# Development of a Generic Physiologically Based Kinetic Model for the Prediction of Internal Exposure to Organophosphate Pesticides

**DOI:** 10.1021/acs.est.4c06534

**Published:** 2024-10-07

**Authors:** Thijs M. J. A. Moerenhout, Jiaqi Chen, Hans Bouwmeester, Ivonne M. C. M. Rietjens, Nynke I. Kramer

**Affiliations:** Division of Toxicology, Wageningen University and Research, Stippeneng 4, 6708 WE Wageningen, The Netherlands

**Keywords:** organophosphate pesticides, PBK modeling, generic PBK model, internal exposure, ADME kinetics

## Abstract

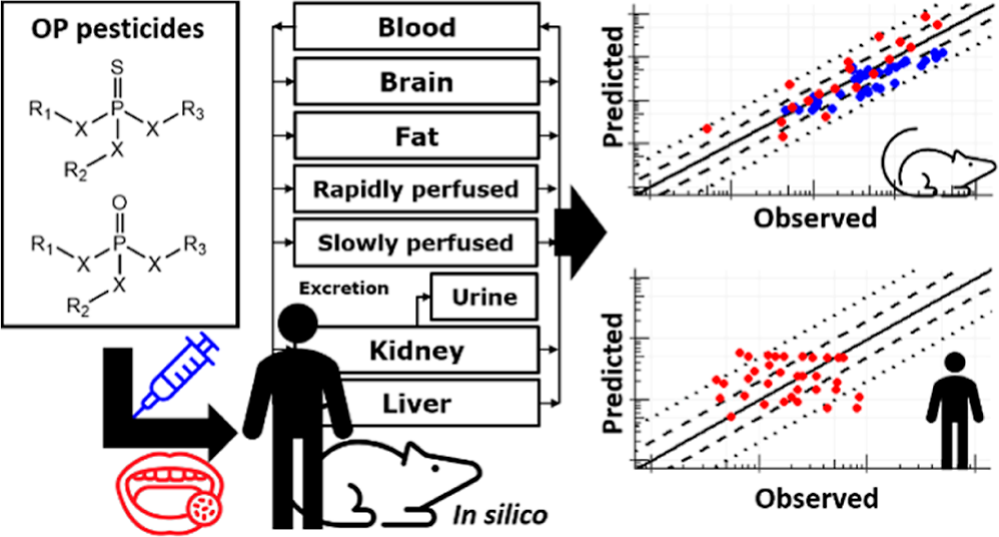

Since their introduction into agriculture, the toxicity of organophosphate (OP) pesticides has been widely studied in animal models. However, next generation risk assessment (NGRA) intends to maximize the use of novel approach methodologies based on in vitro and in silico methods. Therefore, this study describes the development and evaluation of a generic physiologically based kinetic (PBK) model for acute exposure to OP pesticides in rats and humans using quantitative structure property relationships and data from published *in vitro* studies. The models were evaluated using in vivo studies from the literature for chlorpyrifos, diazinon, fenitrothion, methyl-parathion, ethyl-parathion, dimethoate, chlorfenvinphos, and profenofos. Evaluation was performed by comparing simulated and in vivo observed time profiles for blood, plasma, or urinary concentrations and other toxicokinetic parameters. Of simulated concentration–time profiles, 87 and 91% were within a 5-fold difference from observed toxicokinetic data from rat and human studies, respectively. Only for dimethyl-organophosphates further refinement of the model is required. It is concluded that the developed generic PBK model provides a new tool to assess species differences in rat and human kinetics of OP pesticides. This approach provides a means to perform NGRA for these compounds and could also be adopted for other classes of compounds.

## Abstract 2

Using a generic PBK model, rat and human internal concentrations of organophosphate pesticides and their metabolites can be predicted successfully.

## 1 Introduction

**Figure 1:**
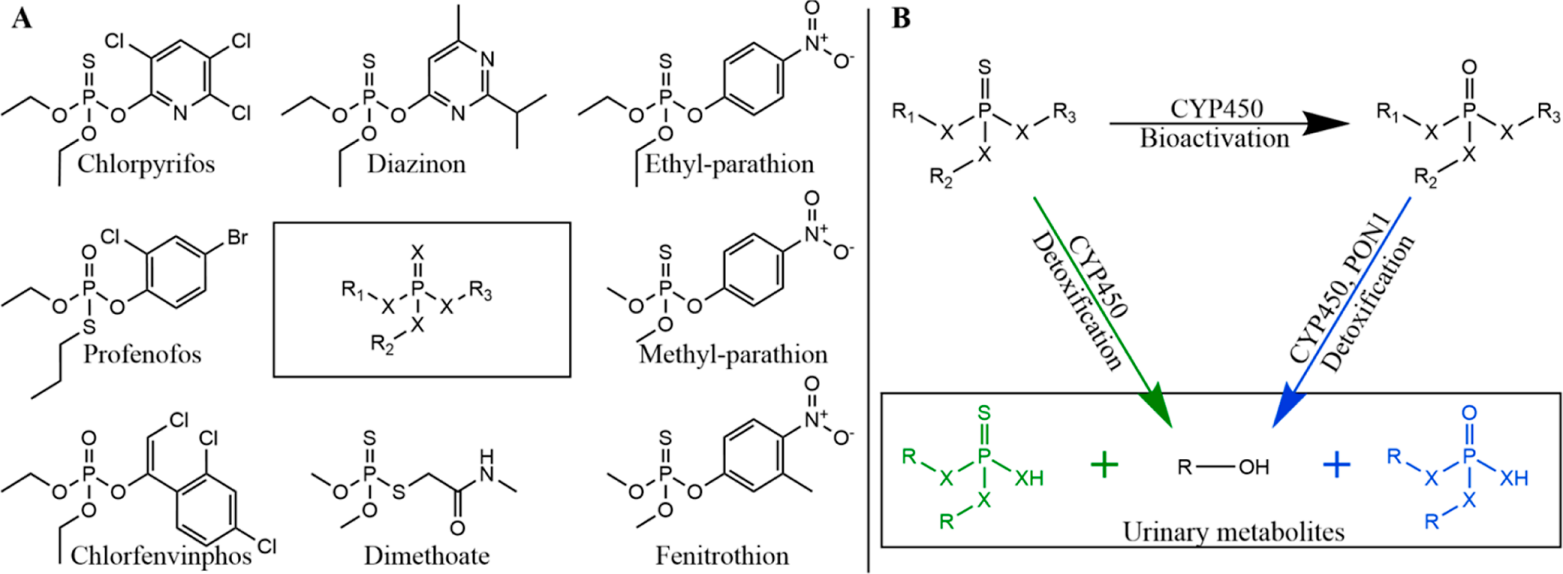
Structures and the generalized metabolic pathway of OP pesticides considered in this study. (A) The central structure represents the general structure for OP pesticides, where X can be a sulfur or an oxygen atom, and usually, no more than two sulfur atoms are bound to the phosphor atom. OTPs and OPOs contain a sulfur or an oxygen atom, respectively, which is connected via a double bond to the central phosphate. R_1_ and R_2_ are alkyl groups, typically methyl or ethyl, and sometimes propyl groups. R_3_ can be aromatic or aliphatic (Supporting Information table S1). (B) Metabolic scheme of OTP and OPO pesticides. In the liver, OTPs can be detoxified to urinary metabolites by CYP450-mediated oxidative cleavage, or they can be bioactivated to OPOs by CYP450-mediated oxidative desulfuration. OPOs can be detoxified to urinary metabolites by CYP450-mediated oxidative cleavage in the liver or hydrolysis by paraoxonase 1 (PON1) enzymes in the plasma and liver.

Although numerous organophosphate (OP) pesticides have been banned in various countries,^1^ OPs are still a widely used class of pesticides.^2^ Two major classes of OP pesticides can be distinguished: organothiophosphates (OTPs) and OP oxons (OPOs; Figure 1). Many OP pesticides are OTPs, which can be bioactivated to OPOs by oxidative desulfuration. Both OTPs and OPOs can also be detoxified by cytochrome P450 (CYP450)-mediated oxidative cleavage to hydrophilic metabolites (Figure 1) that are excreted in urine. The mechanism of action of OP pesticides is based on inhibition of the enzyme acetylcholinesterase (AChE) by OPOs.^3,4^ AChE normally hydrolyzes the neurotransmitter acetylcholine in the synaptic cleft. Inhibition of this enzyme results in an accumulation of acetylcholine in the synapse and subsequent overactivation of the cholinergic nervous system. While blocking of AChE in pest animals is very effective and therefore works well for crop protection, concern has been raised with regard to exposure in nontarget species, including humans. OPs and their metabolites have been repeatedly found in human blood, milk, and urine.^5−9^ In addition, recent studies suggest a correlation between chronic exposure to OP pesticides and neurological complications in the form of neurodegenerative diseases like Parkinson’s and Alzheimer’s disease, possibly mediated by the inhibition of AChE.^10,11^ Together, these studies raise questions about the adequacy of current pesticide regulations and the general safety of these compounds.

In the field of chemical risk assessment, there is a push toward developing and implementing nonanimal test methods or so-called novel approach methodologies (NAMs). The use of these NAMs in risk assessments is termed Next Generation Risk Assessment (NGRA).^12−14^ NAMs include in silico and in vitro approaches aimed at providing a molecular understanding of the kinetics, toxic mode of action, and potency of chemicals. In the last decades, NAMs have also been used for risk assessment of individual OPs.^15−22^ These models were specifically designed for individual OPs, and they have been helpful not only in risk assessment for these specific compounds but also in gaining general understanding of kinetic processes, in pushing for in silico modeling to minimize the reliance on animal experiments, and in elucidating interspecies differences in toxicity. However, to compare kinetics and predict (neuro)toxic potencies of different OPs in rats and humans from in vitro data, a more generic approach would be of great value. This approach is possible since the major metabolic pathways are similar for the OP pesticides previously investigated in the literature (see Figure 1B).^15−17,21,22^ While there are several freely available generic physiologically based kinetic (PBK) models (TK plate,^23^ QIVIVE tools^24^), they do not allow for the incorporation of the metabolism pathways and prediction of concentrations of active metabolites like the oxon forms of the OTPs, as shown in Figure 1. Therefore, a generic model for OP pesticides that is able to incorporate these metabolic pathways will be a useful tool for the risk assessment of this class of compounds.

In the present study, we aim to develop a generic PBK model for OP pesticides that can be used to predict and compare blood and plasma concentrations of OPs and their bioactive metabolites after acute exposures in rats and humans. To ensure adequate accuracy in the prediction of blood concentrations, the model was evaluated using in vivo experimentally obtained rat and human concentration–time profiles in blood and plasma, or urinary excretion profiles found in literature for six OTPs (chlorpyrifos, diazinon, dimethoate, fenitrothion, methyl-parathion, and ethyl-parathion) and two OPOs (chlorfenvinphos and profenofos) (Figure 1) and their metabolites.

## 2 Materials and Methods

### 2.1 PBK Model Development

The PBK models were developed for the simulation of blood and plasma concentrations as well as urinary excretion of OPs and their metabolites in rats and humans after a single intravenous (IV) or oral dose. The rat and human models were evaluated by comparing model simulations with observed in vivo concentration–time profiles in blood, plasma, and urine of rats and humans.

#### 2.1.1 PBK Model Structure

**Figure 2:**
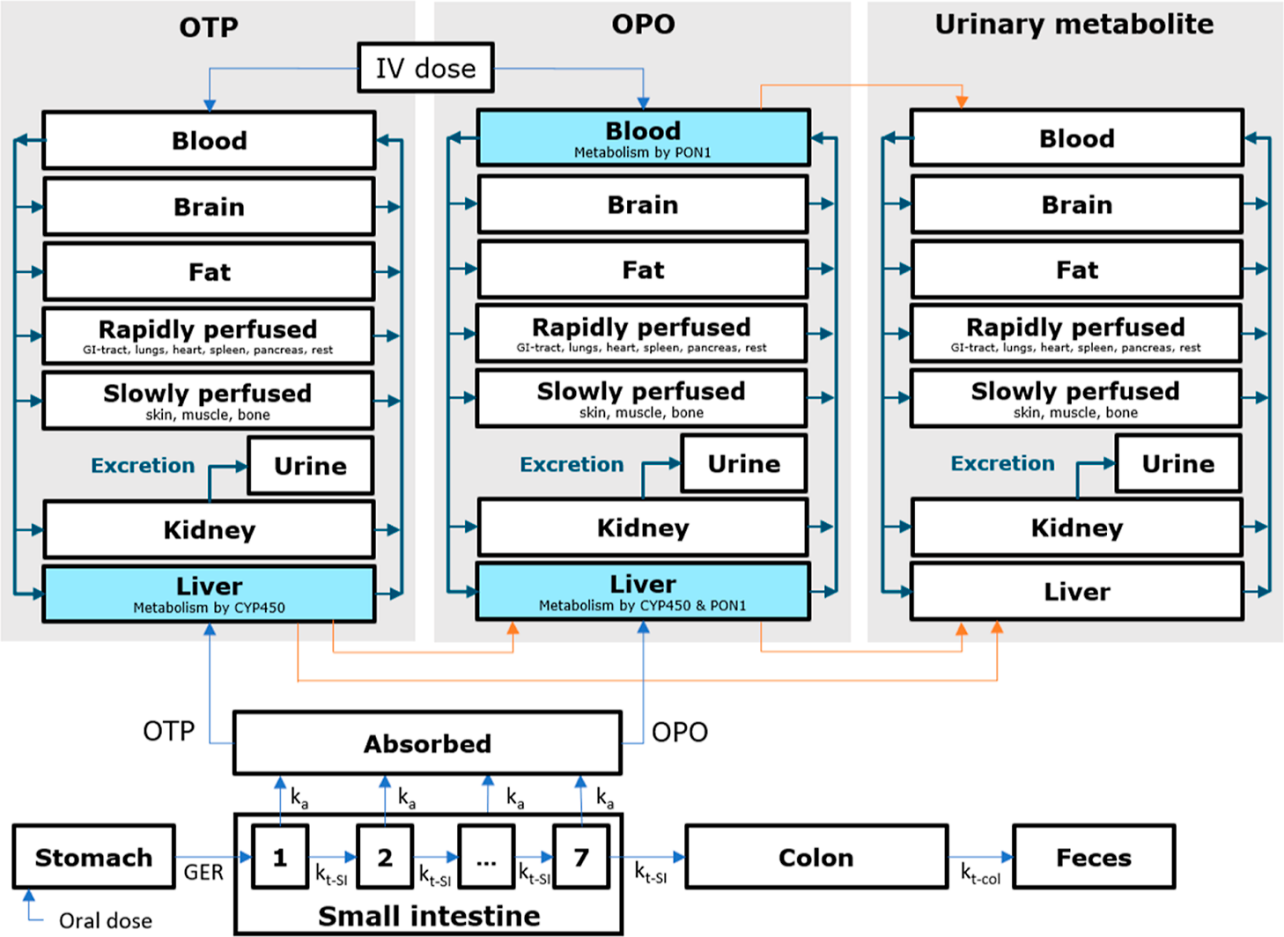
Schematic representation of the PBK model structure. Compounds are absorbed in the small intestine (bottom) and subsequently enter the liver of either the OTP or OPO submodel. Metabolism by CYP450 is incorporated in the liver compartment of both the OTP and OPO submodels. For the OPO submodel, PON1 metabolism is also incorporated in the liver and blood compartments. Urinary excretion by glomerular filtration is incorporated in all submodels. Metabolites from the liver and blood are transferred to their respective submodels. This is represented by orange arrows, while distribution is represented by blue arrows. GER is the gastric emptying rate, *k*_t-SI_ is the transit rate in the small intestine, *k*_a_ is the absorption rate constant, and *k*_t-col_ is the transit time from the colon to feces.

The generic PBK models were developed based on literature describing PBK models for individual OPs^16−19,21,22^ and contain a gastrointestinal absorption model and three submodels (Figure 2; Supporting Information PBK model).

##### 2.1.1.1 Absorption

For the general public, oral exposure via food is considered the main route of exposure to OP pesticides. Therefore, OP oral absorption was modeled using a compartmentalized stomach and intestinal model^25,26^ since it more accurately predicts the temporal absorption profile compared to a mixed-tank model.^25,26^ The model consists of a stomach compartment, seven small intestine (Supporting Information) compartments, one large intestine compartment, and a fecal compartment.^26^ Since most OPs are highly lipophilic, it was assumed that these compounds need bile acids to be dissolved and absorbed. Therefore, OPs were assumed to only be absorbed in the small intestine, where bile acids are active.^27^ All orally absorbed OTP or OPO was assumed to enter the liver, where it can be metabolized or partitioned into the blood. Meanwhile, unabsorbed OPs enter the colon and are excreted in the feces.

##### 2.1.1.2 Distribution

Tissue distribution of OTPs, OPOs, and urinary metabolites was described by their respective submodels, each containing eight compartments representing bodily tissues and urine. Distribution was assumed to be blood flow-limited. Liver and kidney tissues are parametrized as individual compartments since they are clearing organs. The brain is a target organ for OP pesticide toxicity, and fat tissues act as reservoirs for lipophilic OTP and OPO compounds. Therefore, these tissues are modeled separately as well. Skin, muscle, and bone tissues are lumped together in a slowly perfused compartment, and the remaining tissues (lungs, heart, gastrointestinal, and rest) are lumped together in a rapidly perfused compartment. All tissue compartments are connected by a single blood compartment. Distribution between blood and plasma is determined by using the blood-plasma ratio. Urinary metabolites were included in the model since most of the available in vivo data consists of urinary excretion–time data and blood concentration–time profiles for OP urinary metabolites, which were used for model evaluation.

##### 2.1.1.3 Metabolism and Excretion

In the generic PBK model, the main metabolic reactions for the OTPs and the OPOs (Figure 1B) are included. For OTPs, these consist of CYP450-mediated oxidative cleavage to the urinary metabolites and oxidative desulfuration to the bioactive OPOs, both taking place in the liver. PON1 metabolism of OTPs has not been reported (or negligible)^28^ and was therefore not included in the model. For OPOs, hepatic CYP450-mediated oxidative cleavage as well as hepatic and plasma PON1-mediated hydrolysis are included.^29^ Passive renal excretion into the urine was described using a species-specific glomerular filtration rate and a chemical-specific unbound fraction in plasma. When the literature suggested active secretion of conjugates of urinary metabolites, a GFR multiplication factor was included as well, which will be discussed later in the PBK model corrections section.

#### 2.1.2 PBK Model Parametrization

Physiological parameters for adult rats and humans were taken from the literature^30^ and scaled to body weight. For rats, the data are expected to have a tendency toward male physiology.^30^ For human parameters, a tendency toward Caucasian physiology is expected.^30,31^ Passage through the gut model is described by the gastric emptying rate and the small intestinal and colonic transfer rates, respectively.^26,32^ Body weight-corrected cardiac output and glomerular filtration rates were taken from the literature.^33^ Scaling factors for liver and plasma metabolism were obtained from various sources and are reported in Supporting Information table S2, together with all physiological parameters.

##### 2.1.2.1 Compound-Specific Parameters

In this study, eight OP pesticides were used to evaluate the model. Six OTPs: chlorpyrifos, diazinon, fenitrothion, methyl-parathion, ethyl-parathion, and dimethoate; and two OPOs: chlorfenvinphos and profenofos. For these compounds and their metabolites, chemical parameters (log *P* and p*K*_a_) and their sources are shown in Supporting Information table S3. These parameters were utilized in quantitative structure–property relationships (QSPRs) for predicting various compound-specific physicochemical parameters. Given the ease with which parameter values can be generated, QSPR-obtained parameters are preferred over in vitro experiments. Furthermore, many in vitro assays assessing toxicokinetic parameters, such as the apparent permeability (*P*_app_), plasma protein binding, and intrinsic clearance, are poorly amenable to lipophilic organophosphates. There is significant binding to the in vitro apparatus, and standard exposure times are often insufficient (Proença et al., 2021). Therefore, the following approach was used for determining compound-specific parameters for the model: (1) For each compound and metabolite to be simulated, QSPRs (details below) were used for predicting fraction unbound in plasma (fu_p_), blood/plasma ratio (BPR), tissue/plasma partition coefficients, and the absorption rate constant (*k*_a_). (2) Due to the lack of accurate QSPRs for predicting metabolic parameters, all Michaelis–Menten parameters describing compound-specific metabolism were taken from studies that determined these parameters using the in vitro liver microsome, liver cytosol, or plasma incubation assays. In case these parameters were not available in the literature, they were determined in this study or derived based on structural similarity (read-across).

For the determination of tissue/plasma partition coefficients, several QSPRs reported in the literature^37−39^ were considered. For OTPs and OPOs, the QSPR reported by Berezkhovskiy^37^ was used. The QSPR by DeJongh et al. was excluded since it inadequately predicted partition coefficients for ethyl-parathion.^38^ The QSPR by Rodgers and Roland^39^ was only used for urinary metabolites since it was the only QSPR accounting for the charge of a molecule, while the predictive power of this QSPR diminishes for lipophilic compounds with a log *P* > 3.^40,41^ All tissue/plasma partition coefficients were calculated using R code published previously.^42^ The fu_p_ was predicted using the QSPR by Lobell and Sivarajah.^34^ Meanwhile, the BPR was predicted using the SimCyp BPR calculator,^35^ or, in case of accessibility issues, the BPR was set to 1 for neutral compounds and 0.55 for acidic compounds.^36^ In an effort to keep the model as simple as possible, conjugated and unconjugated urinary metabolites were modeled as one. This was done by taking the weighted average of the fu_p_, BPR, and tissue/plasma partition coefficients for each form (glucuronide or sulfate conjugates or unconjugated form) of the urinary metabolite, where for the weights the reported fraction of the respective chemical derivative found in rat urine (Supporting Information table S4) was used. For humans, the same fractions as for rats were used, except for TCP, for which 50% conjugation was used based on the report by Nolan et al.^43^ An example of this method is given in Supporting Information: 1.3 Calculation of physicochemical parameters for urinary metabolites. This enabled the simulation of distribution and excretion of all of the derivatives at once. It must be noted that the current implementation assumes the ratios of different chemical derivatives to be independent of species and dose and that all derivatives are produced in the liver. Since the physiological accuracy of this method is not known, it only gives an estimate of urinary metabolite concentrations and can only be used when the conjugated fractions of the urinary metabolites are available.

Since previous studies showed that microsomal and blood metabolism of OP pesticides followed Michaelis–Menten kinetics,^17,21,22^ the maximum reaction rate (*V*_max_) and the Michaelis–Menten constant (*K*_m_) were used to describe CYP450- and PON1-mediated metabolism in the liver and plasma. These parameters were derived from three sources: in vitro experiments performed in the present study (chlorfenvinphos), read-across (methyl-parathion), and from the literature (all other OPs; Supporting Information table S5). For chlorfenvinphos, microsomal incubations were performed to determine the Michaelis–Menten parameters for CYP450-mediated dealkylation (Supporting Information supporting materials and methods). No plasma incubations were performed since multiple studies show minimal PON1-mediated metabolism of chlorfenvinphos in rats and humans.^44,45^ Since the primary metabolite des-ethyl-chlorfenvinphos was not commercially available, an analytical method for its identification and quantification was developed without the reference compound. Materials and methods for this experiment are described in the Supporting Information. Next, for methyl-parathion, *V*_max_ and *K*_m_ of fenitrothion were used since these compounds only differ by one methyl-moiety in the rest (R3) group. It was assumed that such a small difference at that location on the molecule would not change the metabolism significantly. IV predictions for the parent compound showed good predictions (Supporting Information Figure S18A,C), verifying this assumption. Lastly, the data from the literature were derived using pooled male rat or pooled mixed gender human plasma, microsomal fractions, and, in the case of profenofos, cytosolic fractions (see references in Supporting Information table S5). When implementing metabolism in the model, the *V*_max_ was scaled by tissue weight using scaling factors (Supporting Information table S2 and Supporting Information PBK model), while *K*_m_ was assumed to be similar in vitro and in vivo.

For the parent compounds (chlorpyrifos, diazinon, fenitrothion methyl-parathion, ethyl-parathion, chlorfenvinphos, dimethoate, and profenofos), the absorption rate constant (*k*_a_) was calculated for rats based on a method proposed by Punt et al.^46^ and corrected using ex vivo absorption data for chlorpyrifos.^47^ The associated fa was calculated based on the *k*_a_ and eq 8 from Yu and Amidon^26^ using the transit rate constant (*k*_t_) for the small intestinal transit for rats and humans, respectively (Table S2). The calculated values can be found in the Supporting Information (supporting information calculation of absorption rate constant).

For humans, calculated *k*_a_ values resulted in significant overpredictions of the internal concentrations. Therefore, a *k*_a_ value was derived from fitting the parameter to the urinary excretion of 3,5,6-trichloropyridinol (TCP) in human exposure studies to chlorpyrifos of Timchalk et al. 2002 and Brzak et al. 2000.^19,48^ This resulted in a *k*_a_ of 0.1, which was also used for other OPs in the human model. With this, minimal interindividual differences are assumed. This fixed *k*_a_ approach was used because incorporating the effects of food, formulation, and interindividual differences on absorption into the current model fell outside the scope of this study.

#### 2.1.3 PBK Model Corrections

During development, the model showed some deviations when comparing model simulations to reported in vivo profiles. Some of these deviations could be explained and corrected for. This was done for chlorpyrifos, diazinon, chlorfenvinphos, and the urinary metabolites of fenitrothion and profenofos: 3-methyl-4-nitrophenol (MNP) and 4-bromo-2-chlorophenol (BCP), respectively.

For chlorpyrifos and diazinon, some of the rat oral exposure studies showed a delay in absorption.^19,49^ Therefore, for these simulations, absorption was delayed by the same amount of time as the in vivo data indicated (1–2.5 h; Supporting Information Figures S3, S5, and S10). Furthermore, model simulations of the oral chlorpyrifos exposure studies in humans by Nolan et al.^50^ (Supporting Information figure S6) were corrected by fitting the *k*_a_, as described above (see Section 2.1.2).

Differences between simulated and in vivo profiles of chlorfenvinphos in rats (Supporting Information Figure S16) were attributed to low recovery of the analytical method used in the exposure studies of Ikeda et al.^51,52^ since results from another study^53^ showed that a similar analytical method had low recovery (13%) at 1 μM but less so at higher concentrations. Moreover, Boyer mentioned very strong plasma protein binding for the dimethyl derivative of chlorfenvinphos.^54^ Since protein was not denatured in the analytical method, this explains the low recovery in the aforementioned in vivo studies. Therefore, in vivo blood and plasma concentrations after oral exposure (all below 1 μM) but not following IV exposure (all below 6 μM) were compared to the simulated unbound concentrations instead.

For MNP, BCP, and PNP, all of which are phenol derivatives, literature suggests that phenol glucuronide- and sulfate-phenol conjugates are excreted about 1.6 and 5.5 times faster than the glomerular filtration rate in rainbow trout.^55^ Because these compounds are extensively conjugated (Supporting Information table S4), cross-species extrapolation was used to estimate the active secretion of these urinary metabolite derivatives in rats and humans. The GFR multiplication factors were scaled based on the amount of each conjugate present (see Supporting Information: 1.3 Calculation of physicochemical parameters for urinary metabolites).

Lastly, in vivo data for 2-isopropyl-4-methyl-6-hydroxypyrimidine (IMHP) excretion in urine after exposure to diazinon was assumed to be only free, unconjugated IMHP, since another study reported IMHP being unstable during the acidic hydrolysis step to release IMHP from its conjugate molecule during sample preparation.^49^ Since the human in vivo IMHP data used here were taken from a risk assessment report and the original study was not available in the literature, there was no information available on the specific analytical methods; thus, the assumption could not be checked. The data used for evaluation of the model, reported in the results section, include the discussed corrections.

#### 2.1.4 PBK Model Evaluation

Model performance was evaluated using available in vivo blood and plasma concentration–time and urinary excretion–time profiles after intravenous and oral OP exposures of rats and humans. Importantly, data of intravenous exposures allowed for evaluation of the distribution and clearance predictions without being affected by variation in the absorption process or inaccuracies in the used in vivo studies. Only studies mentioning the route of exposure, the dose, and the (average) bodyweight of the animals/volunteers were considered for evaluation (Supporting Information table S6). Blood and plasma concentration and urine excretion–time profiles for rats and humans were simulated and were compared to observed in vivo profiles. This was done qualitatively, by visually inspecting the overall fit and shape of the simulated concentration–time profile against in vivo profiles and quantitatively, based on observed vs predicted plots and comparison of simulated and in vivo *T*_max_, *C*_max_, and area under the concentration time curve (AUC) values. While the WHO states that model predictions of 2-fold difference from in vivo data are considered good predictions for compound-specific models,^56^ guidelines for generic models are lacking. According to Abduljalil et al., high variability in observed studies with small sample sizes can have a significant effect on interstudy variation in pharmacokinetic data.^57^ Furthermore, in classic risk assessment, a higher uncertainty factor than 2-fold, being 3.2, has already been used for a long time to account for intraspecies differences in kinetics.^58^ Lastly, Punt et al.^59^ also found that a 2-fold difference is not well applicable to generic models, suggesting to use a 5-fold difference as a goodness-of-fit criterion but also indicated that better measures are needed. For these reasons, predictions were considered adequate when simulated blood and plasma concentration–time or urinary excretion–time profiles were within a 5-fold difference from the respective in vivo profiles, and the overall shape was visually similar.

#### 2.1.5 Sensitivity Analysis

Using the FME R package,^60^ a local sensitivity analysis was performed to determine the impact of individual parameters on the simulated maximum free concentration of OPO in blood, which is related to the inhibition of AChE and the consequent acute neurotoxicity following OP exposure. Sensitivity coefficients (SC) were calculated following the formula: ,^60^ where *C* is the maximum free concentration of OPO in blood and P is the model parameter value. Input parameters were changed by 1%, and the relative effect on the maximum free concentration of the OPO in blood was reported. Sensitivity analysis was performed using a single oral dose of 1 mg/kg of body weight for both rats and humans. This dose was selected to represent a dose that is close to doses used in both rat and human in vivo studies. At this dose, concentrations relevant for metabolism were all below the *K*_m_ and therefore in the linear range of the saturation curve. Furthermore, using the same dose also allows for a better comparison between the two species.

### 2.2 Software and Data Analysis

The R programming language (v 4.1.0) and R studio (v 1.4.1717) were used to develop the model. Several packages were used for modeling and data analysis. The *RxODE2* package^61^ was used to solve the ordinary differential equations of the model. *Tidyverse* and *dplyr* were used for data handling, and *Readxl* and *xlsx* were used to read and write Excel files for input and output, respectively. The *FME* package was used for the sensitivity analysis. Microsoft Excel (version 16.0.14931.20764) and GraphPad Prism (version 5.04), and the R packages *ggplot2* and *patchwork* were used to analyze and visualize the data. GraphPad Prism was also used to derive the Michaelis–Menten parameters from data from incubation studies. WebPlotDigitizer (https://automeris.io/WebPlotDigitizer/) was used to extract in vivo concentration–time data from graphs in the literature.

## 3 Results

To develop the PBK model, physiological, chemical, and metabolic data are required. Physiological and chemical data were retrieved from the literature or predicted using QSPRs (Supporting Information tables S2 and S3). Metabolic parameters were available in the literature for all OPs (Supporting Information table S5) except for chlorfenvinphos, for which the metabolic parameters were determined experimentally in the present study.

### 3.1 Chlorfenvinphos Metabolic Incubation Experiments

Microsomal incubations were performed with both rat and human liver microsomes to define the PBK model parameters for the hepatic clearance of chlorfenvinphos. Des-ethyl-chlorfenvinphos was the only metabolite detected in incubations. It was not produced if the incubations were performed without reduced nicotinamide adenine dinucleotide phosphate. Parameters describing the Michaelis–Menten kinetics of chlorfenvinphos metabolism were 0.46 and 0.57 nmol/min/mg microsomal protein for *V*_max_ and 5.91 and 6.36 μM for the *K*_m_ for rat and human liver microsomes, respectively (Supporting Information figure S1). The catalytic efficiencies (calculated as *V*_max_/K_m_) were 78.0 and 88.9 μL/min/mg microsomal protein for rat and human liver microsomes, respectively. The *V*_max_ and *K*_m_ were used to describe the metabolism of chlorfenvinphos in the human and rat models.

### 3.2 PBK Model Evaluation

Once all parameters for all eight OP pesticides (chlorpyrifos, diazinon, fenitrothion, methyl-parathion, ethyl-parathion, dimethoate, chlorfenvinphos, profenofos, and metabolites) were collected, the generic model was developed (Supporting Information PBK model) and evaluated. The model was found to have an adequate mass balance, with the mass error of around 1 × 10^–12^ μmol for runs using doses of 1 to 100 mg/kg body weight. Very low doses of 0.001 mg/kg bodyweight gave mass errors of 1 × 10^–9^ μmol (data not shown), which are considered acceptable.

**Figure 3:**
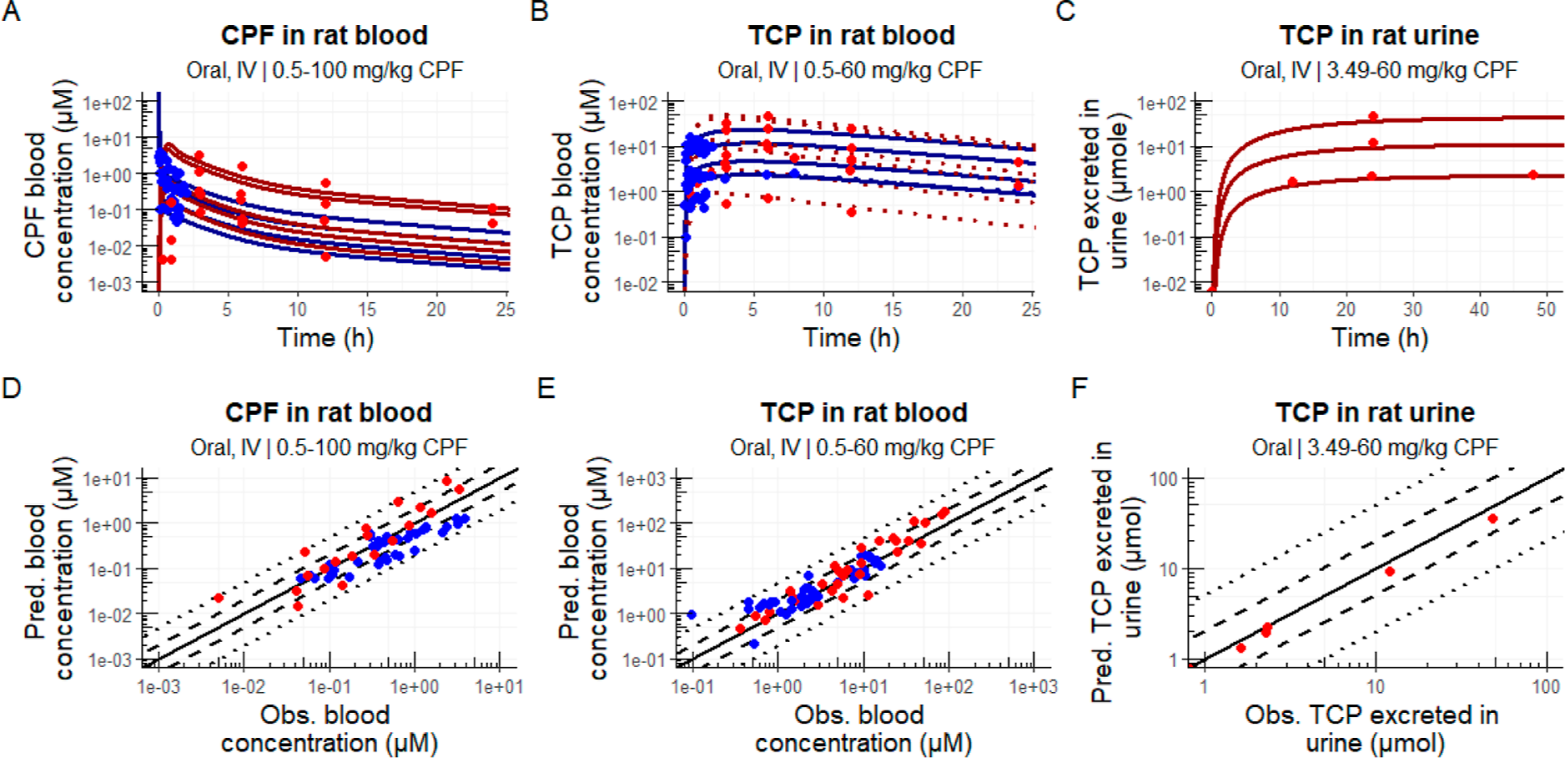
Simulated blood concentration- and urinary excretion–time profiles (A–C) and predicted versus observed data plots (D–F) for chlorpyrifos exposures in rats. Blue and red dots represent means of observed in vivo values after intravenous or oral doses, respectively, and similarly, colored solid and dotted lines represent simulated profiles (at different doses) of the total and unconjugated blood concentration, respectively, depending on the reported concentration in vivo. Unconjugated concentrations were based on conjugate ratios reported in Supporting Information table S4. In predicted versus observed data plots (D–F), lines representing perfect prediction (solid line), 2-fold difference (dashed lines), and 5-fold difference (dotted lines) are shown in black. See Materials and Methods for included corrections. The reader is referred to Figures S2–S8 for more detailed comparisons of each individual exposure. Abbreviations: CPF: chlorpyrifos; TCP: 3,5,6-trichloropyridinol.

**Figure 4:**
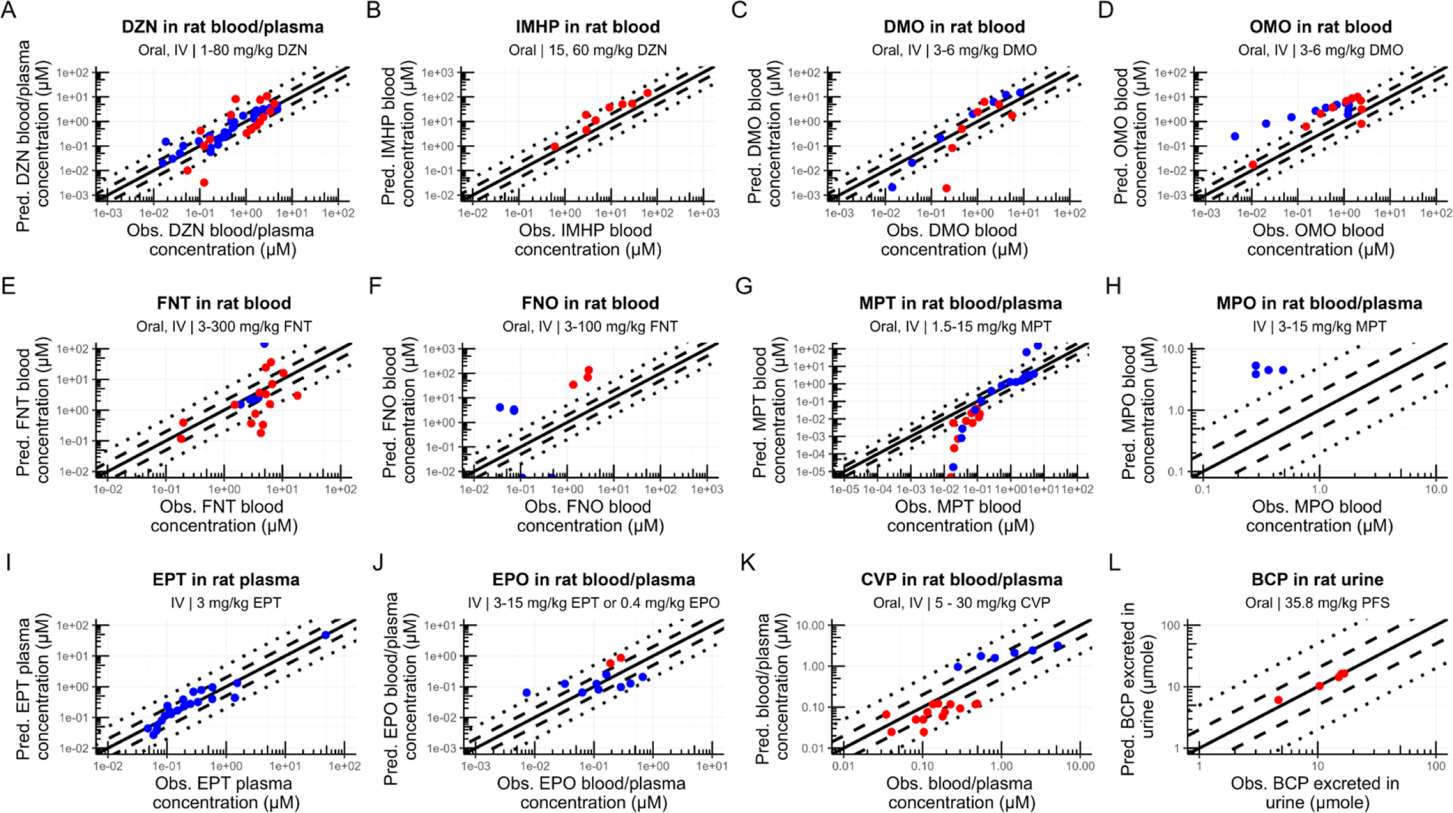
Predicted versus observed data plots for oral (red) and intravenous (blue) OP exposures in rats. Rats were exposed to diazinon (DZN; A,B), dimethoate (DMO; C,D), fenitrothion (FNT; E,F), methyl-parathion (MPT; G,H), ethyl-parathion (EPT; I,J), chlorfenvinphos (CVP; K), and profenofos (PFS; L). Lines for perfect prediction (solid line), 2-fold difference (dashed lines), and 5-fold difference (dotted lines) are shown in black. See Materials and Methods for included corrections. The reader is referred to the Supplementary Figures S9–S19 for more detailed comparisons of each individual exposure. Metabolite abbreviations: IMHP: 2-isopropyl-4-methyl-6-hydroxypyrimidine; OMO: omethoate; FNO: fenitro-oxon; MPO: methyl-paraoxon; EPO: ethyl-paraoxon; and BCP: 4-bromo-2-chlorophenol.

**Figure 5:**
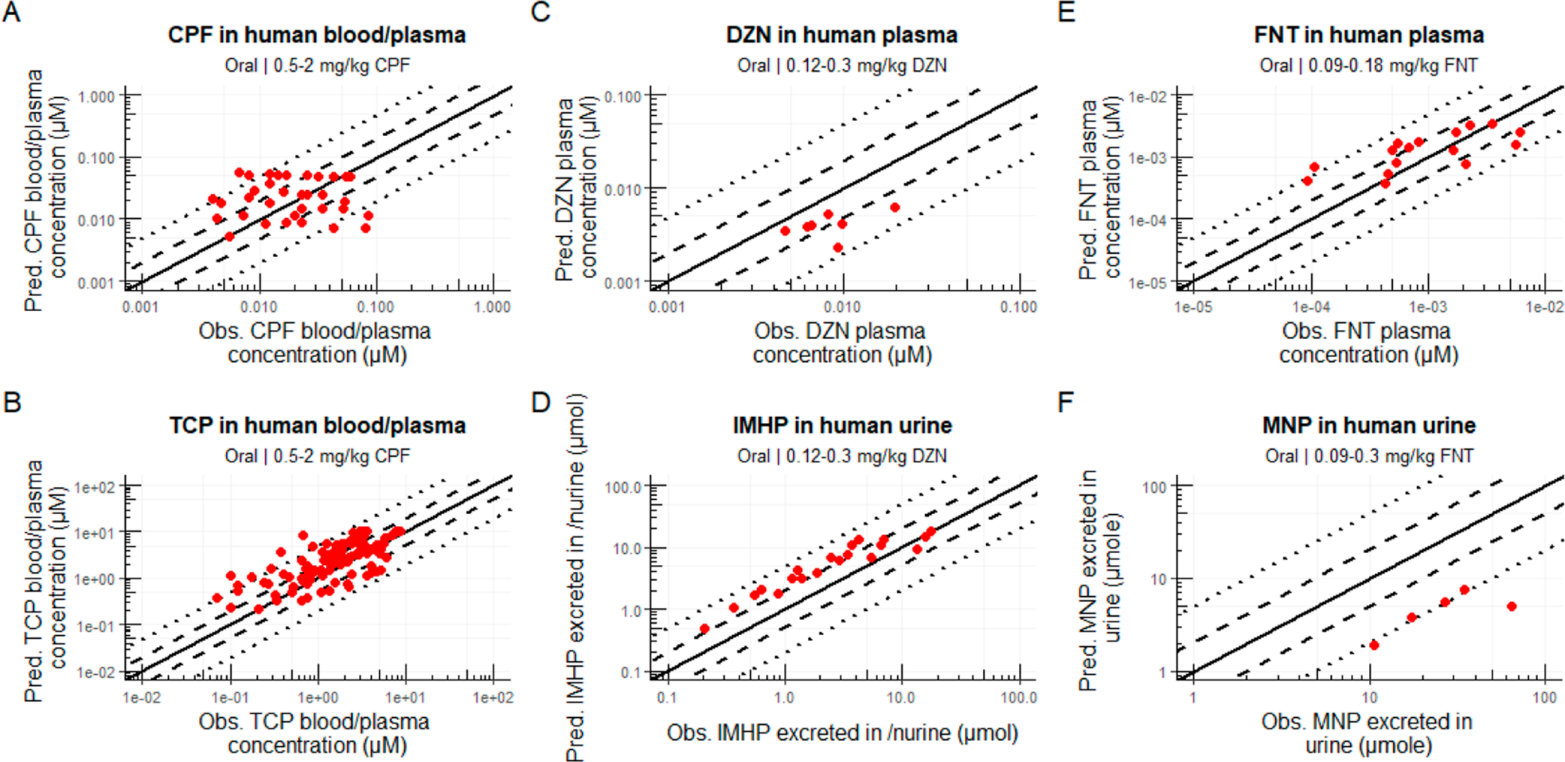
Predicted versus observed data plots for oral exposure to OPs in humans. Humans were exposed to chlorpyrifos (CPF; A,B), diazinon (DZN; C,D), and fenitrothion (FNT; E,F). Lines for perfect prediction (solid line), 2-fold difference (dashed lines), and 5-fold difference (dotted lines) are shown in black. See Materials and Methods for included corrections. The reader is referred to Figures S6–S8, S11, and S14 for more detailed comparisons of each individual exposure. Metabolite abbreviations: TCP: 3,5,6-trichloropyridinol; IMHP: 2-isopropyl-4-methyl-6-hydroxypyrimidine; and MNP: 3-methyl-4-nitrophenol.

The studies considered for model evaluation are listed in Supporting Information table S6. The PBK model was evaluated by means of comparison of concentration–time plots (Figure 3A–C), by plotting observed versus predicted concentrations (Figures 3D–F, 4, and 5), and by comparison of predicted and in vivo observed toxicokinetic parameters (*T*_max_, *C*_max_, AUC; Supporting Information table S7). Data used for these comparisons included literature reported in vivo rat and human blood and plasma concentration–time profiles and urinary excretion profiles after intravenous and oral exposures. Toxicokinetic parameters were calculated for model simulations as well as in vivo data that consisted of four or more data points. All simulated exposures are graphed individually in Supporting Information Figures S2–S19, to provide more information on the temporal differences and the shape of the predicted curve for individual compounds and exposures.

#### 3.2.1 Simulations for OP Exposures in Rats

Simulations of OP exposures in rats were evaluated using predicted versus observed plots (Figures 3D–F and 4). In vivo data were well predicted by the rat model, with the exception of omethoate, fenitro-oxon, and methyl-paraoxon. For all simulations combined, 85 and 55% of simulated concentrations fell within 5-fold and 2-fold differences from in vivo data, respectively. These percentages were higher for IV (87 and 62%) than for oral exposures (82 and 46%). For each compound individually, all of the diethyl-organophosphates were predicted, with at least 85% of predicted in vivo blood and plasma concentrations falling within a 5-fold difference when compared with corresponding in vivo data for IV exposures. This was 80% for oral exposures. However, for dimethyl-organophosphate-oxons, predictions were less accurate. For fenitro-oxon and methyl-paraoxon, all predictions fell outside the 5-fold difference from in vivo data. Meanwhile, for omethoate, only 40% fell within a 5-fold difference. The parent compounds (OTPs) of the dimethyl-organophosphates were better predicted, with 67 and 36% predicted within 5- and 2-fold difference.

Next, simulated and in vivo observed toxicokinetic parameters (*T*_max_, *C*_max_, and AUC) were compared (Supporting Information table S7). All but one of the predicted *T*_max_ values were predicted within a 5-fold difference. Furthermore, for 37 out of 40 data sets, in vivo *C*_max_ and AUC values were predicted within a 5-fold difference from in vivo data. The three data sets for which the predicted *C*_max_ and AUC fell outside a 5-fold difference were for chlorpyrifos, diazinon, and methyl-parathion. Predictions with a large fold difference compared to in vivo data were often explained by the low number of in vivo data points in the experimental concentration time curve.

#### 3.2.2 Simulations for OP Exposures in Humans

The rat model was adjusted using human parameters and used to simulate human blood and plasma concentration–time profiles and urinary excretion–time profiles after oral exposures. Human in vivo exposure data were only available for oral exposures to chlorpyrifos, diazinon, and fenitrothion. Therefore, these compounds were used for evaluation of the human model.

Figure 5 shows that human data were well predicted, with 91 and 43% of simulated concentrations being predicted within 5- and 2-fold differences from in vivo data, respectively. For chlorpyrifos, TCP, diazinon, IMHP, and fenitrothion individually, each was predicted with more than 80% of the predicted blood and plasma concentrations and urinary excretion within 5-fold difference from in vivo data. Meanwhile, 60% of the predicted amounts of MNP excreted in urine fell within the 5-fold difference limit.

Predicted toxicokinetic parameters could only be compared to those of TCP and fenitrothion due to data availability. For TCP, the *T*_max_ was well predicted with a 1.1-fold difference compared to in vivo data. Meanwhile, the predicted *C*_max_ and AUC were 3.19- and 2.45-fold different from the in vivo data, respectively. Similar accuracy was found for fenitrothion: *T*_max_, *C*_max_, and AUC were predicted with 1.1-, 2.3-, and 3.3-fold differences from in vivo data, respectively.

When comparing the model predictions of chlorpyrifos, diazinon, and fenitrothion in rats and humans, it shows that the approach seems to have a similar accuracy for both species for chlorpyrifos and diazinon exposures. Meanwhile, fenitrothion as a parent compound seems to be reasonably predicted in both rats and humans, while its urinary metabolite is overpredicted in rats and significantly underpredicted in humans, suggesting that there might be species-specific differences for this compound. Furthermore, exposure to 1 mg/kg bw of each pesticide to both rats and humans was simulated to investigate species differences between rats and humans. The largest species differences in blood *C*_max_ were found for chlorpyrifos-oxon, ethyl-parathion, omethoate, and chlorfenvinphos (see Supporting Information table S8).

### 3.3 Sensitivity Analysis

SC were calculated to quantify the effect of individual parameters on the predicted maximum free blood concentration of the OPOs. Besides body weight, the most sensitive parameters were related to metabolism, absorption or blood flow, and blood partitioning (Supporting Information Figures S20–27). Overall, for the generic human model, the predictions for diazinon, ethyl-parathion, and chlorpyrifos show the highest number of sensitive parameters, with 17, 13, and 11 input parameters with a sensitivity coefficient of 0.5 or higher, respectively. Furthermore, when comparing the SC of rats and humans, diazinon showed the least and fenitrothion the most interspecies differences in sensitivity analysis. When sensitivity and uncertainty are compared according to the WHO PBK characterization guidelines^56^ (see Supporting Information 1.4 sensitivity/uncertainty matrix), it is clear that the *k*_a_ is of importance since it is highly sensitive and also has high uncertainty associated with it.

## 4 Discussion

The aim of this study was to develop a generic PBK model for OP pesticides, which can be used to predict and compare blood concentrations of OPs and their bioactive metabolites after acute exposures in rats and humans. The model was evaluated for rats and humans using the respective available in vivo data for eight OP pesticides. When considering all simulations, the rat model was shown to predict 85 and 55% of the simulated blood and plasma concentrations within a 5- and 2-fold difference from in vivo data, respectively. For humans, these values were 91 and 43%, respectively, when considering only simulations for chlorpyrifos, diazinon, and fenitrothion. Similar trends were seen for the predicted toxicokinetic parameters. With these results, the model was considered to adequately predict blood and plasma concentrations of OP pesticides and their metabolites, with the exception of predictions for the dimethyl-organophosphates dimethoate, methyl-parathion, and fenitrothion. Looking at individual pesticides, the blood and plasma concentration–time and urinary excretion–time profiles, and toxicokinetic parameters were well predicted for chlorpyrifos, diazinon, ethyl-parathion, chlorfenvinphos, profenofos, and their metabolites. While for dimethoate, fenitrothion, and methyl-parathion rat blood and plasma concentration–time and urinary excretion–time profiles and toxicokinetic parameters were well predicted, simulated blood and plasma concentrations of their oxon metabolites differed by more than 5-fold from in vivo data. In humans, however, the fenitrothion exposures were well predicted within a 5-fold difference compared to the available in vivo data. While many exposures were predicted well by the final model, initial simulations revealed that some corrections were needed.

Some of the corrections were needed due to errors in the analytical methods used in the in vivo studies, like those mentioned earlier for chlorfenvinphos (see Section 2.1.3). The quality of reporting of in vivo data affected the reliability of some specific data sets. Namely, plotting concentration–time profiles nonlogarithmically hampered accurate extraction of data for time points that are close to the *x*-axis, as was the case for methyl-parathion and dimethoate (Figure 4C,D,G). Since this could not be corrected for, low concentrations of in vivo data for these compounds are less reliable.

Further corrections were related to physiological aspects and were especially relevant for predicting data upon oral exposure since there were some odd trends in *in vivo* oral exposure data, especially in rats. First, in some studies a delay in oral absorption was visible (Supporting Information Figures S3, S5, and S10), while in default simulations absorption starts immediately after exposure. Predictions were improved when incorporating a 1 to 2.5 h delay in absorption. In rats, such a delay in absorption might be explained by the presence of a forestomach, which is able to store food for up to 3 h.^62^ Second, while the current model accounts for a temporal aspect of absorption by simulating transit through the intestine, differences are found in the *k*_a_ between in vivo studies. In this study, the *k*_a_ was predicted using a set of QSPRs,^46^ in combination with intestinal permeability data^47^ (Supporting Information: Calculation of absorption rate constant). For rats, these predictions worked very well for those studies that used oil as the dosing vehicle, while for humans, the predicted *k*_a_’s resulted in overpredictions of blood and plasma concentration–time profiles. It is often quite clear that when the pesticides were dosed with food or oils from biological origin, a high *k*_a_ was found, while absorption seems to be lower and slower when synthetic oils were used. For human studies, this is apparent when comparing the studies of Nolan et al.,^50^ Timchalk et al.,^19^ and Brzak et al. 2000 (via EPA, 2009). In the study of Nolan et al., dosing was accompanied by a meal, and a *k*_a_ of 0.6 was found after fitting to in vivo urinary excretion of TCP, while in the study of Timchalk et al., there was no mentioning of food or fasting state, and the *k*_a_ was found to be around 0.1. If these volunteers in the latter study indeed did not eat when ingesting chlorpyrifos, food effects facilitating the oral absorption of these lipophilic compounds may explain the difference between these studies. In this study, it is hypothesized that the higher bioavailability of OPs, when ingested with food, is mediated by bile acids. Due to their lipophilicity, OP pesticides easily pass the membranes of the intestinal wall. However, these compounds are poorly soluble in intestinal fluids, making dissolution the rate-limiting step in intestinal absorption. Bile acids significantly increase the solubility of lipophilic compounds and, therefore, increase the rate at which they are absorbed. Bile acids are released when free fatty acids are detected in duodenal fluids, which are induced by the presence of food or oils of biological origin in the intestinal lumen. While humans have a gallbladder that releases bile when needed, rats do not have this organ and continuously secrete bile.^63^ This in itself might, in part, explain why the *k*_a_ for intestinal absorption of OPs in rats is generally higher than that in humans. However, according to Vonk et al.,^64^ when rats are fasted, bile secretion rates are about half of those in a fed state, potentially affecting the *k*_a_ for intestinal uptake. Implementing bile-mediated intestinal dissolution is therefore expected to improve predictions, especially in humans. Measuring the dissolution profile in fasted and fed gastro-intestinal fluids is considered to be of high importance for improving PBK predictions for lipophilic compounds since, as was shown in the sensitivity/uncertainty matrix (Supporting Information materials and methods 1.4), the *k*_a_ is a highly sensitive parameter with high uncertainty.

While *k*_a_ is the main absorption parameter in the model, if it is derived from in vivo data, it represents various physiological and kinetic processes that take place in the intestine. Not all of these processes might be captured using in vitro or QSPR models. This includes distribution by chylomicrons through the lymphatic system. Once absorbed, lipophilic compounds might be distributed via the lymphatic system, packaged in chylomicrons and lipoproteins since these particles have a lipid core.^65^ While fatty acids will be extracted from these particles by various tissues, the particle itself, its lipophilic core, and potentially OP pesticides will almost completely be taken up by the liver and the spleen as a chylomicron remnant particle.^66,67^ This is different from more hydrophilic compounds, which are usually distributed via the portal vein after absorption and for which passive diffusion into the liver is generally quite low due to their hydrophilicity. Currently, to our knowledge, there is no reported in vitro or in silico method to quantitatively predict the extent to which a compound is distributed via chylomicrons after oral absorption. Such a method could potentially improve PBK predictions for lipophilic compounds since these differences in distribution after absorption influence the concentration in the liver and therefore the extent of metabolism.

Metabolic clearance was predicted well for five of eight of the modeled compounds. Omethoate, fenitro-oxon, and methyl-paraoxon blood concentrations were predominantly predicted with over a 5-fold difference from in vivo data. This difference might be explained by an additional metabolic process for dimethyl-OPs that is not included in the model: glutathione transferase-mediated *O*-dealkylation. Several studies suggest that glutathione transferases, located in the soluble fraction of the liver, are able to extensively cleave the alkyl groups (usually R_1_ and R_2_ in Figure 1 and Supporting Information table S1) of dimethyl-organophosphates.^68−70^ This might explain the overpredictions of the oxon metabolites and urinary metabolites of dimethyl-organophosphates like dimethoate, fenitrothion, and methyl-parathion. Addition of metabolism and submodels for dealkylated metabolites would likely make the model more accurate for dimethoate, fenitrothion, and methyl-parathion but also increase model complexity.

Overall, the presented generic PBK models for rats and humans were shown to predict OP pesticide and metabolite concentrations in blood, plasma, and urine within generally a 5-fold and often even 2-fold difference from in vivo data. Deviations could be explained by lack of dealkylation metabolism in the models and food/oil effects on absorption. The evaluated model can be used to simulate blood and plasma concentrations for OP pesticides that are metabolized via CYP450-mediated desulfuration, oxidative cleavage, and PON1-mediated hydrolysis. With regard to NGRA, to remove all reliance on animal experiments, the model could be further combined with in vitro or in silico absorption models to determine the *k*_a_ for a specific OP formulation. The general approach described in this study could also be applied to other classes of compounds, providing further insights into their toxicokinetics and aiding in NGRA. In a future study, the model will be used for quantitative *in vitro* to *in vivo* extrapolation (QIVIVE) to determine PODs for acute neurotoxicity after exposure to OP pesticides, supporting animal alternative risk assessment and providing tools for elucidating the association of OP exposure and neurodegenerative diseases.

## Supporting Information Available

Materials and methods for chlorfenvinphos in vitro metabolism incubations and its analytical analysis, absorption rate constant (*k*_a_) calculation, calculation of physicochemical parameters for urinary metabolites, sensitivity/uncertainty matrix; summary of OP description, physiological, physicochemical, and kinetic parameters and urinary metabolite ratios; summary of in vivo studies used for model evaluation, toxicokinetic parameters, and species differences; chlorfenvinphos metabolic incubations, sensitivity analyses, and concentration profiles for each individual exposure for each study; and PBK model code and mass balance equations (PDF)
