# Correction to “Development of a Generic Physiologically Based Kinetic Model for the Prediction of Internal Exposure to Organophosphate Pesticides”

**DOI:** 10.1021/acs.est.4c11675

**Published:** 2024-11-04

**Authors:** Thijs M. J. A. Moerenhout, Jiaqi Chen, Hans Bouwmeester, Ivonne M. C. M. Rietjens, Nynke I. Kramer

In supplementary Table S5, showing the metabolic parameters, the *V*_max_ and *K*_m_ values for rats and humans were swapped for the conversion of diazinon to diazinon–oxon. The correct values are given here and in the attached Supporting Information.

**Table S5:** 

	Rat		Human	
	*V*_max_ (nmol/min/mg prot)	*K*_m_ (μM)	*V*_max_ (nmol/min/mg prot)	*K*_m_ (μM)
DZN > DZO	0.288	4.745	0.187	59.6

## Supporting Information Available

Materials and methods for chlorfenvinphos in vitro metabolism incubations and its analytical analysis, absorption rate constant (*k*_a_) calculation, calculation of physicochemical parameters for urinary metabolites, sensitivity/uncertainty matrix; summary of OP description, physiological, physicochemical, and kinetic parameters and urinary metabolite ratios; summary of in vivo studies used for model evaluation, toxicokinetic parameters, and species differences; chlorfenvinphos metabolic incubations, sensitivity analyses, and concentration profiles for each individual exposure for each study; and PBK model code and mass balance equations (PDF)

