# Supplementary material for: Development of a Generic Physiologically Based Kinetic Model for the Prediction of Internal Exposure to Organophosphate Pesticides
Source: Environ Sci Technol. 2024 Oct 7;58(42):18834–45. doi: 10.1021/acs.est.4c06534 (PMC11500413; doi:10.1021/acs.est.4c06534)
Supplement: Supplementary file 1 — es4c06534_si_001.pdf [file es4c06534_si_001.pdf]

Supporting Information

Development of a generic physiologically based kinetic model for the prediction of internal exposure to organophosphate pesticides

Thijs M.J.A. Moerenhout[1], Jiaqi Chen[1], Hans Bouwmeester[1], Ivonne M.C.M. Rietjens[1], Nynke I. Kramer[1]*

1. Division of Toxicology, Wageningen University and Research, Stippeneng 4, 6708 WE Wageningen, The Netherlands

* corresponding author at thijs.moerenhout@wur.nl

Figures: 27

Tables: 10

Pages: 51

Table of Contents

1. Supporting Materials and Methods 3
   1.1 Chlorfenvinphos metabolism studies 3
   1.2 Calculation of absorption rate constant 4
   1.3 Calculation of physicochemical parameters for urinary metabolites 5
   1.4 Sensitivity/uncertainty matrix 6
2. Supporting Tables 7
   Table S1: Organophosphate pesticides and their rest groups 7
   Table S2: Physiological parameters 7
   Table S3: Physico-chemical parameters for organophosphate pesticices 8
   Table S4: Metabolite ratios in urine 10
   Table S5: Metabolic parameters. 11
   Table S6: Studies used for model evaluation 13
   Table S7: Simulated and observed toxicokinetic parameters for organophosphate exposures 15
   Table S8: Species differences between rats and humans 17
3. Supporting Figures 18
   3.1 Chlorfenvinphos metabolism kinetics 18
   3.2 Simulated and measured concentration-time profiles 19
      3.2.1 Chlorpyrifos 19
      3.2.2 Diazinon 26
      3.2.3 Fenitrothion 29
      3.2.4 Profenofos 32
      3.2.5 Chlorfenvinphos 32
      3.2.6 Dimethoate 33
      3.2.7 Methyl-parathion 34
      3.2.8 Ethyl-parathion 35
   3.3 Sensitivity analysis 36
4. PBK model 40
   4.1 General abbreviations in model 40
   4.2 Model parameter and variable name list 40
   4.3 Mass balance equations 44
   4.5 Model Code 45
5. References 50

1. Supporting Materials and Methods

1.1 Chlorfenvinphos metabolism studies

To develop the model, physiological, chemical and metabolic data are required. For chlorfenvinphos, the metabolic parameters were not available in literature. Therefore, the metabolic parameters for chlorfenvinphos were determined experimentally. Here the experiments used to determine these parameters are described.

Chemicals and materials

Chlorfenvinphos (>96.7%), 1-(2',4'-dichlorophenyl)ethanol (DPE), 2,2′,4′-trichloroacetophenone (TCAP; 97%), tris(hydroxymethyl)aminomethane (Tris-HCl; Trizma® base 99.99%), tetraisopropyl pyrophosphoramide (iso-OMPA), trifluoroacetic acid (TFA; >99%), ethylenediaminetetraacetic acid (EDTA; >98.5%) and magnesium chloride ($MgCl2•6H2O$; 99.99%) were ordered from Sigma-Aldrich (Amsterdam, The Netherlands). Reduced nicotinamide adenine dinucleotide phosphate (NADPH; >95%) was ordered from Carbosynth Ltd. (Compton, UK). Acetonitrile (CAN; 99.97%) was ordered from Biosolve (Valkenswaard, The Netherlands). Pooled male Sprague Dawley rat liver microsomes and mixed gender pooled human liver microsomes (150 donors) were ordered from Corning (Amsterdam, The Netherlands).

Microsomal incubations

Conditions for human and rat liver microsomal incubations were optimized with regard to linearity of metabolite formation with time and microsomal protein concentration (data not shown). Optimized incubation conditions were the same for rats and humans. Incubations were performed in glass tubes (~8mL borosilicate glass test tubes) in 50 mM Tris-HCl (pH = 7.4) with final concentrations of 50 µM iso-OMPA, 5 mM EDTA, 5 mM MgCl2, 1 mM NADPH and 5-200 µM chlorfenvinphos. Iso-OMPA and EDTA were added to inhibit A- and B-esterase activity, while MgCl2 and NADPH were added as the cofactor for cytochrome P450 (CYP450)-mediated metabolism. Chlorfenvinphos stock solutions were prepared in 100% acetonitrile and the concentration of acetonitrile in the final incubation mixture was 1% (v/v). Control incubations, with the same concentration of chlorfenvinphos as in the treatment incubations, were incubated without the addition of the NADPH cofactor. Mixtures without microsomes were pre-incubated in a 37°C water bath for 1 minute, after which the incubation was started with the addition of rat or human liver microsomes (final concentration 0.05 mg protein/mL), making the total volume of the reaction mixture of 200 µL. Incubations were stopped after 10 minutes with the addition of 100 µL ice cold acetonitrile and put on ice. Tubes were centrifuged at 4000 g for 15 minutes at 4°C and the supernatant was analysed on a UPLC-UV/vis system.

Detection and quantitative analysis

A Shimadzu Nexera X2 UPLC system (2 LC-30AD pumps, SIL-30AC autosampler and a CTO-20AC column oven, Japan) equipped with a Kinetex C18 100Å LC column (50mm × 2.1mm, 1.7µm; Phenomenex, US) with a SecurityGuard ULTRA UHPLC C18 guard column (2.1mm; Phenomenex, US). A SPD-M20A PDA (Shimadzu, Japan) for UV/vis absorption detection was used to detect chlorfenvinphos, and its metabolites TCAP and DPE. A binary gradient of 0.1% TFA in pure water as the polar mobile phase and ACN as the apolar phase was used. The gradient started at 10% ACN, increased to 100% ACN for 20 minutes after which 100% ACN was kept for 5 minutes. Next, the concentration ACN was brought back to 10% in 30 seconds and kept at that concentration for 4.5 minutes. A flow of 0.6 ml/min was used and the column oven was set at a constant temperature of 40°C. 20µL of samples was injected. Chlorfenvinphos and its metabolites TCAP and DPE had retention times of 8.9, 7.25 and 5.9 minutes, respectively. The limit of detection was 0.1 µM for chlorfenvinphos and 0.5 µM for DPE. An unidentified peak was found at the retention time of 4.1 minutes, of which the area changed depending on chlorfenvinphos concentration, microsomal concentration and incubation time,

and was investigated using a micrOTOF TOF-MS (Bruker Daltonics, US). TOF-MS showed the compound had the same molecular mass and the same number of chlorine atoms as des-ethyl-chlorfenvinphos. The UV/vis spectrum of this compound was the same as that of chlorfenvinphos, with a slight shift of 2 nm in the maximum wavelength. The peak at 4.1 min. with the same UV/vis spectrum as chlorfenvinphos is assumed to be the primary metabolite of chlorphenvinphos , des-ethyl-chlorfenvinphos [1]. This compound was quantified using the calibration curve for chlorfenvinphos, since the UV/vis spectra were similar.

1.2 Calculation of absorption rate constant

Since the absorption rate constant was not available for many of the organophosphates considered in this study, an attempt was made to estimate this parameter using *in silico* methods, as described below.

Initially it was attempted to predict the k_a for each organophosphate using the method proposed [2], where the apparent permeability (P_{app}) and effective permeability (Peff) are predicted based on molecular descriptors and translated to an abosorption rate constant (k_a) using various quantitative structure property relationships (QSPRs). However, this method underpredicted the extent of absorption in rats, while overpredicting the absorption in humans. This is likely caused by effects of fasting state, formulation and bile acids. However, for rats, a measured Peff parameter was available for chlorpyrifos via the study of Cook and Shenoy [3]. Using this parameter, the k_a could be calculated according to the methods described by [2] for chlorpyrifos, which resulted in simulations closely fitting the *in vivo* data. Using the method of [2], this value was extrapolated to other organophosphate pesticides. This was done as follows:

- First, k_a values were calculated for each organophosphate using the method of Punt et al. [2]. The equations are shown below, while further details can be found in the referenced paper.
    - $\log P_{app}$ (cm/s) = -4.36 -0.01*TPSA
    - $\log P_{eff,human}$ (10^-4 cm/s) = 0.4926*$\log P_{app}$ (10^-6 cm/s) – 0.1454
    - $P_{eff,rat} = P_{eff,human}/11.04$
    - $k_a = P_{eff}$ * 2 (cm/s)/R (cm) * 3600 (s/h)
- Next, a ratio was calculated between the k_a (calculated using the equations above) of each organophosphate and that of chlorpyrifos. These ratios were used to extrapolate k_a calculated from the <u>measured</u> P_{eff} of chlorpyrifos to obtain P_{eff} values for the other organophosphates.
- Since no permeability data were available for humans, the rat P_{eff} of chlorpyrifos was recalculated to a human P_{eff} according to Punt et al. [2]. This value was used to calculate human k_a values for the different OPs in the same way as was done for rats.
- Finally, the fraction absorbed (fa) was derived using the calculated k_a and equation 8 of Yu & Amidon [4].
    - fa = 1 – (1 + (k_a / k_t))^-7
        - Where k_t is the small intestinal transit rate, and can be found in SI table S2.

Calculated k_a (/h) values using the described method:

	CPF	DZN	DMO	FNT	MPT	EPT	CVP	PFS
**Rat ka**	2.4	2.07	2.22	1.65	1.65	1.7	2.29	2.22
**Rat fa**	0.94	0.92	0.93	0.88	0.88	0.88	0.94	0.95
**Human ka**	1.01	0.73	0.78	0.58	0.58	0.6	0.81	0.9
**Human fa**	0.94	0.88	0.89	0.82	0.82	0.83	0.90	0.92

This method resulted in good fits for most organophosphates in rats (see supporting figures), but less so for humans. This could be explained by the fact that rats were often exposed in the same way during different studies (using the same formulation), while human exposures were often different in

formulation and fasting state between different studies. This was evident from the difference between the reported fa for chlorpyrifos in the studies of Brzak et al. via Timchalk et al. (0.22) [5] and Nolan et al. (0.7) [6]. Therefore, the k_a for humans was fit to the excretion profile of urinary metabolites and k_a values of 0.1 and 0.6 were found for these studies, respectively. Using these k_a values, fa values were slightly overpredicted compared to the *in vivo* observed fa: 0.82 vs 0.7 for the Nolan et al. study and 0.28 vs 0.22 for all other chlorpyrifos studies in humans.

1.3 Calculation of physicochemical parameters for urinary metabolites

For urinary metabolites, conjugated and unconjugated derivatives were modelled together. This was done by taking the weighted average of the following physicochemical parameters: fup, BPR and all tissue partition coefficients. The weights for calculating the weighted average were based on the percentage of each conjugate or derivative found in urine (see table S4). An example of the calculations for calculating the weighted average fup of the derivatives of TCP, the urinary metabolite of CPF, is shown below.

	**Fraction found in urine**	**Rescaled fraction**	**Fu_p**	**Fractional Fu_p**
TCP	0.109	0.132	0.025	0.132 * 0.025 = 0.0033
TCP-glucuronide	0.69	0.835	0.378	0.835 * 0.378 = 0.3156
TCP-sulfate	0.027	0.033	0.533	0.033 * 0.533 = 0.0176
Other metabolites	0.037			
Sum	0.863*	1	-	0.336

** This is the fraction of the total administered dose found in urine. This needs to be rescaled to 100% to account only for the fractions found in urine. This is done, excluding 'other metabolites', which are either not identified or non-TCP derivatives.*

Following this example, a weighted average fup of 0.336 was used for TCP and its derivatives. The weighted average BPR and tissue partition coefficients were calculated in the same way. For the urinary metabolites MNP, PNP and BCP, active urinary excretion was shown to play a role in renal clearance. For those metabolites, a GFR multiplication factor was found for glucuronide and sulfate derivatives of phenolic compounds (1.6 and 5.5, respectively [7]), meaning that glucuronide and sulfate conjugates were excreted 1.6 and 5.5 times faster than the GFR, respectively. These multiplication factors were processed similarly to the physicochemical parameters above, resulting in a weighted average GFR multiplication factor estimating the effect of active excretion of these urinary metabolites.

1.4 Sensitivity/uncertainty matrix

The sensitivity/uncertainty matrix was used to evaluate the importance and uncertainty of each parameter. Sensitivity was put in one of three categories based on the sensitivity coefficient determined in the sensitivity analysis (see SI figures S20-S27): High (SC = 0.5 or higher), medium (SC = 0.2-0.5), low (SC = below 0.2) [8]. Results are shown in the table below.

		Uncertainty		
		**High**	**Medium**	**Low**
**Sensitivity**	**High**	k_a, V_{max}, K_m	fu_p	
	**Medium**			Liver related physiological parameters
	**Low**		BPR, $k_{tissue:plasma}$	Rest of physiological parameters

The absorption rate constant (k_a) is a highly sensitive parameter for all organophosphate pesticides tested (see SI 3.3 Sensitivity analysis). However this parameter is also highly uncertain, since for rats it is calculated from one permeability study using QSPRs that are not fully evaluated. Meanwhile the k_a value for humans was fit to urinary excretion of the urinary metabolites. The uncertainty of the k_a value could be lowered by performing Caco-2 permeability studies to derive P_{app} values, from which k_a and fa can be estimated (see 1.2 Calculation of absorption rate constant). However, these assays are challenging to perform accurately due to the lipophilicity of most of the considered compounds, since highly lipophilic compounds have the tendency to stick to plastic, resulting in low recovery [9]. The V_{max} and K_m were measured for each reaction of interest, at different substrate concentrations, and optimized for time and protein content. While metabolism in itself can be quite variable, due to the use of pooled subcellular fractions, the derived values could be considered to be reasonably certain, however, *in vitro* determined V_{max} and K_m values also suffer from high variation [10], possibly also due to the lipophilicity of the compounds in question. Next, the fup parameter is highly sensitive, but considered less uncertain compared to the ka. The fup was determined using the QSPR by Lobell and Sivarajah [11], which has been developed based on a wide range of compounds and was shown to adequately fit that dataset. Improving the certainty of these measurements could be done by using solid phase micro extraction (SPME) techniques for each individual compound, while using glass containers. Tissue:plasma partition coefficients (ktissue:plasma) and the BPR were both predicted using well established QSPRs [12–14]. Lastly, values for physiological parameters, including tissue volumes and blood flows, tissue composition, liver protein concentrations, glomerular filtration rate and intestinal transit times, are all considered to be certain and have been measured and used in multiple studies. Only a few physiological parameters were more sensitive, almost all of them being related to the liver compartment. This is expected, since this is where most of the metabolism takes place.

2. Supporting Tables

Table S1: Organophosphate pesticides and their rest groups

Description of the organophosphate molecular structure. In the main manuscript, the general structure can be found in figure 1. Here the restgroups are described for each pesticide considered in this study.

Pesticide	OTP or OPO	R_1	R_2	R_3
Chlorpyrifos	OTP	Ethyl	Ethyl	Aromatic
Diazinon	OTP	Ethyl	Ethyl	Aromatic
Profenofos	OPO	Ethyl	Propyl	Aromatic
Chlorfenvinphos	OPO	Ethyl	Ethyl	Aromatic
Ethyl-parathion	OTP	Ethyl	Ethyl	Aromatic
Methyl-parathion	OTP	Methyl	Methyl	Aromatic
Fenitrothion	OTP	Methyl	Methyl	Aromatic
Dimethoate	OTP	Methyl	Methyl	Aliphatic

OTP: organothiophosphate; OPO: organophosphate-oxon.

Table S2: Physiological parameters

PBK physiological input parameters in the PBK model of OPs for average unisex rats and humans. Note: for tissue volumes and blood flows in rats and humans, a tendency to male physiology and to Caucasian physiology, respectively, is expected due to due to these groups being most abundant in the data of [15].

Parameter	Description	Rat	Human	References
**Absorption parameters**				
GER	Gastric emptying rate (h^{-1})	2	2	
$K_{t\text{-}SI}$	Small intestine transit rate (h^{-1})	0.68 -> 4.77*	0.30 -> 2.11*	4,16
$K_{t\text{-}col}$	Colon transit rate (h^{-1})	0.26	0.09	
**Tissue parameters**				
BW	Body weight (kg)	0.25	70	
	Tissue volume fractions (L/kg BW)			
VFaf	Adipose tissue	0.07	0.214	
VLif	Liver	0.034	0.026	
VKif	Kidney	0.007	0.004	
VBrf	Brain	0.006	0.02	15
VBlf	Blood	0.074	0.079	
VSlf	Slowly perfused**	0.667	0.58	
VRif	Rapidly perfused***	0.087	0.063	
HC	Hematocrit	0.45	0.45	17
COf	Cardiac output (L/h/kg BW)	18.00	4.8	18
	Organ perfusion fractions (L/L CO)			
QFaf	Adipose tissue	0.07	0.052	
QLif	Liver	0.183	0.227	
QKif	Kidney	0.141	0.175	15
QBrf	Brain	0.02	0.114	
QSlf	Slowly perfused**	0.458	0.291	
QRif	Rapidly perfused***	0.128	0.141	
**Metabolism and excretion parameters**				
	Clearance scaling factors			19–23
MPL	mg microsomal protein/g of liver	32	35	
CPL	mg cytosolic protein/g of liver	80.7	87	
MPP	mg plasma protein/g of plasma	77	60	
GFRf	Glomerular filtration rate (L/h/kg BW)	0.312	0.108	18

* the SI transit rate was corrected according to [4] for the fact that SI content has to travel through multiple compartments. ** The slowly perfused compartment consists of bone, muscle and skin tissues. *** The rapidly perfused compartment consists of all other tissues not yet defined.

Table S3: Physico-chemical parameters for organophosphate pesticices

Chemical and physic-chemical parameters used in QSPRs to estimate chemical specific input parameters for each considered compound. Unless mentioned otherwise, logP values were taken from the referenced source or calculated as the average of VCClab (http://www.vcclab.org/lab/alogps/) [24], ChemAxon (https://chemicalize.com/) and molinspiration (https://www.molinspiration.com) predictions, pKa values were predicted using molGpKa (https://xundrug.cn/molgpka) [25], fu_p values were predicted using the QIVIVE tool (https://www.qivivetools.wur.nl/) [26] (QSPR: Lobell & Sivarajah [11]) and blood:plasma ratio (BPR) values were predicted using the simcyp BPR calculator [14].

**Compound**	**CAS#**	**Type**	**LogP ($LogK_{o/w}$)**	**pKa**	**fu_p**	**BPR**
Chlorpyrifos	2921-88-2	OTP	4.96[a]	NI	0.018	0.983
Chlorpyrifos-oxon	5598-15-2	OPO	3.89[b]	NI	0.051	0.692
TCP	6515-38-4	Urinary metabolite	3.21[c]	A: 4.55[c]	0.025	0.55[i]
TCP-glucuronide		Urinary metabolite	-0.190	A: 2.8	0.31	0.55[i]
TCP-sulphate		Urinary metabolite	-0.446	A: -4.5	0.359	0.55[i]
Diazinon	333-41-5	OTP	3.81[a]	B: 2.6[a]	0.11[h]	1.51
Diazinon-oxon	962-58-3	OPO	2.07[d]	B: 2.6*	0.262	0.786
IMHP	2814-20-2	Urinary metabolite	0.55[d]	A: 9.7[e]	0.623	0.55[i]
IMHP-OH		Urinary metabolite	-0.019	A: 9.3	0.623	0.55[i]
IMHP-glucuronide		Urinary metabolite	-1.540	A: 2.8, 8.5	0.583	0.55[i]
IMHP-sulphate		Urinary metabolite	-0.855	A: -3.8, 6.5	0.441	0.55[i]
Dimethoate	60-51-5	OTP	0.78	NI	0.573	1[j]
Omethoate	1113-02-6	OPO	-0.74	NI	0.866	1[j]
Fenitrothion	122-14-5	OTP	3.3[a]	NI	0.091	1.251
Fenitrothion-oxon	2255-17-6	OPO	1.69[d]	NI	0.344	1.046
MNP	2581-34-2	Urinary metabolite	2.48[d]	7.49[f]	0.103	0.55[i]
MNP-OH		Urinary metabolite	0.805	A: 6.8	0.222	0.55[i]
MNP-glucuronide		Urinary metabolite	-0.052	A: 2.8	0.286	0.55[i]
MNP-sulphate		Urinary metabolite	1.177	A: -4.6	0.125	0.55[i]

(continues on next page)

(Table S3 cont.)

Compound	CAS#	Type	LogP ($LogK_{o/w}$)	pKa	fu_p	BPR
Methyl-parathion	298-00-0	OTP	2.86	NI	0.136	1.2128
Methyl-paraoxon	950-35-6	OPO	1.25	NI	0.453	0.9634
PNP	100-02-7	Urinary metabolite	1.91	A: 7.0	0.109	0.55[i]
PNP-glucuronide		Urinary metabolite	-0.55	A: 2.8	0.378	0.55[i]
PNP-sulphate		Urinary metabolite	-0.66	A: -4.6	0.4	0.55[i]
Ethyl-parathion	56-38-2	OTP	3.83	NI	0.054	0.97
Ethyl-paraoxon	311-45-5	OPO	1.98	NI	0.28	1.0962
PNP (see methyl-parathion)						
Chlorfenvinphos	470-90-6	OPO	3.81	NI	0.043	0.992
Des-ethyl-chlorfenvinphos		Urinary metabolite	3.727	A: 1.2	0.016	0.55[i]
Profenofos	41198-08-7	OPO	4.68[a]	NI	0.023	1.295
BCP	3964-56-5	Urinary metabolite	3.34[d]	A: 7.92[g]	0.064	0.55[i]
BCP-glucuronide		Urinary metabolite	0.960	A: 2.7	0.146	0.55[i]
BCP-sulphate		Urinary metabolite	2.1	A: -5	0.062	0.55[i]

NI: Not ionizable; A: Acid; B: Base. Sources: a: PubChem, b: ChemAxon, c: [27], d: CompTox, e: [28], f: [29], g: [30], h: (H. X. Wu et al., 1996), i: 0.55 for acids according to Cubitt et al. [31]. * The pKa of diazinon-oxon is assumed to be the same as diazinon. j: when missing, a BPR of 1 was assumed for neutral compounds. OTP: organothiophosphate; OPO: organophosphate-oxon; TCP: 3,5,6-trichloropyridinol; IMHP: 2-isopropyl-4-methyl-6-hydroxypyrimidine; MNP: 3-methyl-4-nitrophenol; PNP: 4-nitrophenol; BCP: 4-bromo-2-chlorophenol.

Table S4: Metabolite ratios in urine

Relative amounts of derivatives and conjugates of the urinary metabolites found in the urine of rats from toxicokinetics studies. These ratios only function as an estimation of the ratio *in vivo*, since the actual ratio *in vivo* may differ per dose and per species, especially at high dose levels, due to possible saturation of enzyme and transporter kinetics. Additionally, on study qualitatively mentioned the extend of glucuronide conjugation in humans [32]. It was stated that after enzymatic hydrolysis, the amount of TCP in urine samples doubled. Therefore, glucuronidation of TCP in humans was assumed to be 50% of the TCP excreted in urine. For all other urinary metabolites in humans, ratios were not available. Therefore, rat data was used for those metabolites instead.

**Urinary metab-olites**	**Unconjugated**		**Conjugated**		**Other metabolites***	**% of dose excreted**	**Dose**	**References**
	Main metabolite	Derivatives (-OH)	Glucuronide	Sulphate				
TCP	10.9%		69%	2.7%**	3.7%	86.3% (83.9-88.7%)	0.5, 25 mg/kg bw	[33]
IMHP	22.5% (19-25%)	31%	21%***		0.5%	74.5% (69-80%)	1 mg per day for four days	[34]
MNP	11.4%	9.5%	5.5%	31.45%	40.5%	98%	15 mg/kg bw	[35]
BCP	1%		24.5%	42.5%	21%	89%	1 and 100 mg/kg	[36]
PNP	23.2%**		9%	62.25% (59.5-65%)	2.95%	97.4%	0.4-2 mg/kg bw	[37]

* Other metabolites consist of parent OP desalkyl derivatives or unidentified metabolites. **Rest amount was assumed based on missing percentage. *** Polar conjugates were assumed to be glucuronide and sulphate conjugates. TCP: 3,5,6-trichloropyridinol; IMHP: 2-isopropyl-4-methyl-6-hydroxypyrimidine; MNP: 3-methyl-4-nitrophenol; BCP: 4-bromo-2-chlorophenol; PNP: 4-nitrophenol.

Table S5: Metabolic parameters.

Michaelis-Menten parameters for the metabolism of organophosphates and their metabolites derived from incubations with liver microsomes and plasma used in the PBK model. For profenofos liver cytosolic metabolism is also included. Most values were taken from literature, while those for chlorfenvinphos were determined in this study.

**Tissue** / **Reaction**	**Liver**				**Plasma**				**References**
	Rat		Human		Rat		Human		
Chlorpyrifos	V_{max}*	K_m*	V_{max}*	K_m*	V_{max}*	K_m*	V_{max}*	K_m*	
CPF > CPO	0.268	10.6	0.156	28.59					[38–40]
CPF > TCP	1.14	4.08	0.234	4.326					
CPO > TCP	1280	345	37.98	627.9	400	250	23.84	290	
Diazinon									
DZN > DZO	0.187	59.6	0.288	4.745					[23]
DZN > IMHP	1.098	1.764	0.665	16.34					
DZO > IMHP	691.1	668	53.49	557.4	431.4	535.6	124	701	
Dimethoate									
DMO > OMO	0.341	20.6	0.132	339.2					[41]
DMO > DMCA	17.6	1012	0.4714	419.2					
OMO > HME									
Fenitrothion									
FNT > FNO	7.336	11.22	1.68	21.56					[42]
FNT > MNP	1.049	13.16	0.1825	4.084					
FNO > MNP	31.79	6589	3.089	12564	19.26	4075	2.142	5489	
Methyl-parathion									
MPT > MPO	7.336	11.22	1.68	21.56					Assumed the same as for fenitrothion, since these compounds only differ by one methyl group.
MPT > PNP	1.049	13.16	0.1825	4.084					
MPO > PNP	31.79	6589	3.089	12564	19.26	4075	2.142	5489	

(continues on next page)

(Table S4 cont.)

Tissue / Reaction	Liver				Plasma				References
	Rat		Human		Rat		Human		
Ethyl-parathion	V_{max}*	K_m*	V_{max}*	K_m*	V_{max}*	K_m*	V_{max}*	K_m*	
EPT > EPO	3.62	0.23	1.004	9					[43–47]
EPT > PNP	4.56	71.3	0.231	31.8					
EPT > PNP	19.03	181.7	1.35	280	126.17	617.8	3.5	240	
Chlorfenvinphos									
CVP > des-ethyl-CVP	0.46	5.9	0.565	6.36					This study.
Profenofos									
PFS > BCP microsomal	8.9	8.1	1.2	6.9					[48]
PFS > BCP cytosol/plasma	0.95	0.34	1.1	1.4	0.68	3.6	0.99	158	

*V_{max} in nmol/min/mg microsomal protein; K_m in µM. CPF: chlorpyrifos; CPO: chlorpyrifos-oxon; TCP: 3,5,6-trichloropyridinol; DZN: diazinon, DZO: diazinon-oxon; IMHP: 2-isopropyl-4-methyl-6-hydroxypyrimidine; DMO: dimethoate; OMO: omethoate; FNT: fenitrothion; FNO: fenitro-oxon; MNP: 3-methyl-4-nitrophenol; MPT: methyl-parathion; MPO: methyl-paraoxon; PNP: 4-nitrophenol; EPT: ethyl-parathion; EPO: ethyl-paraoxon; CVP: chlorfenvinphos; PFS: profenofos; BCP: 4-bromo-2-chlorophenol.

Table S6: Studies used for model evaluation

Overview of toxicokinetic studies used for evaluation of the PBK model predictions.

Compound	Exposure route	Formulation	Dose tested (mg/kg bw)	Species tested	Body weight (kg)	k_a used (/h)	Fasting state	Reference
Chlorpyrifos	Intravenous	Saline solution with 5% Tween	0.5, 1, 2.5, 5	Rat, SD, adult male	0.35	-	Not mentioned	[49]
Chlorpyrifos	Oral gavage	Corn oil	15, 60	Rat, SD, adult male	0.275	2.4	12h prior – 3 h after	[50]
Chlorpyrifos	Oral gavage	Corn oil	5, 10, 50, 100	Rat, F344, males (10-11 weeks)	0.210	2.4	16h prior	[5]
Chlorpyrifos	Intravenous	Saline solution with 5% Tween	4.05	Rat, SD, male	0.245	-	12h prior – 3h after	[51]
Chlorpyrifos	Oral gavage	Saline solution with 5% Tween	4.15	Rat, SD, male	0.245	2.4	12h prior – 3h after	[51]
Chlorpyrifos	Oral gavage	Corn oil	3.49	Rat, SD, male	0.245	2.4	12h prior – 3h after	[51]
Chlorpyrifos	Oral	Lactose tablet	0.5	Human, Caucasian, male (27-50 years)	83.3	0.6	No, meal right before dose	[6]
Chlorpyrifos	Oral	Gelatin capsule	0.5, 1, 2	Human	75	0.1	10h fast with snack prior	Brzak et al. 2000 via [52]
Chlorpyrifos	Oral	Capsule	0.5, 1, 2	Human, male, female	54.8*	0.1	Not mentioned	[5]
Diazinon	Oral	'Water diluted ethanol solution'	0.011	Human, male, female	83	Not modelled	Not mentioned	[53]
Diazinon	Oral	Soybean oil in gelatine capsule	0.12, 0.2, 0.21, 0.3	Human, male (18-48 years)	76.1, 78.6, 75.4, 71.2	0.1	After breakfast	Wong AJ & Anderson GD (2000) via [54]
Diazinon	Oral	Corn oil	15, 60	Rat, SD, adult male	0.275	2.07	12h prior – 3h after	[50]
Diazinon	Intravenous	Methanol in 20% IV fat emulsion	1, 10	Rat, SD, male	0.375	-	Not mentioned	[55]
Diazinon	Intravenous	Saline solution with 5% Tween	15	Rat, Wistar, male	0.278	-	16h prior	[56]
Dimethoate	Intravenous	5% ethanol, 10% captisol water solution	3	Rat, SD, adult male	0.375	-	Fasted overnight	[41,57]
Dimethoate	Oral	Corn oil	4	Rat, SD, adult male	0.375	2.22	Fasted overnight	[41,57]

(continues on next page)

(Table S6 cont.)

Compound	Exposure route	Formulation	Dose tested (mg/kg bw)	Species tested	Body weight (kg)	k_a used (/h)	Fasting state	Reference
Fenitrothion	Oral	Capsule	0.36	Human, male, female (23-50 years)	75	0.1	Overnight fast, with food	[58]
Fenitrothion	Oral	Olive oil in capsules	Total amounts: 5, 10, 15, 20mg	Human	77.9, 71.6, 80, 66.4	0.1	Not mentioned	[59]
Fenitrothion	Intravenous	'emulsifyable concentrate'	3, 15	Rat, male	0.14	-	Not mentioned	[60]
Fenitrothion	Oral	Corn oil	25, 50, 100	Rat, SD, male	0.29	1.65	Not mentioned	[61]
Fenitrothion	Oral gavage	'Edible oil'	47	Rat, albino, female	0.19	1.65	Not mentioned	[62]
Fenitrothion	Intravenous	Not mentioned	0.94	Rat, albino, female	0.19	-	Not mentioned	[62]
Methyl-parathion	Intravenous	'emulsifyable concentrate'	3, 15	Rat, male	0.14	-	Not mentioned	[60]
Methyl-parathion	Intravenous	Propylene glycol	1.5	Rat, SD, female	0.2	-	24h prior	[63]
Methyl-parathion	Oral	Propylene glycol	1.5	Rat, SD, female	0.2	1.65	24h prior	[63]
Ethyl-parathion	Intravenous	Propylene glycol	3	Rat, Wistar, male	0.25	-	Not mentioned	[64]
Ethyl-parathion	Intravenous	PEG400 solution	3	Rat, SD, male	0.305	-	Access to food, water	[65]
Ethyl-parathion	Oral	Corn oil	10, 12.5, 15	Rat, SD, male	0.29	1.7	Not mentioned	[61]
Chlorfenvinphos	Oral	Olive oil	25-30	Rat, Porton, young adult	0.175	2.29	Not mentioned	[66]
Chlorfenvinphos	Oral	Olive oil	30	Rat, F334	0.25	2.29	Not mentioned	[67]
Chlorfenvinphos	Oral	Olive oil	30	Rat, F334	0.25	2.29	Not mentioned	[68]
Chlorfenvinphos	Intravenous	1% Tween 80 sol.	5	Rat, F334	0.25	-	Not mentioned	[68]
Profenofos	Oral	Different language	35.8	Rat, SD	0.175	2.22	Different language	[69]

*Not reported by study; average bodyweight was calculated based on reported dose and fraction absorbed. SD: Sprague Dawley; k_a: absorption rate constant. Possible corrections of the *in vivo* data are discussed in the materials and methods.

Table S7: Simulated and observed toxicokinetic parameters for organophosphate exposures

For further comparison, toxicokinetic parameters were determined to better quantify the extend of over/under-prediction for model evaluation. These parameters were determined using GraphPad Prism (see materials and methods in main manuscript). Only acute, single dose exposure studies that report T_{max}, C_{max} or AUC values are included in this table. AUC was taken for the time period mentioned in the respective study. If no observation time was mentioned, AUC was calculated until 100 hours.

	Study	Compound	Medium	Dose (mg/kg bw)	Route	Tmax (h)			Cmax (uM)			AUC (uM · h)		
						Predicted	Observed	FD	Predicted	Observed	FD	Predicted	Observed	FD
Rat	49	CPF	Bl	0.5	IV	0	-		19.27	-		1.358	1.353**	1.00
		TCP				4.7	-		2.368	-		55	44.11	1.25
		CPF		1	IV	0	-		38.54	-		2.718	3.090**	1.14
		TCP				4.7	-		4.733	-		110.0	187.7	1.71
		CPF		2.5	IV	0	-		96.36	-		6.806	7.045**	1.04
		TCP				4.7	-		11.81	-		275.0	631.9	2.30
		CPF		5	IV	0	-		192.7	-		13.65	9.978**	1.37
		TCP				4.7	-		23.54	-		549.9	1082	1.97
	51	CPF	Bl	4.05	IV									
		TCP				4.7	8.0	1.70	2.1	2.5	1.19	49	68.69	1.40
		CPF		4.15	Oral									
		TCP				2.1	0.5	4.17	4.082	20.24	4.95	59.97	94.91	1.58
		CPF		3.49	Oral									
		TCP				2.1	6.0	2.85	3.436	11.33	3.30	50.43	81.85	1.62
	50	CPF	Bl	15	Oral	0.6	<3	<5	0.6949	0.2853	2.43	2.302	2.852	1.23
		TCP				2.2	6	2.73	14.47	9.6256	1.49	182.0	131.02	1.39
		CPF		60	Oral	0.6	-		8.153	3.2516	2.5	23.84	13.69	1.75
		TCP				2.8	-		48.85	23.787	2.04	691.1	282.22	2.44
	5	CPF	Bl	5	Oral	0.6	-		0.1803	-		0.6854	0.4	1.72
		CPF		10	Oral	0.6	-		0.4067	-		1.502	1.1	1.37
		CPF		50	Oral	0.6	-		5.796	-		18.02	5	3.57
		CPF		100	Oral	0.7	-		19.37	-		66.70	12.5	5.26
Hu	6*	CPF	Bl	0.5	Oral		-			-			-	
		TCP				6.8	6	1.136	1.461	4.687	3.19	94.42	231.82	2.45

(continues on next page)

(Table S7 cont.)

	Study	Compound	Medium	Dose (mg/kg bw)	Route	Tmax (h)			Cmax (µM)			AUC (µM · h)		
						Predicted	Observed	FD	Predicted	Observed	FD	Predicted	Observed	FD
Rat	55	DZN	Pl	1	IV	0	-		22.31	-		1.515	2.097***	1.38
		DZN		10	IV	0	-		223.1	-		15.51	22.30***	1.44
	50	DZN	Bl	15	Oral	0.6	6	10	0.6368	0.1314	4.76	1.426	1.6100	1.13
		IHMP				1.6	6	3.75	15.12	9.2962	1.63	99.81	98.561	1.01
		DZN		60	Oral	0.7	-		16.39	0.5914	25	32.98	6.2428	5.26
		IMHP				2.2	-		50.82	61.305	1.21	392.7	525.66	1.34
	41	DMO	Pl	3	IV	0	-		176.80	-		16.42	10.13***	1.62
		OMO				0.8	0.5	1.6	4.12	1.28	3.22	9.27	3.345	2.69
		DMO		6	Oral	0.8	0.24	3.33	7.37	5.65	1.3	10.81	6.633	1.63
		OMO				1.6	0.48	3.33	7.71	2.41	3.20	22.77	11.45	1.99
	63	MPT	Pl	1.5	IV	0	-		77.01	-		4.283	3.298***	1.30
		MPT		1.5	Oral	0.7	0.27	2.56	0.048274	0.118	2.5	0.086	0.5475	6.25
	64	EPT	Pl	3	IV	0	-		143.50	-		9.518	11.68***	1.22
		EPO				0.1	0.121	1.21	0.21	0.67	3.23	0.1041	0.2401	2.33
	65	EPT	Pl	3	IV	0	-		143.50	-		9.518	4.094***	2.32
	68	CVP	Pl	5	IV	0	-		187.90	-		12.77	8.917***	1.43
		CVP		30	Oral	0.9	1	1.11	0.469	0.158	2.97	1.333	0.542	2.46
	67	CVP	Pl	30	Oral	0.9	1	1.11	0.469	0.493	1.05	1.333	0.92	1.45
Hu	58	FNT**	Pl	0.18	Oral	2.3	2.5	1.09	0.003468	0.0079	2.28	0.02294	0.0069	3.33

* Other studies reported significant differences when they compared to the study from Nolan et al. 1984. ** Reported AUCs were very high, AUCs were calculated based on datapoints and C0 of predicted was used for C0 in observed data. ** Reported toxicokinetic values do not reflect same data in accompanying graph in paper. *** IV AUCs were recalculated based on *in vivo* observed concentration-time data points and C0 of predicted was used for C0 in observed data, since datapoints were missing in the first few minutes, causing large differences in AUC due to the way it is calculated. Dashes (-) represent *in vivo* parameters that were not reported. FD: Fold difference. CPF: chlorpyrifos; TCP: 3,5,6-trichloropyridinol, DZN: diazinon, IMHP: 2-isopropyl-4-methyl-6-hydroxypyrimidine; DMO: dimethoate; OMO: omethoate; FNT: fenitrothion; MPT: methyl-parathion; EPT: ethyl-parathion; CVP: chlorfenvinphos; Bl: blood; Pl: plasma.

Table S8: Species differences between rats and humans

The C_{max} values of simulation with 1 mg/kg bw exposures of each OP pesticide was compared between rats and humans.

**Pesticide**	**C_{max} rat (µM)**	**C_{max} human (µM)**	**Fold difference**
Chlorpyrifos	0.0295	0.0281	1.05
Chlorpyrifos-oxon	0.000077	0.00134	17.4
Diazinon	0.0228	0.0529	2.32
Diazinon-oxon	0.000287	0.00030	1.05
Ethyl-parathion	0.000845	0.0200	23.7
Ethyl-paraoxon	0.067	0.147	2.19
Methyl-parathion	0.031	0.095	3.06
Methyl-paraoxon	1.53	1.623	1.06
Fenitrothion	0.0249	0.0242	1.03
Fenitro-oxon	1.47	0.45	3.27
Dimethoate	0.98	1.08	1.10
Omethoate	1.08	0.15	7.20
Profenofos	0.0013	0.00097	1.34
Chlorfenvinphos	0.142	0.023	6.17

3. Supporting Figures

3.1 Chlorfenvinphos metabolism kinetics

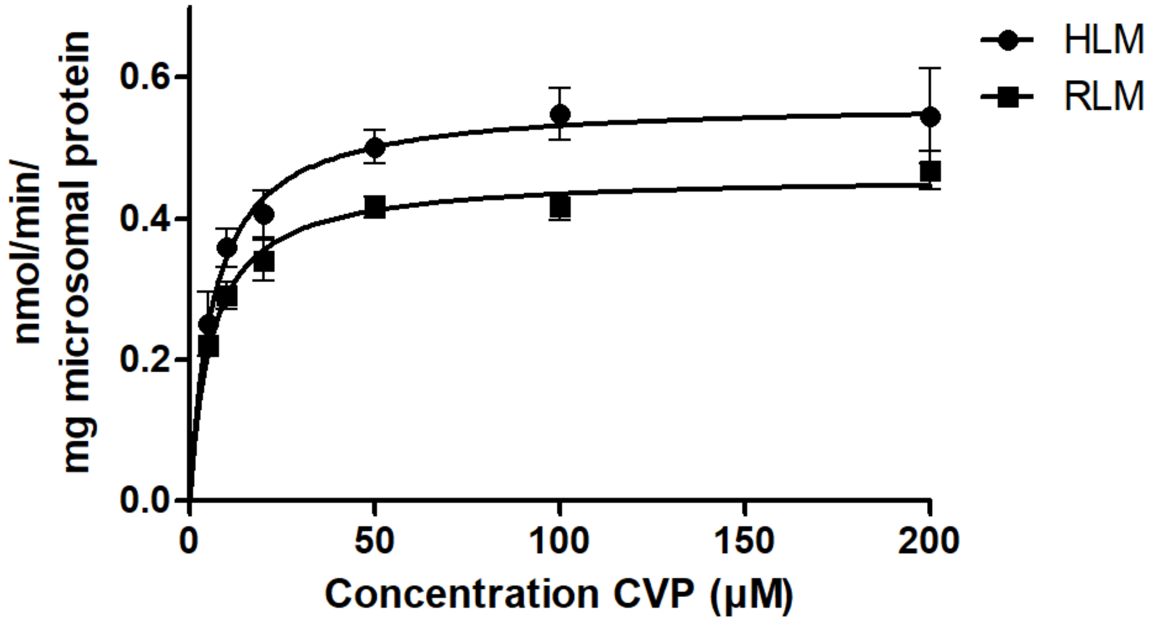

Figure S1: Concentration dependent metabolic reaction rates for the conversion of chlorfenvinphos by CYP P450 dependent des-ethylation to des-ethyl-chlorfenvinphos, in incubations with pooled rat (squares) or human (dots) liver microsomes. Data are plotted as the mean of three experiments with their respective SEM.

3.2 Simulated and measured concentration-time profiles

3.2.1 Chlorpyrifos

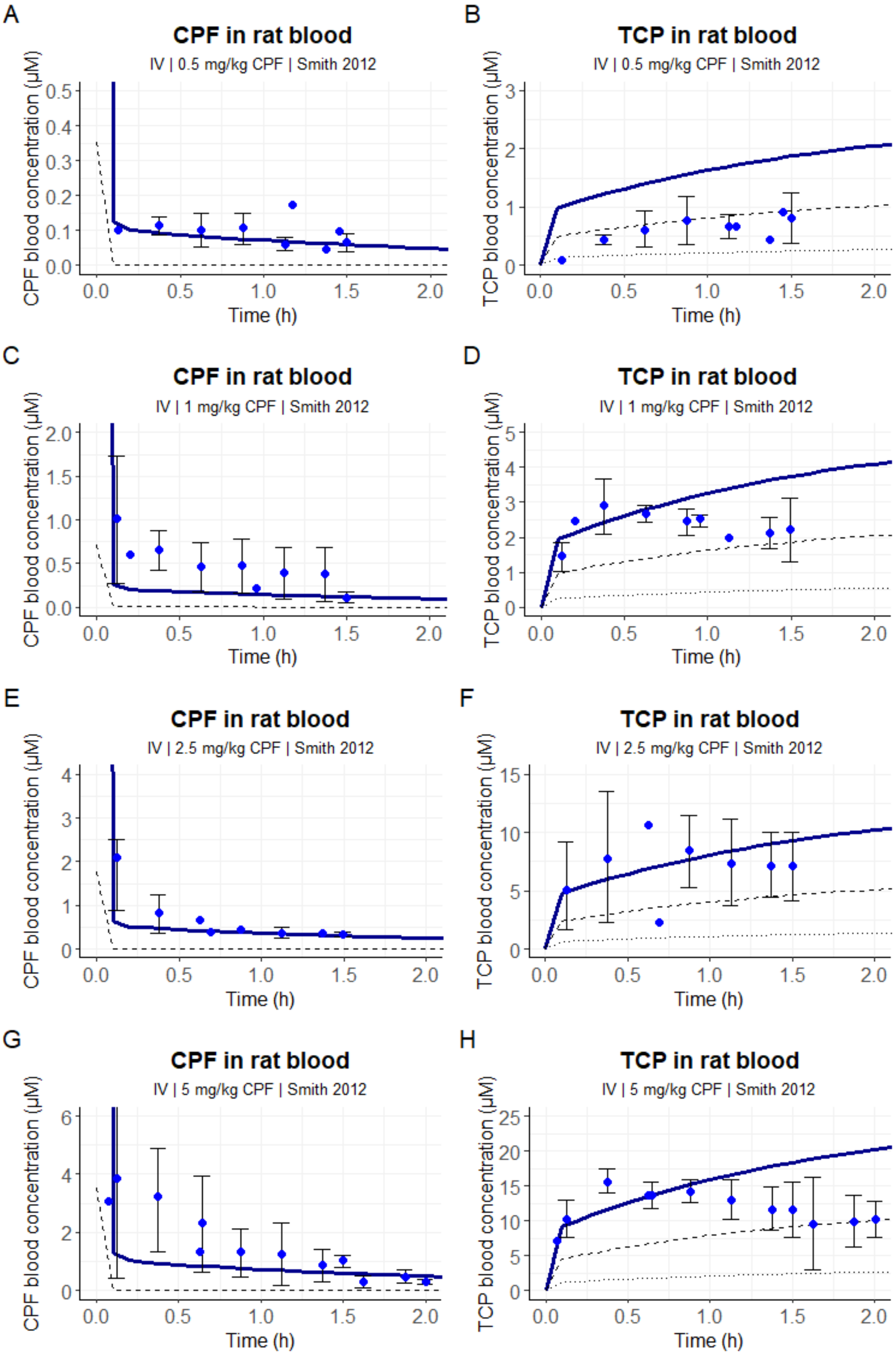

Figure S2: Simulated (solid line) and *in vivo* (filled circles) chlorpyrifos (A, C, E, G) and TCP (B, D, F, H) blood concentration-time profiles after rats were intravenously exposed to 0.5, 1, 2.5 and 5 mg/kg of chlorpyrifos. Solid, dotted and dashed lines represent simulations of total, unconjugated and unbound concentration in blood, respectively. *In vivo* data was taken from [49], where total blood concentrations were measured. CPF: chlorpyrifos; TCP: 3,5,6-trichloropyridinol.

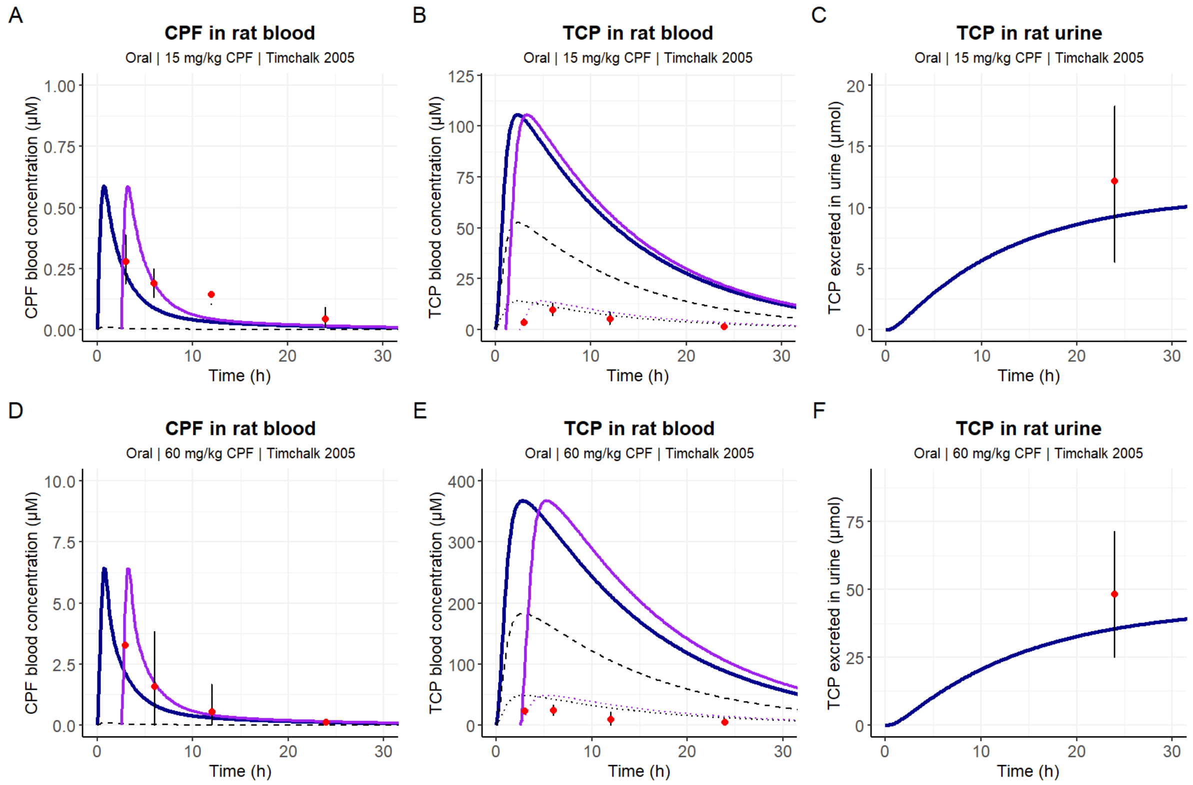

Figure S3: Simulated (solid and dashed lines) and observed (shaped markers) chlorpyrifos (A, D) and TCP (B, E) concentration-time and cumulative urinary excretion-time profiles (C, F) after oral exposure of rats to chlorpyrifos at two different doses: 15 and 60 mg/kg bw. Solid, dotted and dashed lines represent total, unconjugated and unbound concentrations in blood, respectively. Purple lines show model predictions with a 2.5 hour delay in absorption. *In vivo* data were taken form [50], where for chlorpyrifos total concentrations were measured, while for TCP in blood and urine the unconjugated and total concentrations were measured respectively. CPF: chlorpyrifos; TCP: 3,5,6-trichloropyridinol.

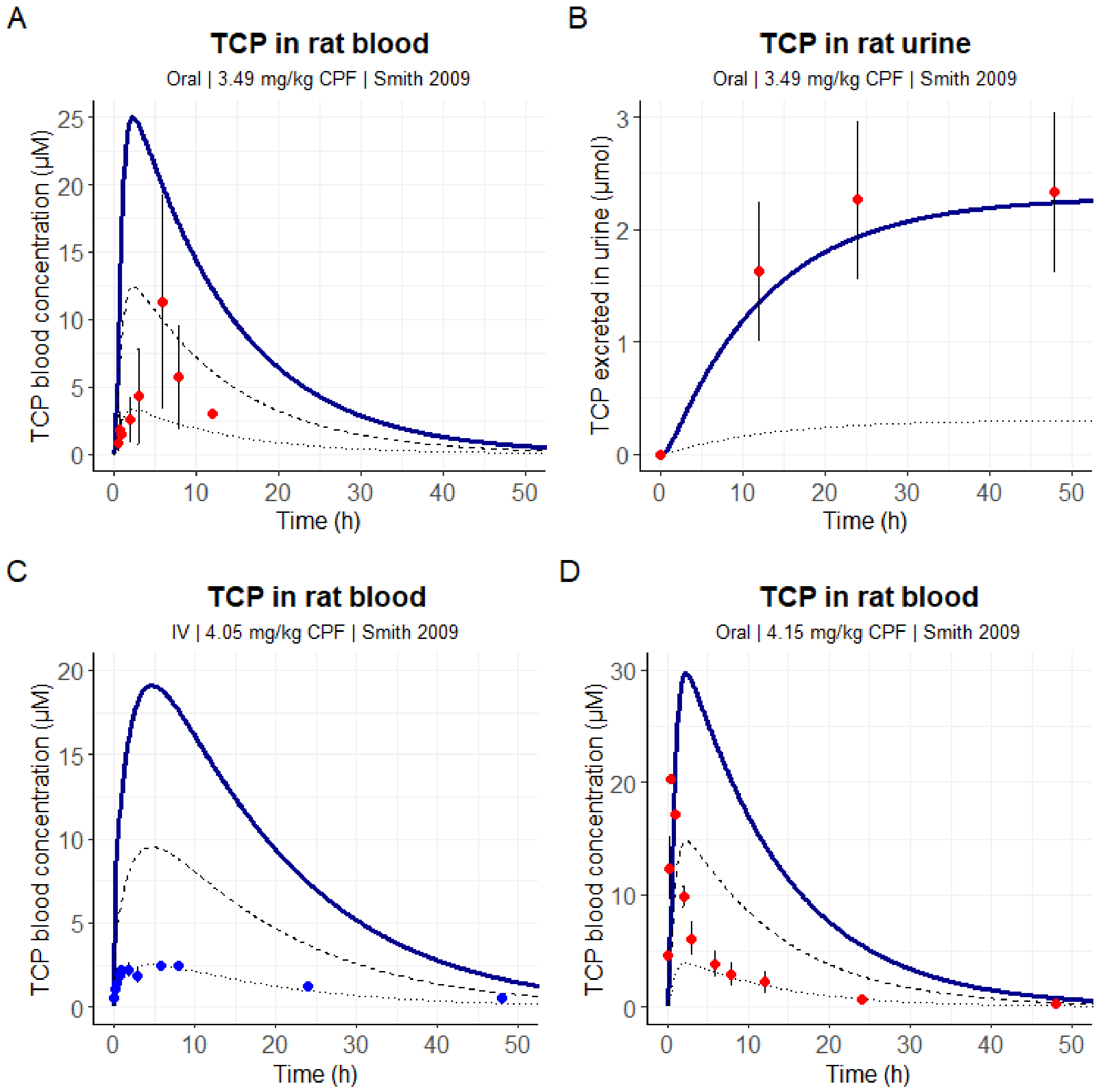

Figure S4: Simulated (solid and dashed lines) and observed (circles) TCP concentration-time (A, C, D) and cumulative urinary excretion-time (B) profiles after IV (C) and oral (A, B, D) exposure of rats to chlorpyrifos at two different doses: 4.05, 4.15, 3.49 mg/kg. Solid, dotted and dashed lines represent simulations of total, unconjugated and unbound TCP in blood, respectively. Panel D shows oral exposure to chlorpyrifos in a watery 5% tween solution, while in panel A and B chlorpyrifos was administered in a corn oil formulation. *In vivo* data were taken from [51] where unconjugated TCP was measured. CPF: Chlorpyrifos; TCP: 3,5,6-trichloropyridinol.

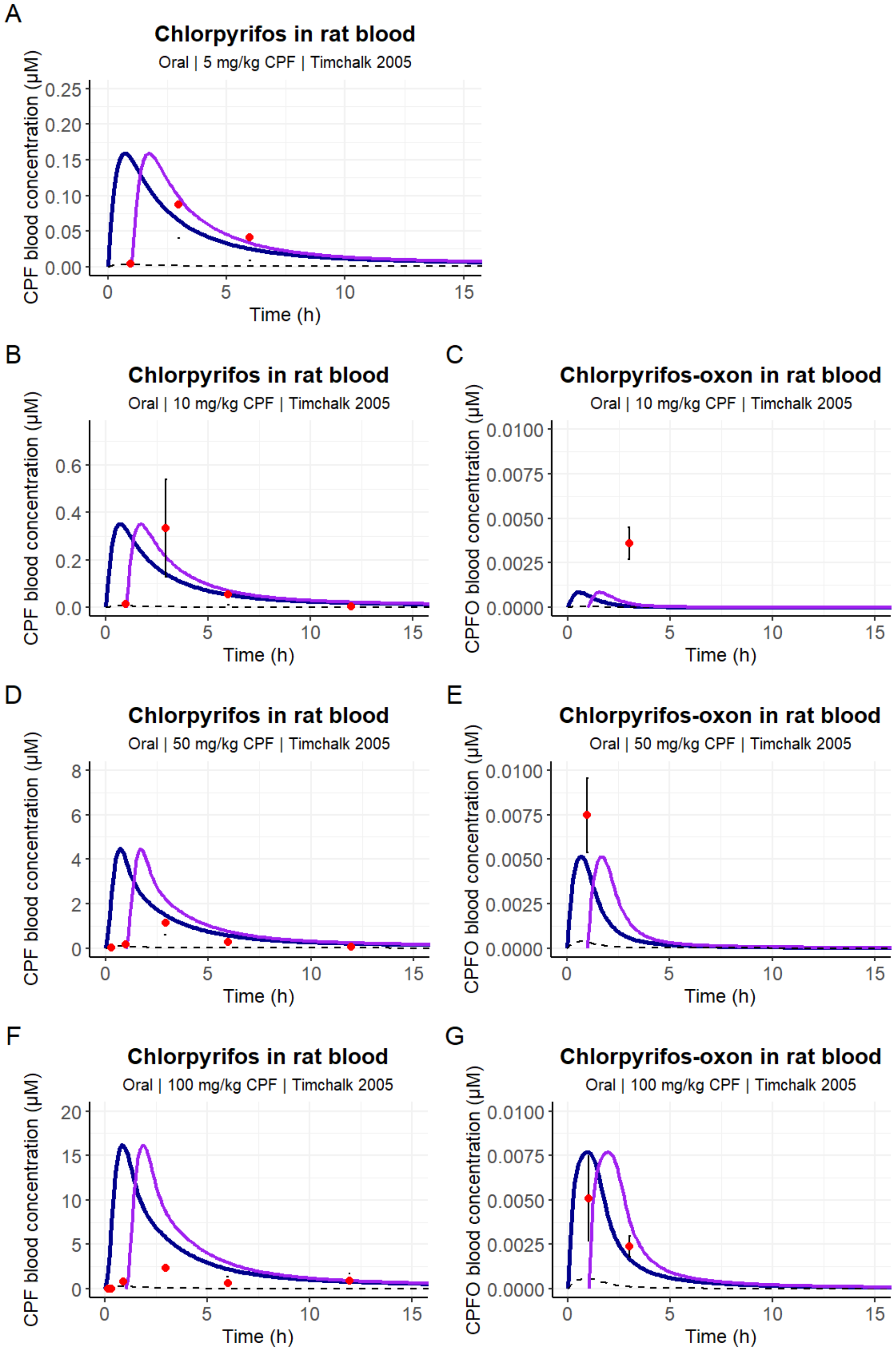

Figure S5: Simulated (solid lines) and observed (circles) chlorpyrifos (A, B, D, F) and chlorpyrifos-oxon (C, E, G) concentrations after oral exposure of rats to chlorpyrifos at doses of 5, 10, 50 and 100 mg/kg. Solid and dashed lines represent simulations of total and unbound concentrations in blood, respectively. Purple lines show model predictions with a 1 hour delay in absorption. *In vivo* data were taken from [5], where total concentrations were measured. CPF: Chlorpyrifos; CPO: Chlorpyrifos-oxon.

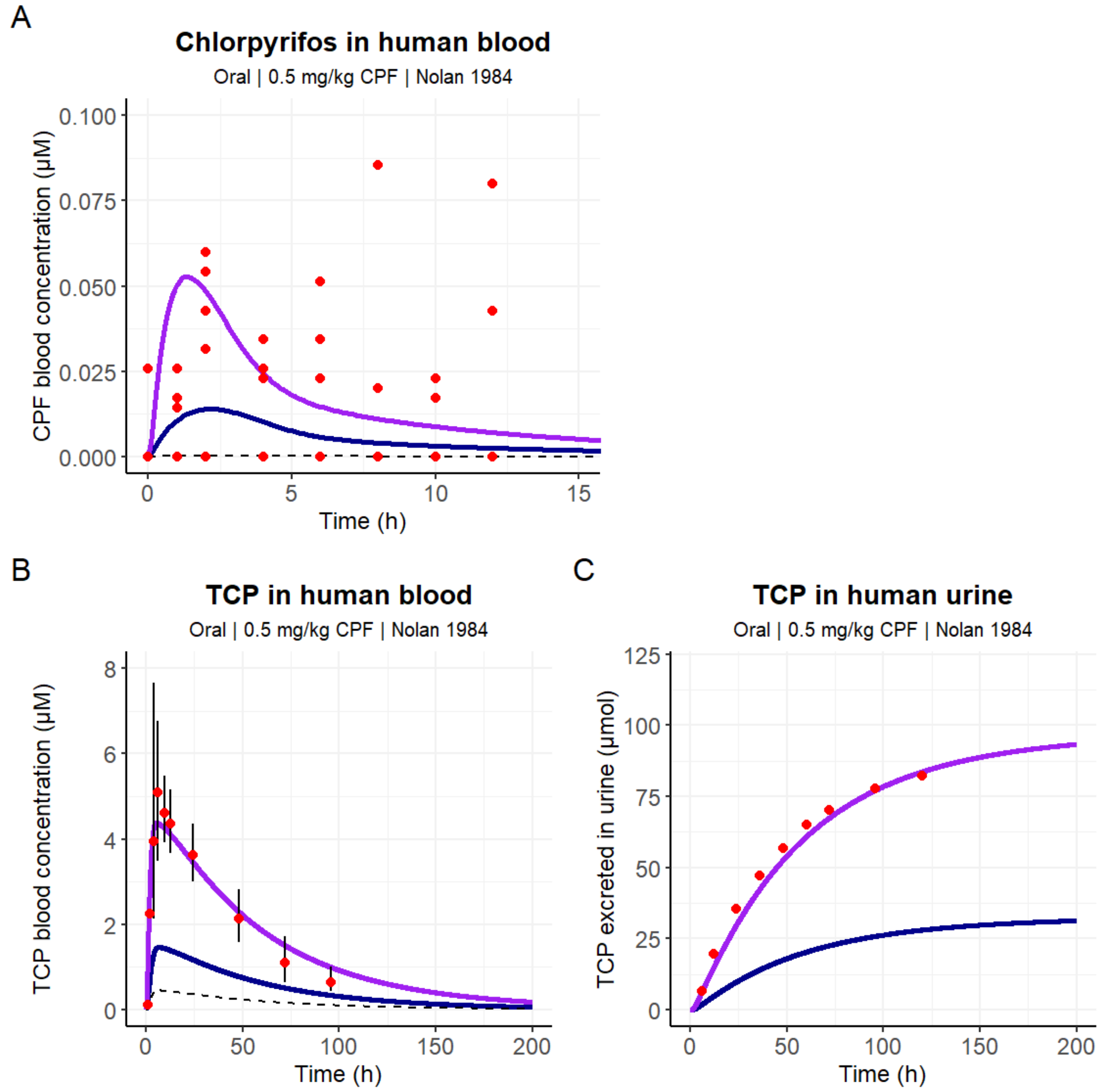

Figure S6: Simulated (solid lines) and observed (circles) chlorpyrifos (A) and TCP blood (B) concentrations and cumulative urinary excretion (C) after oral exposure of human volunteers to 0.5 mg/kg bodyweight chlorpyrifos. Solid and dashed lines represent simulations of total and unbound concentrations in blood, respectively. Blue lines represent simulations with a generalized k_a of 0.1, purple lines represent simulations with the k_a 0.6, as reported by the study. *In vivo* data was taken from [6] and plotted as red dots representing individual measurements (A) or mean of several volunteers (B and C) of total blood or urine concentration of CPF and TCP. In banel B, the SD is also shown. CPF: Chlorpyrifos; TCP: 3,5,6-trichloropyridinol.

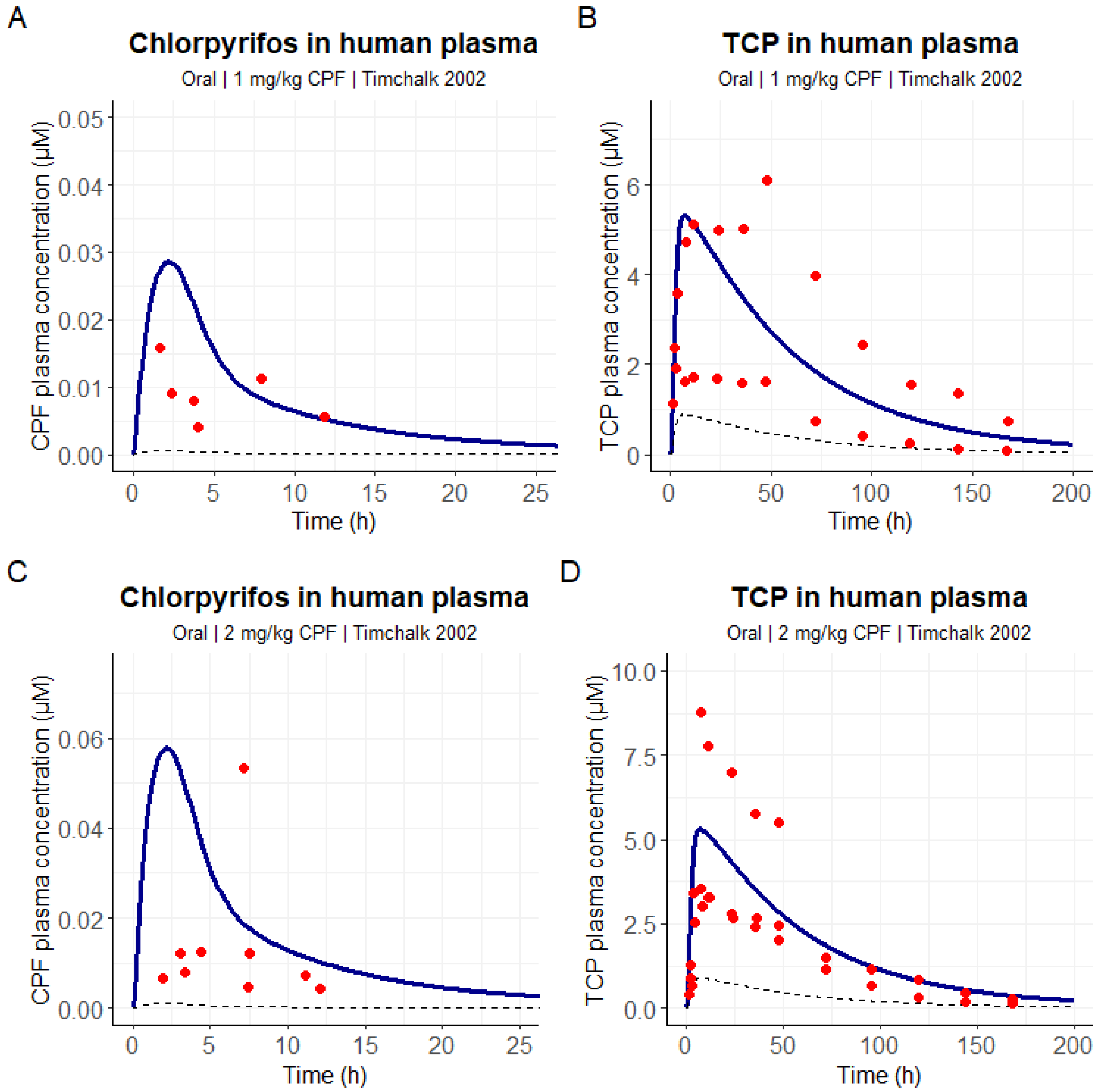

Figure S7: Simulated (solid line) and observed (shaped markers) chlorpyrifos (A, C) and TCP (B, D) plasma concentration-time profiles after oral exposure of human volunteers to chlorpyrifos at two different doses: 1 mg/kg (A, B), and 2 mg/kg (C, D). Solid and dashed lines represent simulations of total and unbound concentrations in plasma, respectively. *In vivo* data was taken from [5] and reported as individual measurements for different volunteers of total plasma concentrations of CPF and TCP. Data reported for individuals are separately denoted, while simulations use an average BW. CPF: chlorpyrifos; TCP: 3,5,6-trichloropyridinol.

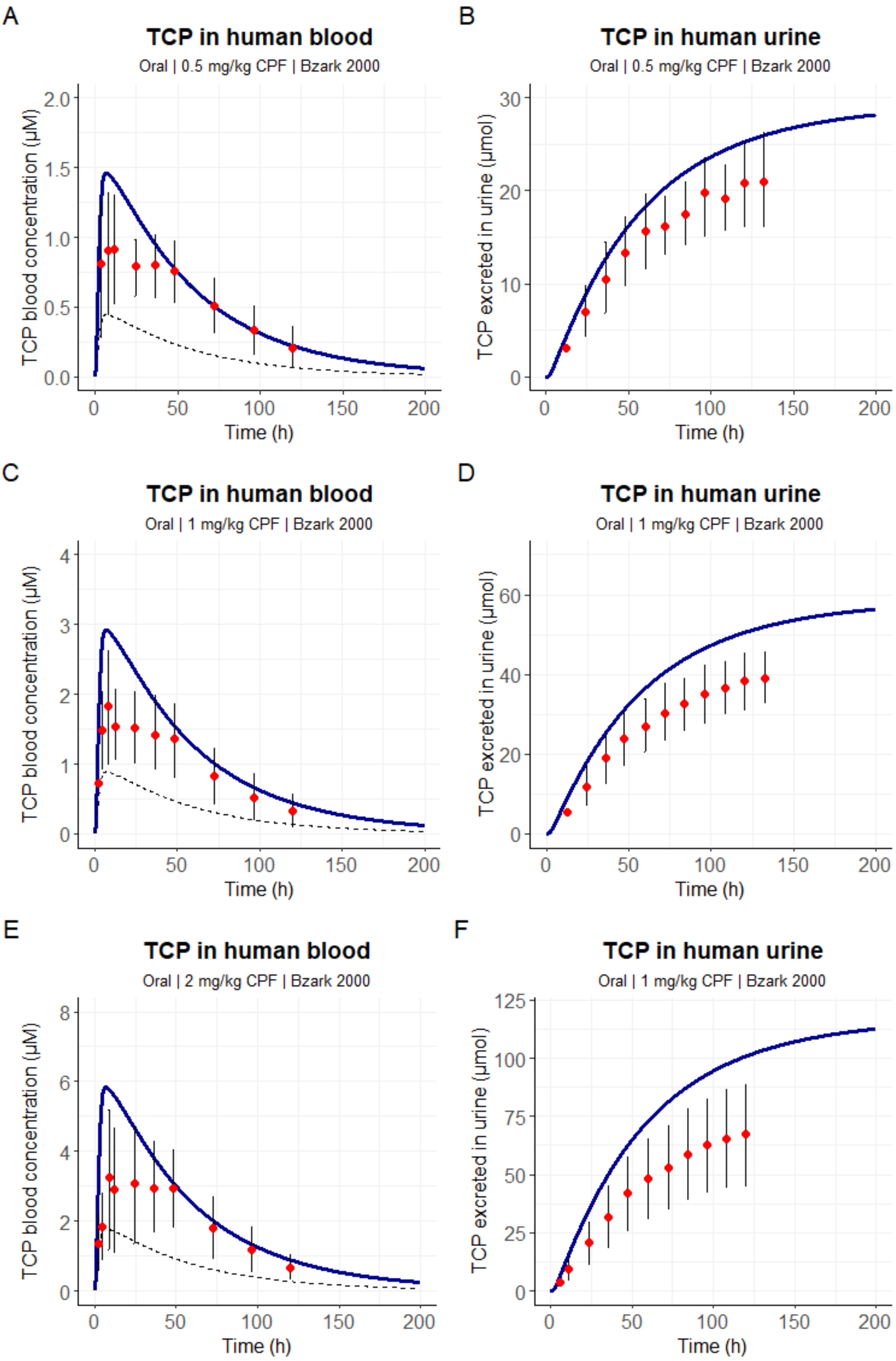

Figure S8: Simulated (solid lines) and observed (circles) TCP blood concentrations (A, C, E) and cumulative urinary excretion (B, D, F) after human volunteers were exposed to 0.5, 1 or 2 mg/kg bodyweight of chlorpyrifos. Solid and dashed lines represent simulations of total and unbound concentrations in blood, respectively. *In vivo* data were taken from Brzak et al. 2000 via [52], where total TCP blood and urine concentrations were measured and plotted as mean ± SD. CPF: Chlorpyrifos; TCP: 3,5,6-trichloropyridinol.

3.2.2 Diazinon

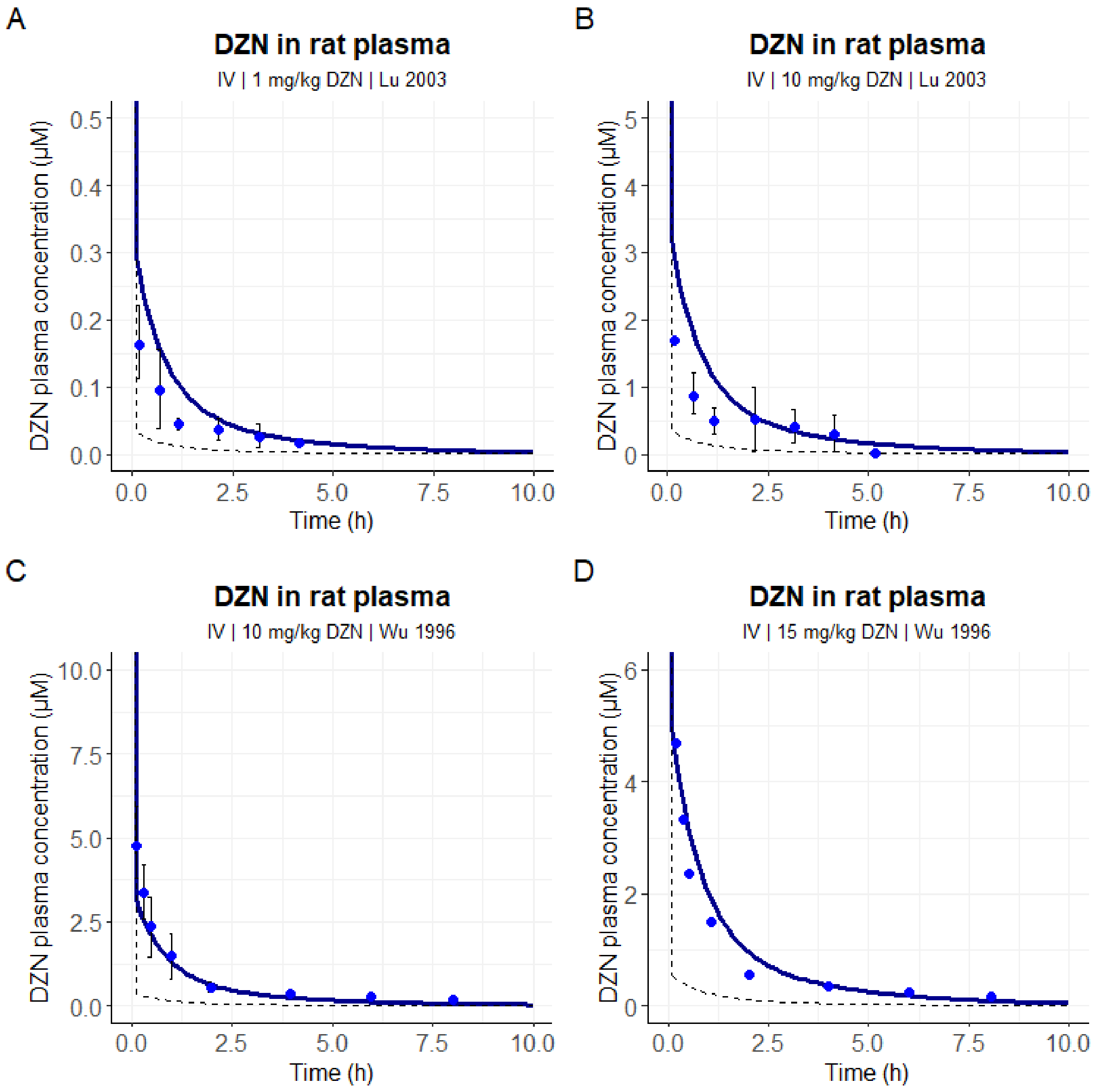

Figure S9: Simulated (solid lines) and observed (circles) diazinon plasma concentrations after rats were intravenously exposed to 1 (A), 10 (B, C) or 15 mg/kg diazinon. Solid and dashed lines represent simulations of total and unbound concentrations in plasma, respectively. *In vivo* data were taken [55,56], where total plasma concentrations were measured. DZN: Diazinon.

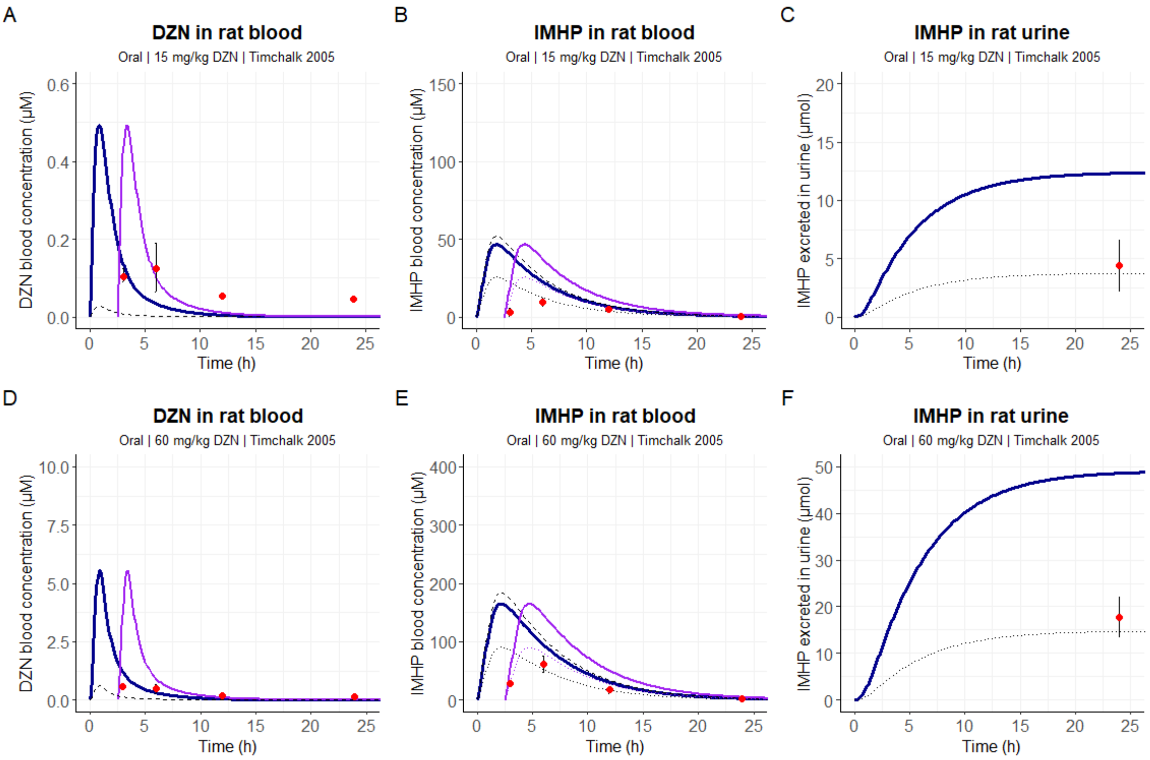

Figure S10: Simulated (solid and dashed lines) and observed (circles) diazinon (A, D) and IMHP (B, E) concentration-time and cumulative urinary excretion-time (C, F) profiles after oral exposure of rats to diazinon at two different doses: 15 and 60 mg/kg. Solid, dotted and dashed lines represent total, unconjugated and unbound concentrations in blood, respectively. Purple lines show model predictions with a 2.5 hour delay in absorption. *In vivo* data was taken form [50], where unconjugated IMHP was measured. DZN: Diazinon, IMHP: 2-isopropyl-4-methyl-6-hydroxypyrimidine.

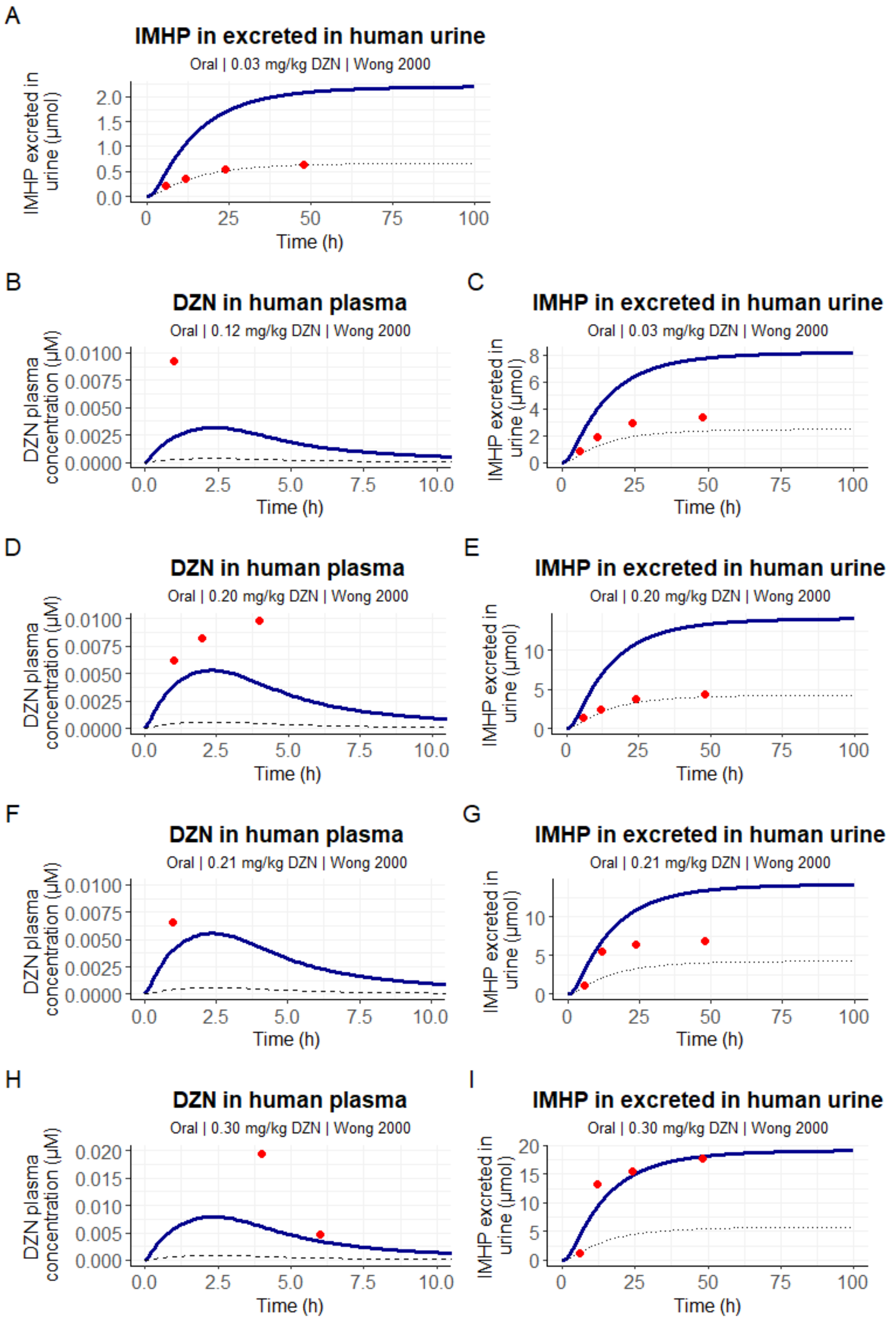

Figure S11: Simulated (solid and dashed lines) and observed (circles) diazinon plasma concentration-time profile (A, C, E, G) and cumulative IMHP urinary excretion-time profile (B, D, F, H) after human volunteers were exposed to 0.03, 0.12, 0.2, 0.21 or 0.3 mg/kg bodyweight of diazinon. Solid, dotted and dashed lines represent total, unconjugated and unbound concentrations in blood, respectively *In vivo* data were taken from Wong et al. 2000 via [54]. No further information was available about this study. It was assumed that unconjugated IMHP was measured, since IMHP in itself breaks down in acidic environments. DZN: Diazinon, IMHP: 2-isopropyl-4-methyl-6-hydroxypyrimidine.

3.2.3 Fenitrothion

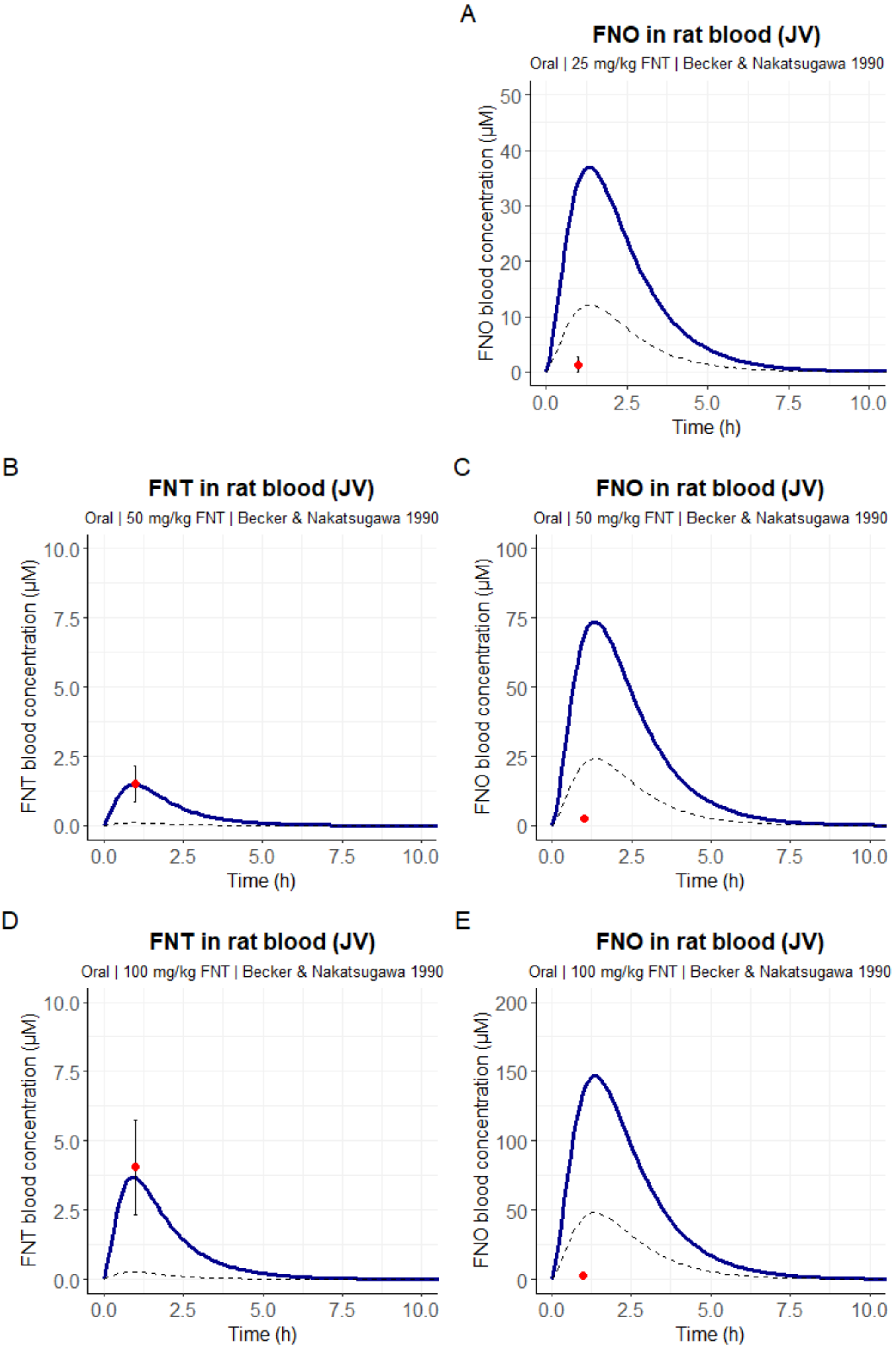

Figure S12: Simulated (solid and dashed lines) and observed (circles) fenitrothion (B, C) and fenitro-oxon blood (jugular vein) concentration-time profile (A, C, E) in rats exposed to 25, 50 and 100 mg/kg bodyweight of fenitrothion. Solid and dashed lines represent total and unbound concentrations in blood, respectively *In vivo* data were taken from [61], where total concentrations were measured. JV: jugular vein; FNT: fenitrothion; FNO: fenitro-oxon.

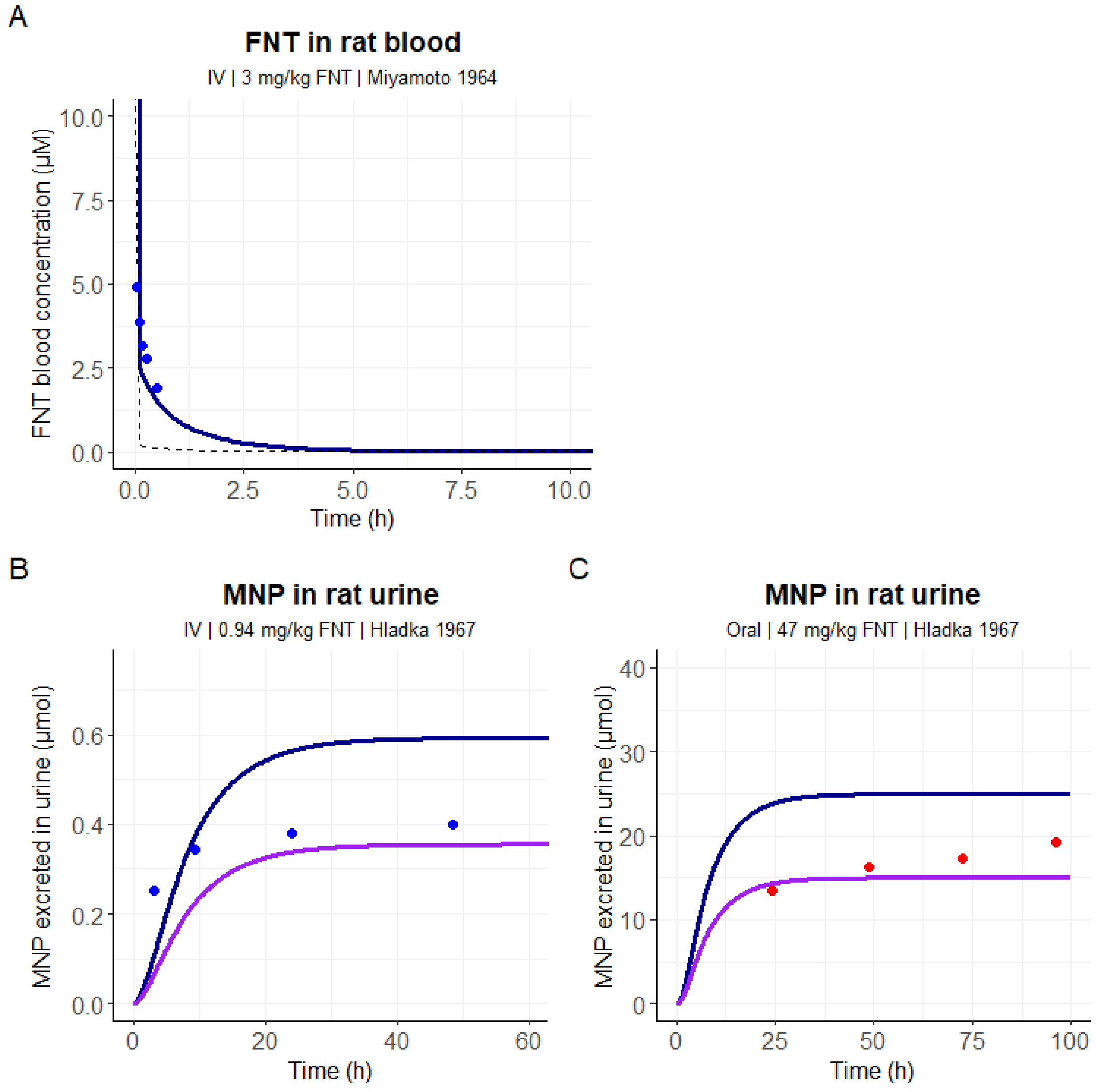

Figure S13: Simulated (lines) and observed (circles) fenitrothion in blood (A) and urinary excretion of MNP after IV exposure of rats to 0.94 mg/kg bw (B) and oral exposure of rats to 47 mg/kg bw fenitrothion (C). Blue and purple lines represent normal simulations and simulations corrected for non-MNP urinary metabolites (des-methyl-fenitrothion and des-methyl-fenitro-oxon; ~40% of total metabolites). These simulations include corrections for active transport (see main text). *In vivo* data from [60,62], where total concentrations were measured in blood and urine. FNT: fenitrothion; MNP: 3-methyl-4-nitrophenol.

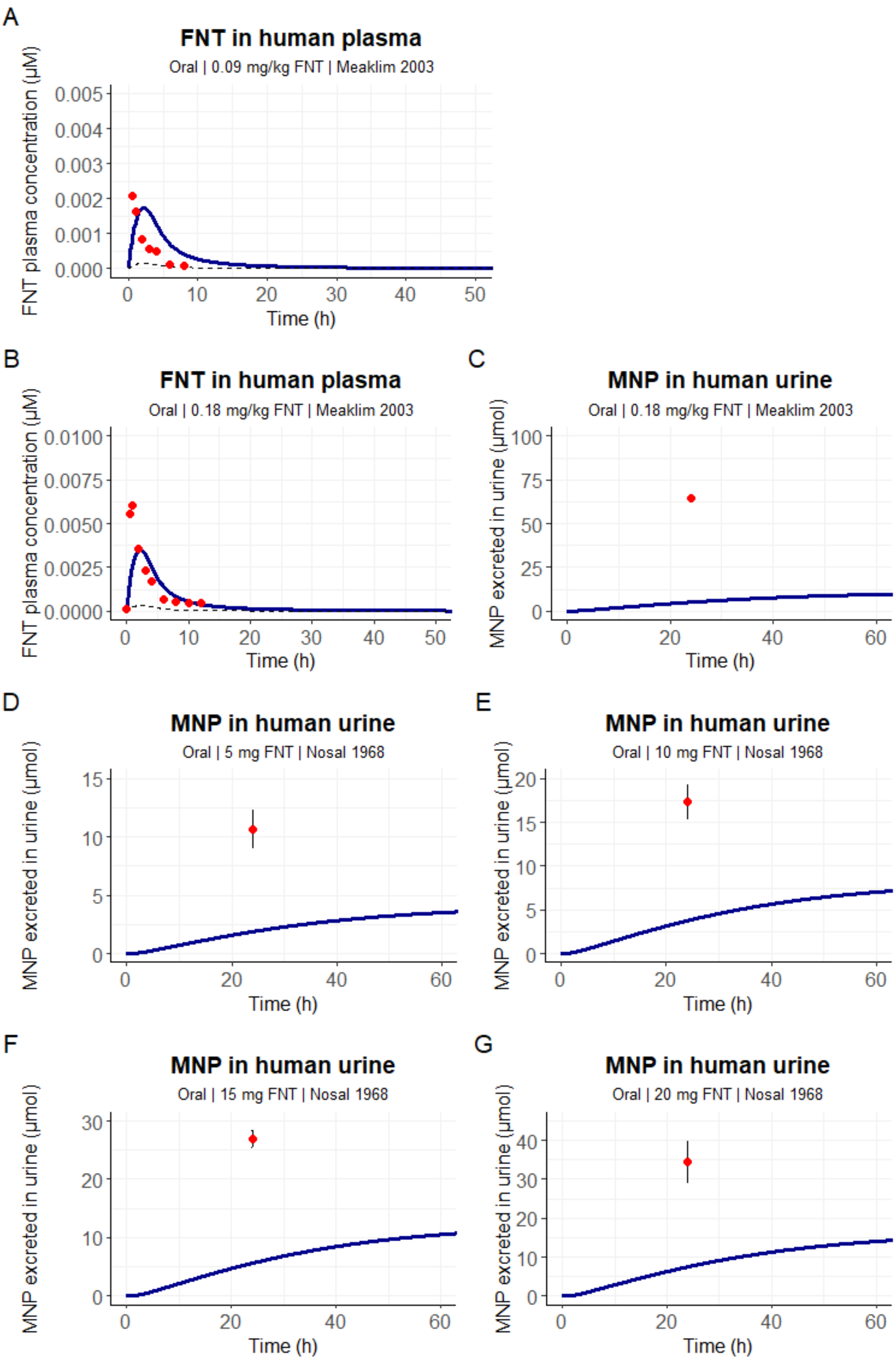

Figure S14: Simulated (lines) and observed (shapes) fenitrothion plasma concentration profiles (A, B) and MNP urinary excretion profiles (C-G) after oral fenitrothion exposure of humans to 0.09 or 0.18 mg/kg bw (A, B), to a total amount of 5, 10, 15, 20 mg (D, E, F, G). Dashed lines are simulated unbound concentrations of fenitrothion. These simulations include corrections for active transport (see materials and methods section in main text). *In vivo* data from [58,59], where total concentrations were measured in plasma and urine. FNT: fenitrothion; MNP: 3-methyl-4-nitrophenol.

3.2.4 Profenofos

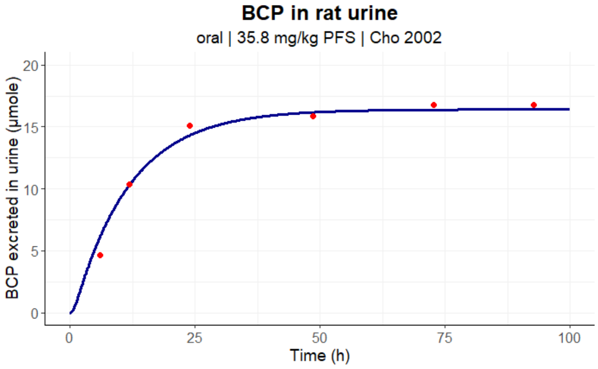

Figure S15: Simulated (lines) and observed (circles) urinary excretion of BCP after oral exposure of rats to 35.8 mg/kg bw profenofos in rats. These simulations include the correction for active transport (see materials and methods section in main text). *In vivo* data from [69], where the total concentration of BCP was measured in urine. PFS: profenofos; BCP: 4-bromo-2-chlorophenol.

3.2.5 Chlorfenvinphos

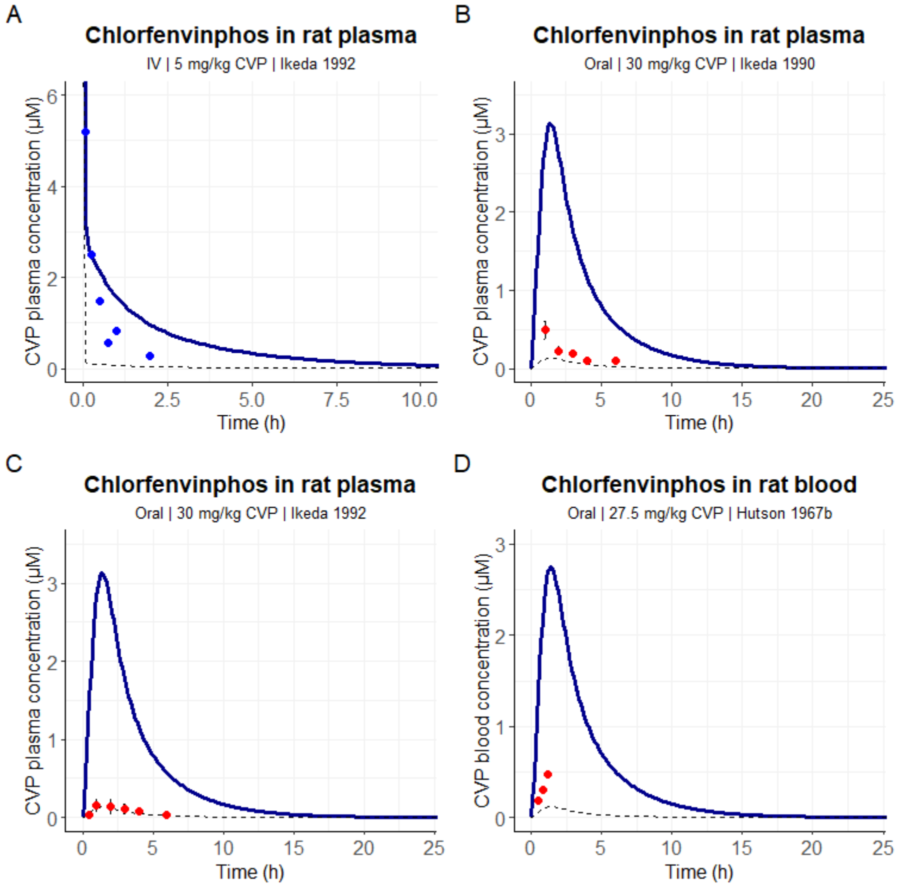

Figure S16: Simulated (lines) and observed (circles) blood (D) and plasma (A, B, C) concentration-time profiles after intravenous exposure (A) of rats to 5 mg/kg bw or oral exposure (B, C, D) to 27.5 or 30 mg/kg bw chlorfenvinphos. Solid and dashed lines represent total and unbound concentrations in blood/plasma, respectively. *In vivo* data from [66–68], where the studies state that total concentration is measured, however this is disputed (see materials and methods section in main text). CVP: chlorfenvinphos.

3.2.6 Dimethoate

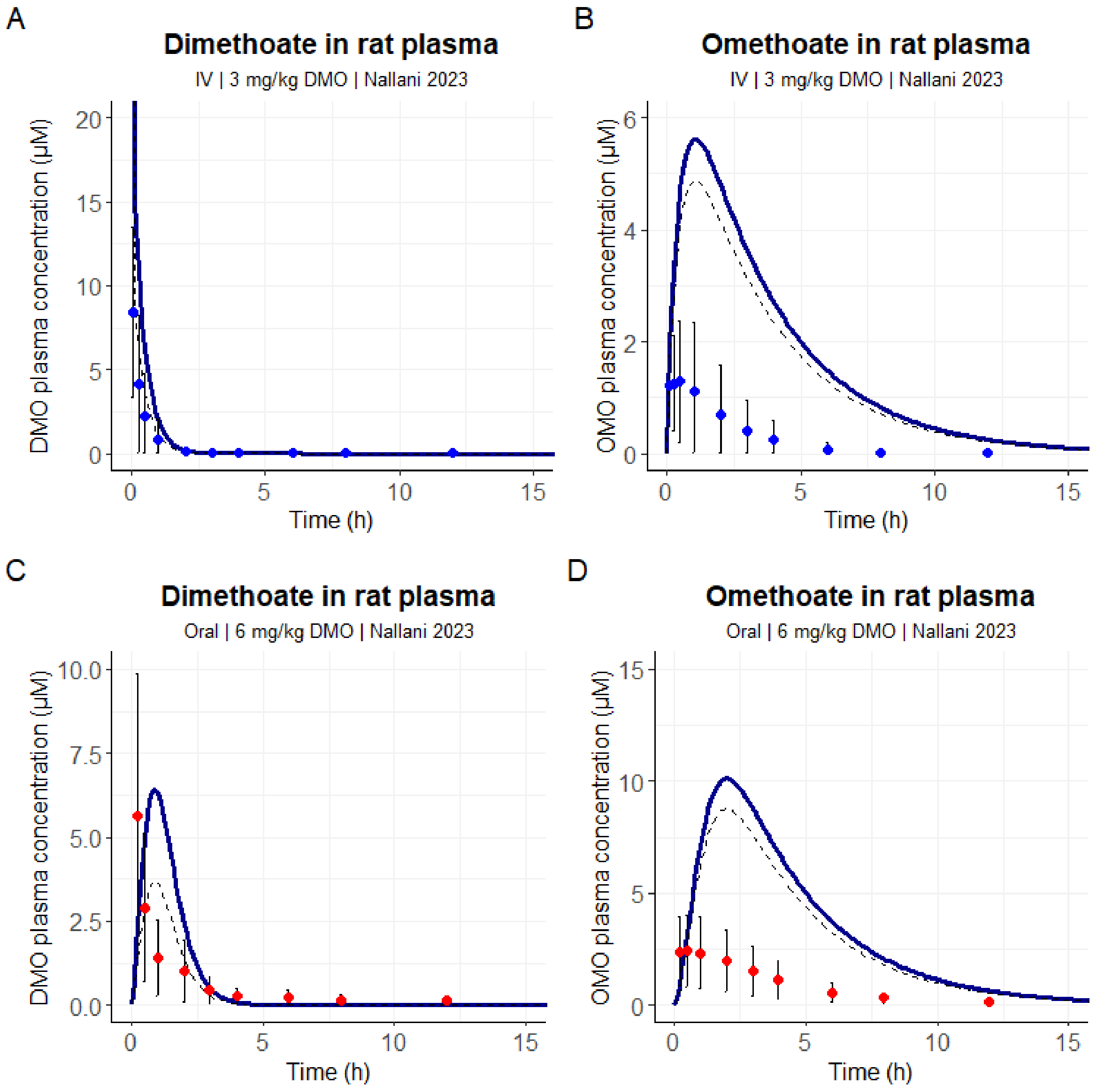

Figure S17: Simulated (lines) and observed (circles) plasma concentration-time profiles in rats after intravenous exposure (A, B) to 3 mg/kg bw or oral exposure (C, D) to 6 mg/kg bw chlorfenvinphos. Solid and dashed lines represent total and unbound concentrations in plasma, respectively. *In vivo* data from [41,57], where total plasma concentrations are measured. DMO: dimethoate; OMO: omethoate.

3.2.7 Methyl-parathion

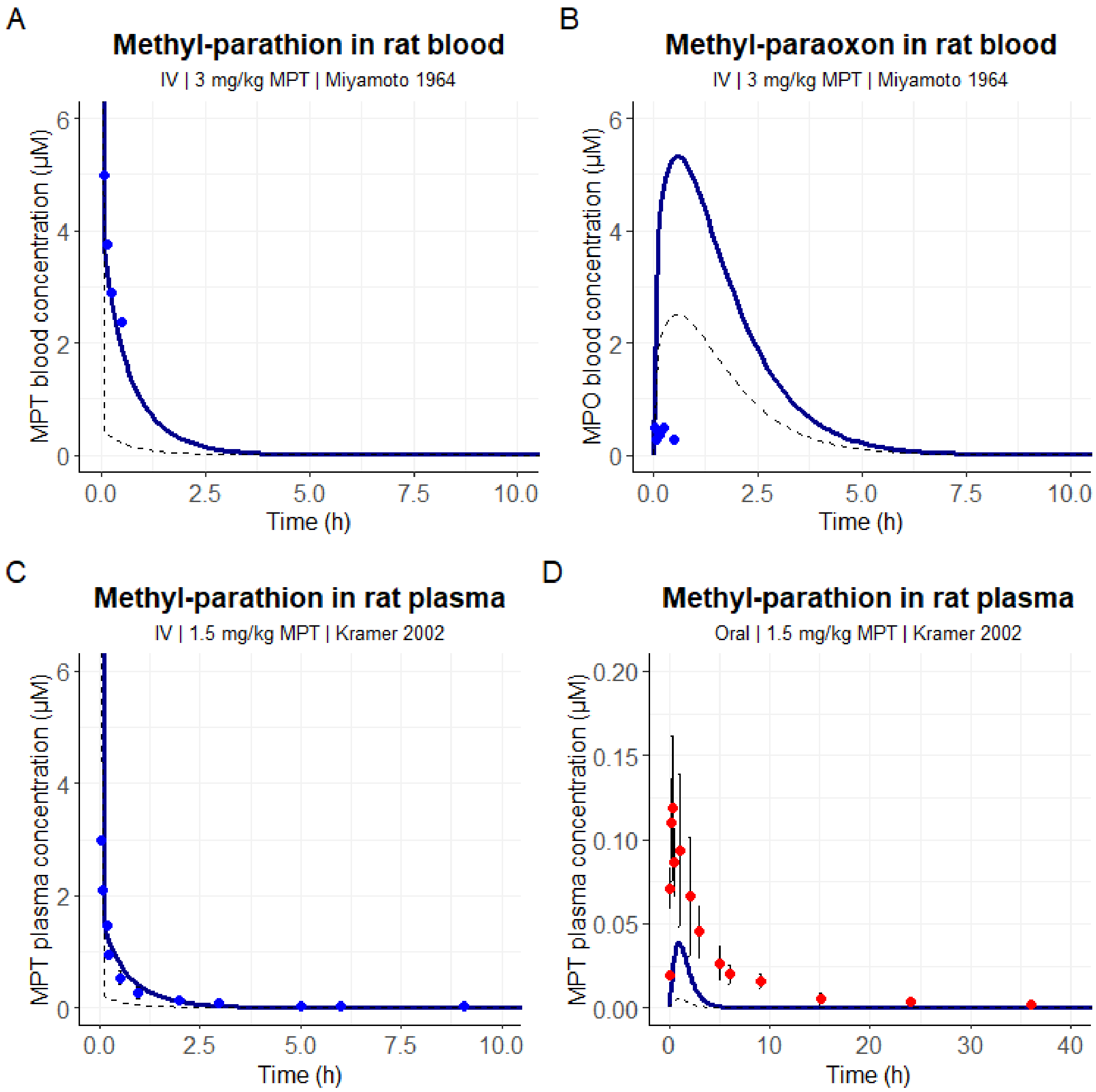

Figure S18: Simulated (lines) and observed (circles) fenitrothion (A, C, D) and fenitro-oxon (B) blood and plasma concentration-time profiles in rats after intravenous exposure (A, B, C) to 1.5 or 3 mg/kg bw, or oral exposure (D) to 1.5 mg/kg bw methyl-parathion. Solid and dashed lines represent total and unbound concentrations in blood/plasma, respectively. *In vivo* data from [60,63], where total plasma concentrations were measured. MPT: methyl-parathion.

3.2.8 Ethyl-parathion

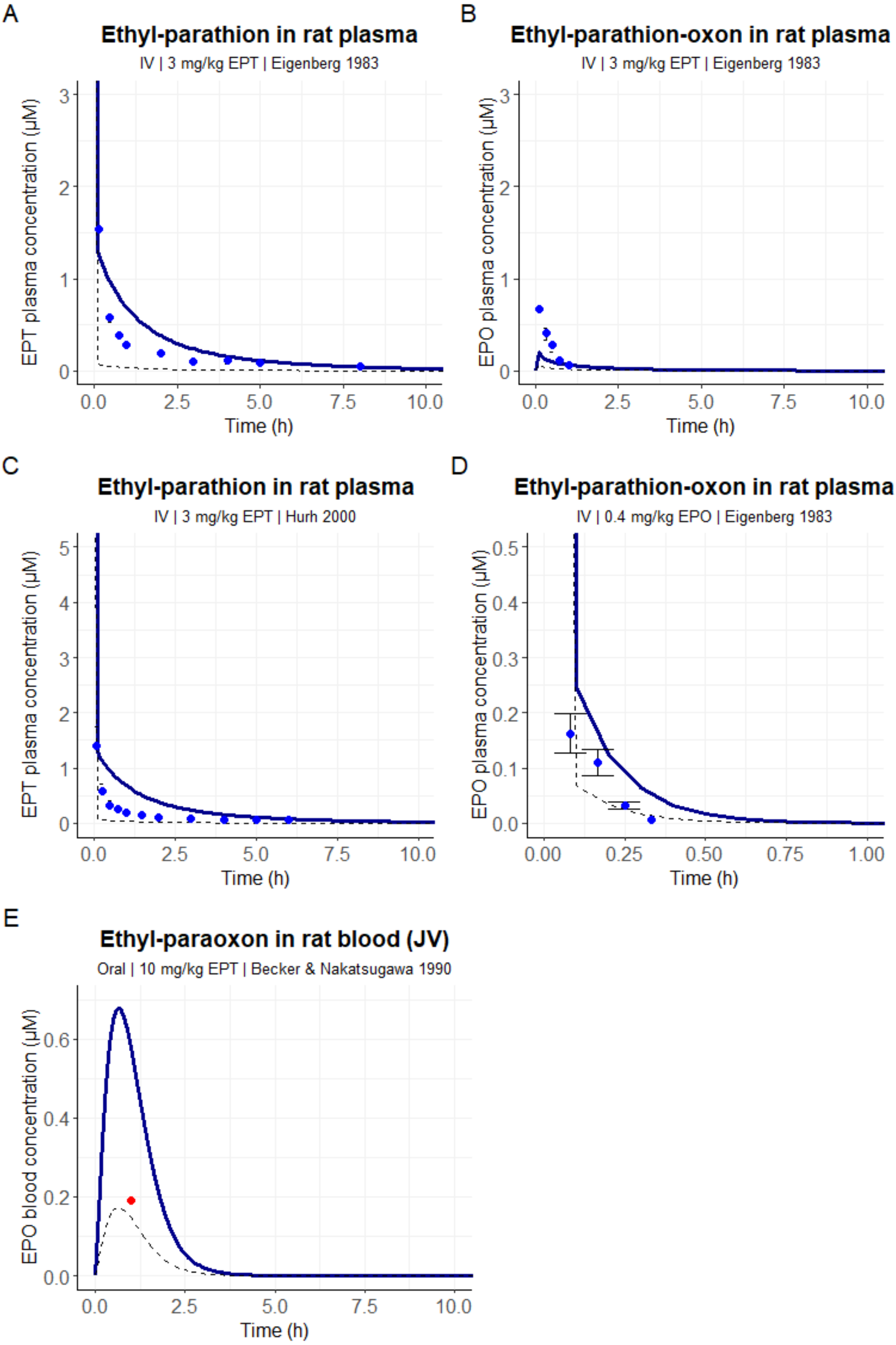

Figure S19: Simulated (lines) and observed (circles) ethyl-parathion (A, C) and ethyl-para-oxon (B, D, E) plasma and blood concentration-time profiles in rats after intravenous exposure to 3 or 10 mg/kg bw ethyl-parathion or 0.4 mg/kg bw ethyl-para-oxon. Solid and dashed lines represent total and unbound concentrations in blood/plasma, respectively. *In vivo* data from [64,65,70], where total plasma and blood concentrations were measured. EPT: ethyl-parathion; EPO: ethyl-paraoxon.

3.3 Sensitivity analysis

Local sensitivity analysis was performed for oral exposure to each of the considered pesticides. The description of the parameter abbreviations can be found in the sections 4.1 and 4.2 of this document.

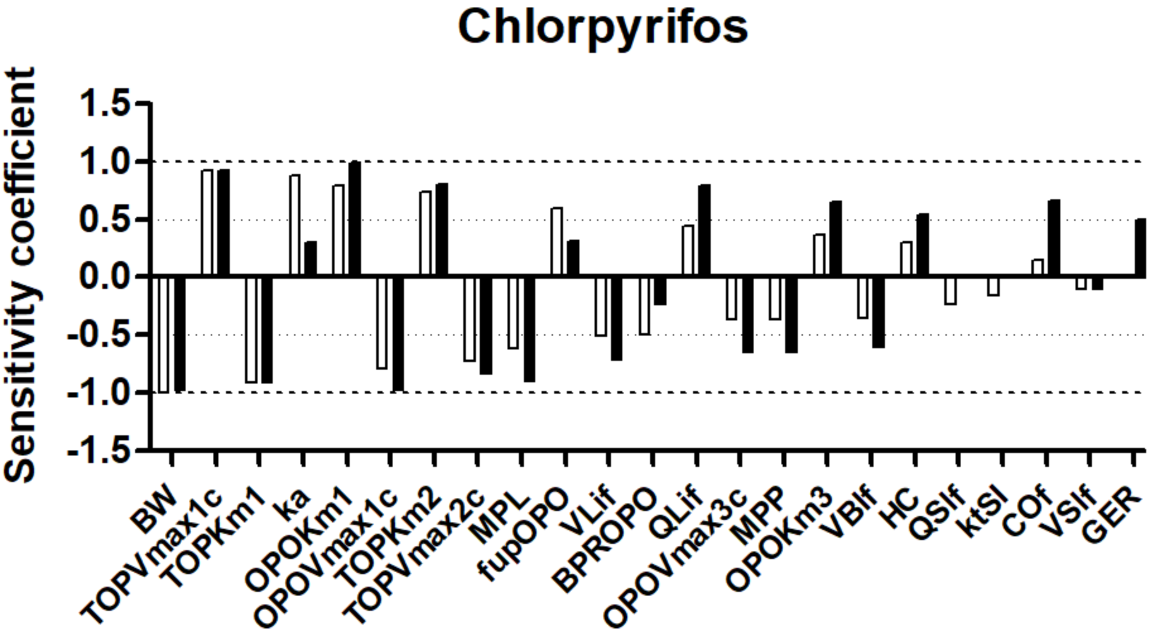

Figure S20: Local sensitivity analysis for oral exposure to 1 mg/kg bodyweight chlorpyrifos in humans (white bars) and rats (black bars). Parameters were changed by 1% to test the relative effect on the free concentration of chlorpyrifos-oxon in blood. Only parameters with a sensitivity coefficient below -0.1 or above 0.1 are included. Parameters are ordered from high to low sensitivity coefficient for humans.

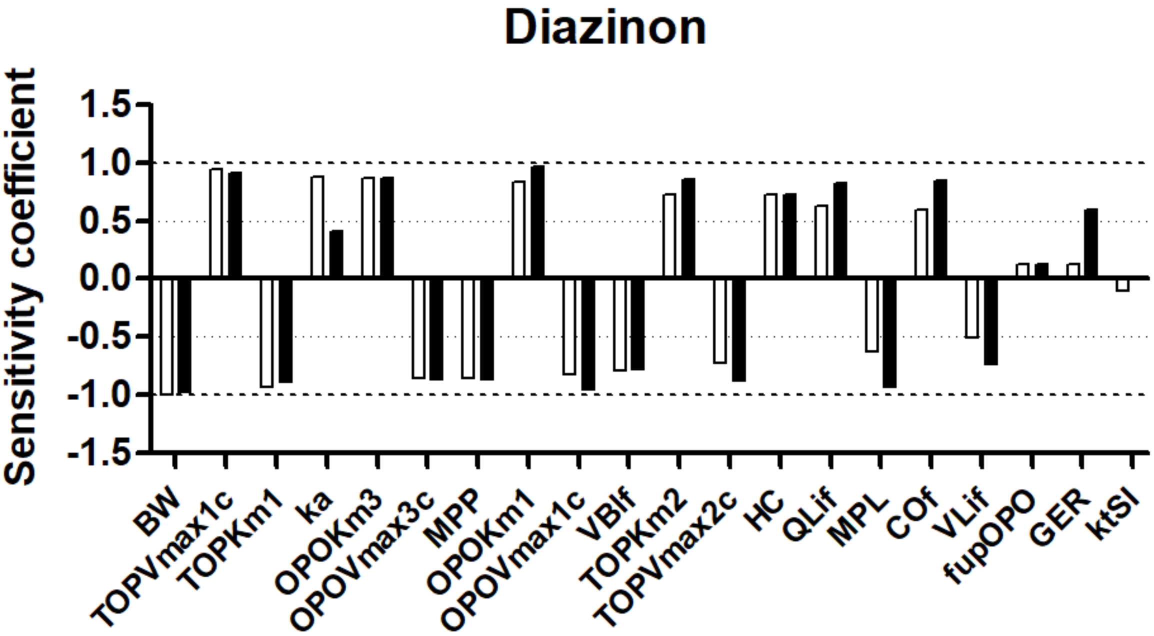

Figure S21: Local sensitivity for oral exposure to 1 mg/kg bodyweight diazinon in humans (white bars) and rats (black bars). Parameters were changed by 1% to test the relative effect on the free concentration of diazinon-oxon in blood. Only parameters with a sensitivity coefficient below -0.1 or above 0.1 are included. Parameters are ordered from high to low sensitivity coefficient for humans.

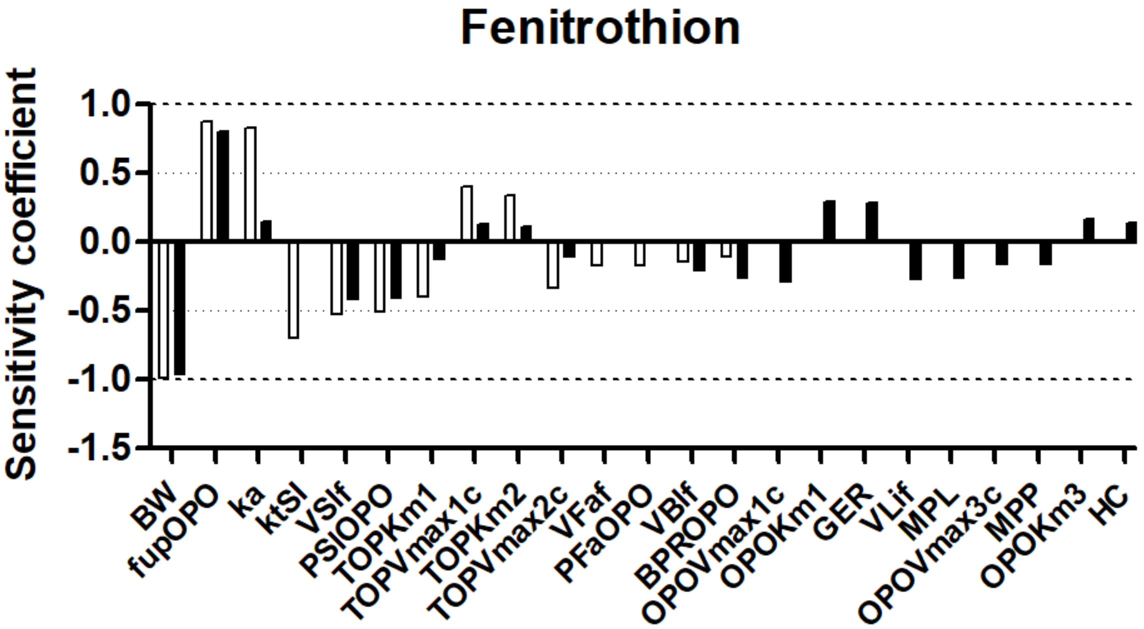

Figure S22: Local sensitivity for oral exposure to 1 mg/kg bodyweight fenitrothion in humans (white bars) and rats (black bars). Parameters were changed by 1% to test the relative effect on the free concentration of fenitro-oxon in blood. Only parameters with a sensitivity coefficient below -0.1 or above 0.1 are included. Parameters are ordered from high to low sensitivity coefficient for humans.

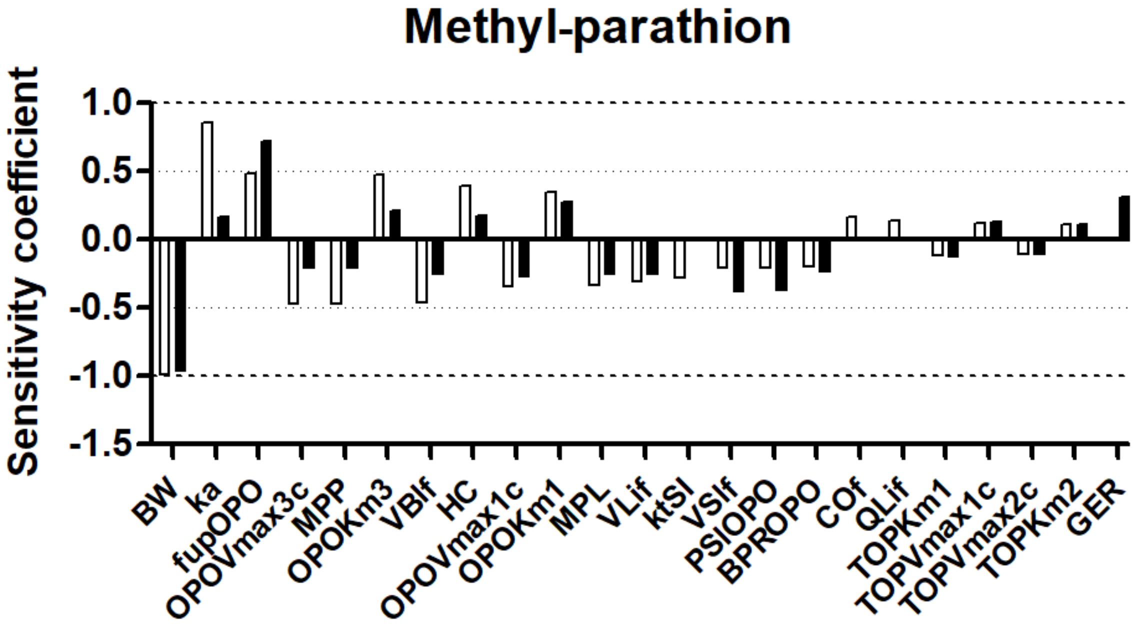

Figure S23: Local sensitivity for oral exposure to 1 mg/kg bodyweight methyl-parathion in humans (white bars) and rats (black bars). Parameters were changed by 1% to test the relative effect on the free concentration of diazinon-oxon in blood. Only parameters with a sensitivity coefficient below -0.1 or above 0.1 are included. Parameters are ordered from high to low sensitivity coefficient for humans.

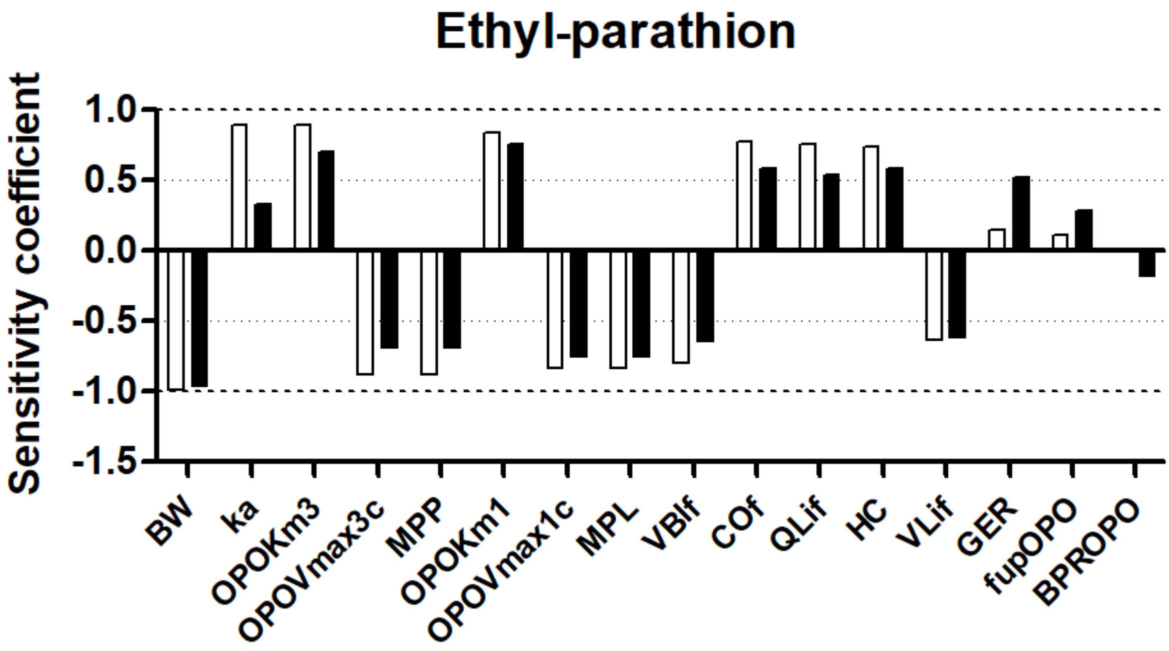

Figure S24: Local sensitivity for oral exposure to 1 mg/kg bodyweight methyl-parathion in humans (white bars) and rats (black bars). Parameters were changed by 1% to test the relative effect on the free concentration of diazinon-oxon in blood. Only parameters with a sensitivity coefficient below -0.1 or above 0.1 are included. Parameters are ordered from high to low sensitivity coefficient for humans.

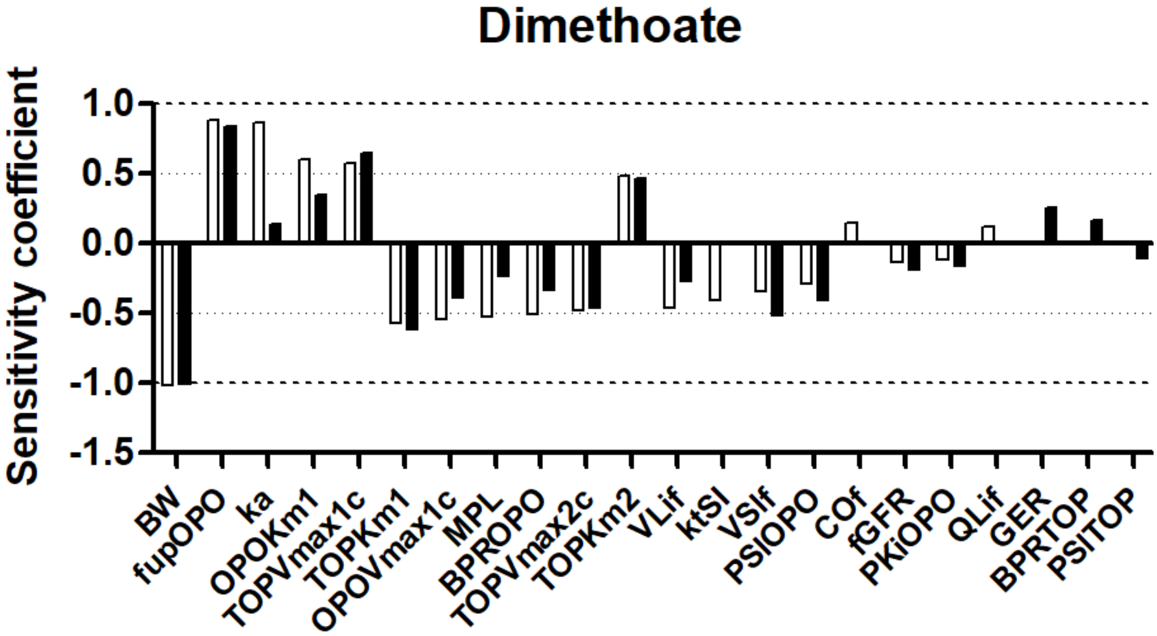

Figure S25: Local sensitivity for oral exposure to 1 mg/kg bodyweight methyl-parathion in humans (white bars) and rats (black bars). Parameters were changed by 1% to test the relative effect on the free concentration of diazinon-oxon in blood. Only parameters with a sensitivity coefficient below -0.1 or above 0.1 are included. Parameters are ordered from high to low sensitivity coefficient for humans.

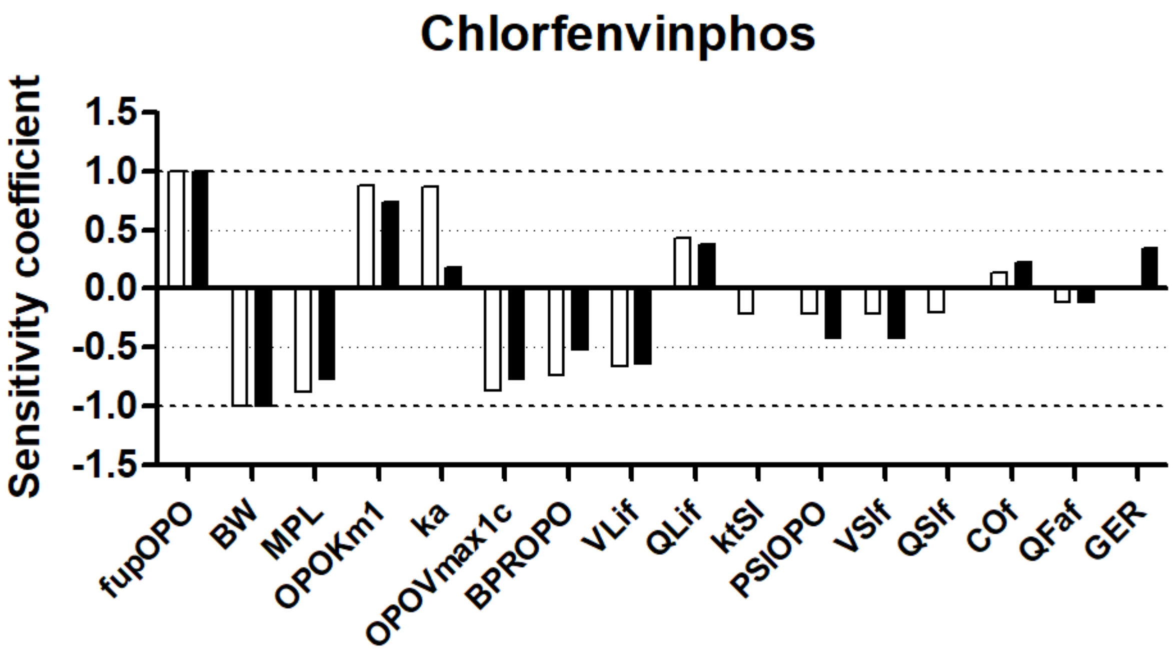

Figure S26: Local sensitivity for oral exposure to 1 mg/kg bodyweight ethyl-parathion in humans (white bars) and rats (black bars). Parameters were changed by 1% to test the relative effect on the free concentration of diazinon-oxon in blood. Only parameters with a sensitivity coefficient below -0.1 or above 0.1 are included. Parameters are ordered from high to low sensitivity coefficient for humans.

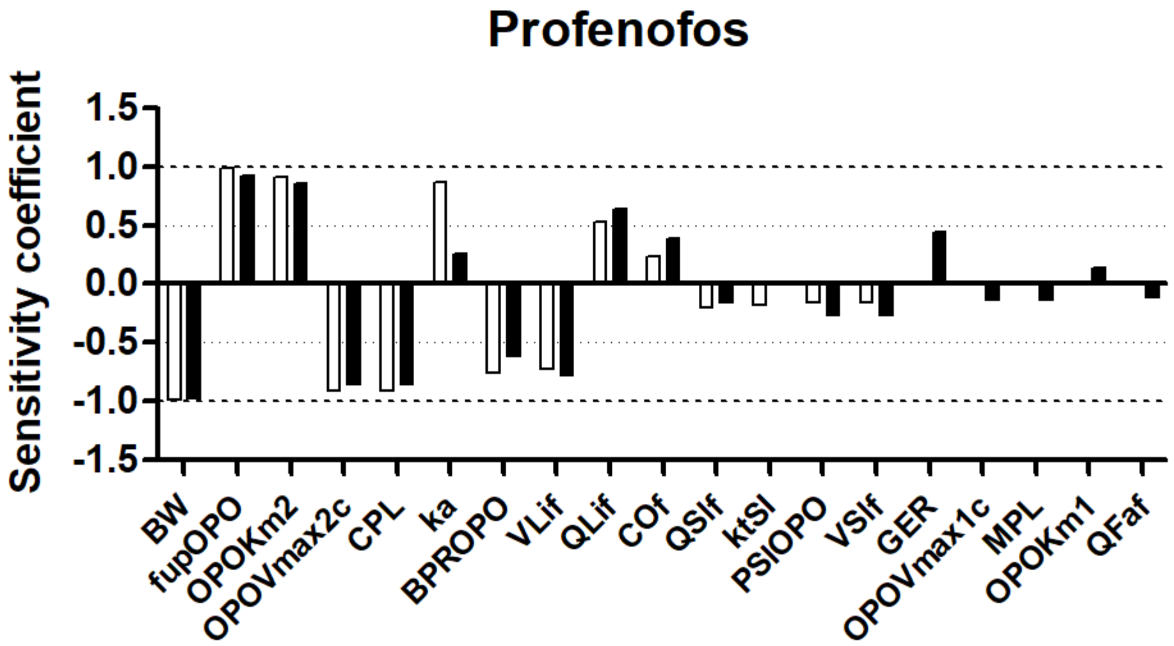

Figure S27: Local sensitivity for oral exposure to 1 mg/kg bodyweight profenofos in humans (white bars) and rats (black bars). Parameters were changed by 1% to test the relative effect on the free concentration of profenofos in blood. Only parameters with a sensitivity coefficient below -0.1 or above 0.1 are included. Parameters are ordered from high to low sensitivity coefficient for humans.

4. PBK model

4.1 General abbreviations in model

Abbreviation	Desciption
TOP	Thio-organophosphate
OPO	Organophosphate-oxon
OPM	Organophosphate (urinary) metabolite
Fa	Fat
Br	Brain
Bl	Blood
Ki	Kidneys
Ri	Richly perfused
Sl	Slowly perfused
Q	Perfusion
V	Volume
P	Tissue:plasma partition coefficient
C	Concentration
A	Amount

4.2 Model parameter and variable name list

Parameter	Description
BW	Bodyweight of the organism in kg
VFa	Volume of fat tissue in organism in L
VFaf	Volume fraction of fat tissues in organism in L/kg bodyweight
VLi	Volume of liver tissues in organism in L
VLif	Volume fraction of liver tissue in organism in L/kg bodyweight
VBr	Volume of brain tissue in organism in L
VBrf	Volume fraction of brain tissue in organism in L/kg bodyweight
VKi	Volume of kidney tissue in organism in L
VKif	Volume fraction of kidney tissue in organism in L/kg bodyweight
VBl	Volume of blood tissue in organism in L
VBlf	Volume fraction of blood tissue in organism in L/kg bodyweight
VRi	Volume of richly perfused tissues in organism in L
VRif	Volume fraction of richly perfused tissues in organism in L/kg bodyweight
VSl	Volume of slowly perfused tissues in organism in L
VSlf	Volume fraction of slowly perfused tissues tissue in organism in L/kg bodyweight
CO	Total cardiac output of the organism in L/h
COf	Cardiac output fraction of the organism in L/h/kg bodyweight
QFa	Perfusion to fat tissues in organism in L/h
QFaf	Fraction of CO flowing to fat tissues
QLi	Perfusion to liver tissue in organism in L/h
QLif	Fraction of CO flowing to liver tissue
QBr	Perfusion to brain tissue in organism in L/h
QBrf	Fraction of CO flowing to brain tissue
QKi	Perfusion to kidney tissue in organism in L/h
QKif	Fraction of CO flowing to kidney tissue
QRi	Perfusion to richly perfused tissues in organism in L/h
QRif	Fraction of CO flowing to richly perfused tissues
QSl	Perfusion to slowly perfused tissues in organism in L/h
QSlf	Fraction of CO flowing to slowly perfused tissues
TOPVmax1	Scaled Vmax parameter for metabolism of TOP to OPO in liver microsomes
TOPVmax1c	Unscaled Vmax parameter for metabolism of TOP to OPO in liver microsomes
TOPVmax2	Scaled Vmax parameter for metabolism of TOP to OPM in liver microsomes
TOPVmax2c	Unscaled Vmax parameter for metabolism of TOP to OPM in liver microsomes

OPOVmax1	Scaled Vmax parameter for metabolism of OPO to OPM in liver microsomes
OPOVmax1c	Unscaled Vmax parameter for metabolism of OPO to OPM in liver microsomes
OPOVmax2	Scaled Vmax parameter for metabolism of TOP to OPM in liver cytosol
OPOVmax2c	Unscaled Vmax parameter for metabolism of TOP to OPM in liver cytosol
OPOVmax3	Scaled Vmax parameter for metabolism of TOP to OPM in blood plasma
OPOVmax3c	Unscaled Vmax parameter for metabolism of TOP to OPM in blood plasma
TOPKm1	Km parameter for metabolism TOP to OPO in liver microsomes
TOPKm2	Km parameter for metabolism TOP to OPM in liver microsomes
OPOKm1	Km parameter for metabolism OPO to OPM in liver microsomes
OPOKm2	Km parameter for metabolism OPO to OPM in liver cytosol
OPOKm3	Km parameter for metabolism OPO to OPM in blood plasma
AMR1	Amount metabolized of TOP to OPO in liver microsomes
AMR2	Amount metabolized of TOP to OPM in liver microsomes
AMR3	Amount metabolized of OPO to OPM in liver microsomes
AMR4	Amount metabolized of TOP to OPM in liver cytosol
AMR5	Amount metabolized of TOP to OPM in blood plasma
MPL	Scaling factor: Mg microsomal protein per gram of liver (mg/g liver)
CPL	Scaling factor: Mg cytosolic protein per gram of liver (mg/g liver)
MPP	Scaling factor: Mg plasma protein per gram of plasma (mg/g plasma)
HC	Hematocrit (volume of red blood cells/volume of total blood)
GFR	Glomerular filtration rate
fGFR	GFR per kg of bodyweight
BPRTOP	Blood plasma ratio for TOP
BPROPO	Blood plasma ratio for OPO
OPMavgBPR	Blood plasma ratio for OPM
AFaTOP	Amount of TOP in fat tissues in µmol
AFaOPO	Amount of OPO in fat tissues in µmol
AFaOPM	Amount of OPM in fat tissues in µmol
CFaTOP	Concentration of TOP in fat tissues in µM
CFaOPO	Concentration of OPO in fat tissues in µM
CFaOPM	Concentration of OPM in fat tissues in µM
CVFaTOP	Concentration of TOP in venous blood flowing from fat tissues
CVFaOPO	Concentration of OPO in venous blood flowing from fat tissues
CVFaOPM	Concentration of OPM in venous blood flowing from fat tissues
PFaTOP	Plasma:tissue partition coefficients for TOP in fat tissues
PFaOPO	Plasma:tissue partition coefficients for OPO in fat tissues
PFaOPM	Plasma:tissue partition coefficients for OPM in fat tissues
ALiTOP	Amount of TOP in liver tissues in µmol
ALiOPO	Amount of OPO in liver tissues in µmol
ALiOPM	Amount of OPM in liver tissues in µmol
CLiTOP	Concentration of TOP in liver tissues in µM
CLiOPO	Concentration of OPO in liver tissues in µM
CLiOPM	Concentration of OPM in liver tissues in µM
CVLiTOP	Concentration of TOP in venous blood flowing from liver tissues in µM
CVLiOPO	Concentration of OPO in venous blood flowing from liver tissues in µM
CVLiOPM	Concentration of OPM in venous blood flowing from liver tissues in µM
PLiTOP	Plasma:tissue partition coefficients for TOP in liver tissues
PLiOPO	Plasma:tissue partition coefficients for OPO in liver tissues
PLiOPM	Plasma:tissue partition coefficients for OPM in liver tissues
ABrTOP	Amount of TOP in brain tissues in µmol
ABrOPO	Amount of OPO in brain tissues in µmol
ABrOPM	Amount of OPM in brain tissues in µmol
CBrTOP	Concentration of TOP in brain tissues in µM
CBrOPO	Concentration of OPO in brain tissues in µM
CBrOPM	Concentration of OPM in brain tissues in µM
CVBrTOP	Concentration of TOP in venous blood flowing from brain tissues in µM
CVBrOPO	Concentration of OPO in venous blood flowing from brain tissues in µM
CVBrOPM	Concentration of OPM in venous blood flowing from brain tissues in µM

PBrTOP	Plasma:tissue partition coefficients for TOP in brain tissues
PBrOPO	Plasma:tissue partition coefficients for OPO in brain tissues
PBrOPM	Plasma:tissue partition coefficients for OPM in brain tissues
ARiTOP	Amount of TOP in richly perfused tissues in μmol
ARiOPO	Amount of OPO in richly perfused tissues in μmol
ARiOPM	Amount of OPM in richly perfused tissues in μmol
CRiTOP	Concentration of TOP in richly perfused tissues in μM
CRiOPO	Concentration of OPO in richly perfused tissues in μM
CRiOPM	Concentration of OPM in richly perfused tissues in μM
CVRiTOP	Concentration of TOP in venous blood flowing from richly perfused tissues in μM
CVRiOPO	Concentration of OPO in venous blood flowing from richly perfused tissues in μM
CVRiOPM	Concentration of OPM in venous blood flowing from richly perfused tissues in μM
PRiTOP	Plasma:tissue partition coefficients for TOP in richly perfused tissues
PRiOPO	Plasma:tissue partition coefficients for OPO in richly perfused tissues
PRiOPM	Plasma:tissue partition coefficients for OPM in richly perfused tissues
ASlTOP	Amount of TOP in slowly perfused tissues in μmol
ASlOPO	Amount of OPO in slowly perfused tissues in μmol
ASlOPM	Amount of OPM in slowly perfused tissues in μmol
CSlTOP	Concentration of TOP in slowly perfused tissues in μM
CSlOPO	Concentration of OPO in slowly perfused tissues in μM
CSlOPM	Concentration of OPM in slowly perfused tissues in μM
CVSlTOP	Concentration of TOP in venous blood flowing from slowly perfused tissues in μM
CVSlOPO	Concentration of OPO in venous blood flowing from slowly perfused tissues in μM
CVSlOPM	Concentration of OPM in venous blood flowing from slowly perfused tissues in μM
PSlTOP	Plasma:tissue partition coefficients for TOP in slowly perfused tissues
PSlOPO	Plasma:tissue partition coefficients for OPO in slowly perfused tissues
PSlOPM	Plasma:tissue partition coefficients for OPM in slowly perfused tissues
ABlTOP	Amount of TOP in blood in μmol
ABlOPO	Amount of OPO in blood in μmol
ABlOPM	Amount of OPM in blood in μmol
CBlTOP	Concentration of TOP in blood in μM
CBlOPO	Concentration of OPO in blood in μM
CBlOPM	Concentration of OPM in blood in μM
CPlTOP	Concentration of TOP in blood plasma in μM
CPlOPO	Concentration of OPO in blood plasma in μM
CPlOPM	Concentration of OPM in blood plasma in μM
CPfTOP	Free unbound concentration of TOP in blood plasma in μM
CPfOPO	Free unbound concentration of OPO in blood plasma in μM
CPfOPM	Free unbound concentration of OPM in blood plasma in μM
fupTOP	Fraction unbound in plasma for TOP
fupOPO	Fraction unbound in plasma for OPO
OPMavgfup	Fraction unbound in plasma for OPM, averaged between different conjugates and derivatives
AKiTOP	Amount of TOP in kidney tissues in μmol
AKiOPO	Amount of OPO in kidney tissues in μmol
AKiOPM	Amount of OPM in kidney tissues in μmol
CKiTOP	Concentration of TOP in kidney tissues in μM
CKiOPO	Concentration of OPO in kidney tissues in μM
CKiOPM	Concentration of OPM in kidney tissues in μM
CVKiTOP	Concentration of TOP in venous blood flowing from kidney tissues in μM
CVKiOPO	Concentration of OPO in venous blood flowing from kidney tissues in μM
CVKiOPM	Concentration of OPM in venous blood flowing from kidney tissues in μM
PKiTOP	Plasma:tissue partition coefficients for TOP in kidney tissues
PKiOPO	Plasma:tissue partition coefficients for OPO in kidney tissues
PKiOPM	Plasma:tissue partition coefficients for OPM in kidney tissues
ACRTOP	Amount cleared renally for TOP in μmol
ACROPO	Amount cleared renally for OPO in μmol
ACROPM	Amount cleared renally for OPM in μmol

Ast	Amount of administered compound (TOP or OPO) in the stomach
GER	Gastric Emptying Rate in fraction /h
Ain1	Amount of administered compound (TOP or OPO) in intestinal compartments 1
Ain2	Amount of administered compound (TOP or OPO) in intestinal compartments 2
Ain3	Amount of administered compound (TOP or OPO) in intestinal compartments 3
Ain4	Amount of administered compound (TOP or OPO) in intestinal compartments 4
Ain5	Amount of administered compound (TOP or OPO) in intestinal compartments 5
Ain6	Amount of administered compound (TOP or OPO) in intestinal compartments 6
Ain7	Amount of administered compound (TOP or OPO) in intestinal compartments 7
Acol	Amount of administered compound (TOP or OPO) in colon
Afec	Amount of administered compound (TOP or OPO) in feces
KtSI	Small intestine transit rate constant in fraction /h
KtCol	Colon transit rate constant in fraction /h
ka	Absorption rate constant in fraction /h
TAMR1	Cumulative amount of TOP metabolized to OPO in liver microsomes in μmol
TAMR2	Cumulative amount of TOP metabolized to OPM in liver microsomes in μmol
TAMR3	Cumulative amount of OPO metabolized to OPM in liver microsomes in μmol
TAMR4	Cumulative amount of OPO metabolized to OPM in liver cytosol in μmol
TAMR5	Cumulative amount of OPO metabolized to OPM in plasma in μmol
TOP	If 1, administered compound is a thiophosphate; if 0, administered compound is a phosphate-oxon.
TACRTOP	Cumulative amount cleared renally for TOP in μmol
TACROPO	Cumulative amount cleared renally for OPO in μmol
TACROPM	Cumulative amount cleared renally for OPM in μmol

4.3 Mass balance equations

Tissue concentration and concentration in venous blood flowing from tissue

$CTi = \frac{ATi}{VTi}$ (Eq. 1) $CVTi = \frac{CTi}{PTi} \times BPR$ (Eq. 2)

CTi: Concentration in tissue; ATi: Amount of compound in tissue; VTi: Volume of tissue; CVTi: Concentration in venous blood flowing from tissue; PTi: tissue:plasma partition coefficient; BPR: blood-plasma-ratio.

Used differential equations for calculating the change in the amount of a compound in a tissue:

Non-clearing tissues

$\frac{d}{dt}(ATi) = QTi \times (CBl - CVTi)$ (Eq. 3)

ATi: Amount of compound in tissue; QTi: blood flow to tissue; CBl: concentration of compound in blood; CVTi: concentration of compound in venous blood flowing from tissue.

Liver

$\frac{d}{dt}(ALi) = AAb + QLi \times (CBl - CVLi) + \sum AM$ (Eq. 4)

ALi: amount of compound in liver; AAb: Amount of compound absorbed; QLi: blood flow to liver; CBl: concentration of compound in blood; CVLi: concentration of compound in venous blood flowing from liver; AM: amount of compound produced (positive) or cleared (negative) in metabolic reactions in liver.

Blood

$\frac{d}{dt}(ABl) = \sum(QTi \times CVTi) - CBl \times \sum QTi - \sum AM$ (Eq. 5)

ALi: amount of compound in blood; QTi: blood flow to tissue; CVTi: concentration of compound in venous blood flowing from tissue; CBl: concentration of compound in blood; AM: amount of compound produced (positive) or cleared (negative) in metabolic reactions in blood.

Kidney

$\frac{d}{dt}(AKi) = QKi \times (CBl - CVKi) - ACR$ (Eq. 6)

AKi: Amount of compound in kidney; QKi: blood flow to kidney; CBl: concentration of compound in blood; CVKi: concentration of compound in venous blood flowing from kidney; ACR: Amount of compound cleared renally.

Metabolism and excretion

While metabolism of OTP only takes place in the liver, OPOs are metabolized both in the liver and plasma. Furthermore, every compound is able to be cleared by the kidneys into the urine. Liver metabolism is incorporated in the model using Michaelis-Menten kinetics (equation 7) [71]. Meanwhile, renal clearance is incorporated using the glomerular filtration rate (GFR; equation 8).

$AM = \frac{V_{max} \times CVL}{K_m + CVL}$ (Eq. 7) $ACR = GFR \times CK \times fu_p$ (Eq. 8)

In the above equations, AM and ACR are the amount metabolized in the liver, and the amount cleared by the kidney respectively. Furthermore, CVL and CK are the venous liver and the kidney concentration respectively. The Michaelis-Menten parameters, V_{max} and K_m, are the maximum velocity of the enzymatic reaction in µmol/h/gram of tissue (liver or blood) and the Michaelis constant in µM. These parameters are scaled from microsomal incubation data and can be found in literature (see **SI table S3**), or acquired experimentally. The GFR is expressed in L/h and the fu_p is the unbound, or free, fraction of a compound in plasma. This parameter is used as a surrogate for the free, unbound fraction of compound in the respective tissues. The amount of compound available for metabolism is approximated using the venous concentration of a tissue [72].

4.5 Model Code

The model was developed and run in R studio (version 1.4.1717; R programming language version 4.1.0) and using the RxODE2 package [73]. The PBK model is able to simulate ADME kinetics for OTPs, OPOs and their respective metabolites.

```
# PHYSIOLOGICAL PARAMETERS
# Tissue volumes (from fraction of BW)
VFa   <-  BW * VFaf;   # Fat tissue
VLi   <-  BW * VLif;   # Liver tissue
VBr   <-  BW * VBrf;   # Brain tissue
VKi   <-  BW * VKif;   # Kidney tissue
VBl   <-  BW * VBlf;   # Blood tissue
VRi   <-  BW * VRif;   # Richly perfused tissue
VSl   <-  BW * VSlf;   # Slowly perfused tissue

CO    <- BW * COf;     # Cardiac output

# Tissue perfusion (from fraction of CO)
QFa   <-  CO * QFaf;   # Fat tissue
QLi   <-  CO * QLif;   # Liver tissue
QBr   <-  CO * QBrf;   # Brain tissue
QKi   <-  CO * QKif;   # Kidney tissue
QRi   <-  CO * QRif;   # Richly perfused tissue
QSl   <-  CO * QSlf;   # Slowly perfused tissue

# Metabolism scaling: scale to total metabolizing protein based on Liver or blood volume; scale to correct units (umol/h)
TOPVmax1  <- TOPVmax1c / 1000 * 60 * MPL * 1000 * VLi; # umol/h/mg microsomal protein * mg microsomal protein = umol/h
TOPVmax2  <- TOPVmax2c / 1000 * 60 * MPL * 1000 * VLi;
OPOVmax1  <- OPOVmax1c / 1000 * 60 * MPL * 1000 * VLi;
OPOVmax2  <- OPOVmax2c / 1000 * 60 * CPL * 1000 * VLi;
# umol/h/mg cytosolic protein * mg cytosolic protein = umol/h

OPOVmax3  <- OPOVmax3c / 1000 * 60 * MPP * 1000 * VBl * (1-HC);
# umol/h/mg plasma protein * mg plasma protein = umol/h

# Renal excretion: Scale glomerular filtration rate with bodyweight
GFR     <- BW * GFRf;

# COMPARTMENTS
# Concentrations in each compartment and the associated venous blood is calculated
# Concentration in compartment (uM) = Amount in compartment (umol) / Volume of compartment (L)
# Concentration in venous blood of compartment = (Concentration in compartment / tissue:plasma partition coefficient) * Blood plasma ratio
# This is done for each compartment (Fat, Liver, Brain, Richly perfused tissues, Slowly perfused tissues, Kidney)
# and each compound (TOP, OPO and OPM)

# Concentrations fat and venous blood from fat
CFaTOP   <- AFaTOP / VFa;
CVFaTOP  <- (CFaTOP / PFaTOP)*BPRTOP;

CFaOPO   <- AFaOPO / VFa;
```

```
CVFaOPO  <- (CFaOPO / PFaOPO)*BPROPO;

CFaOPM   <- AFaOPM / VFa;
CVFaOPM  <- (CFaOPM / PFaOPM)*OPMavgBPR;


# Concentrations in liver and venous blood from liver
CLiTOP   <- ALiTOP / VLi;
CVLiTOP  <- (CLiTOP / PLiTOP)*BPRTOP;

CLiOPO   <- ALiOPO / VLi;
CVLiOPO  <- (CLiOPO / PLiOPO)*BPROPO;

CLiOPM   <- ALiOPM / VLi;
CVLiOPM  <- (CLiOPM / PLiOPM)*OPMavgBPR;

# Metabolism in liver according to Michaelis Menten kinetics
# (Vmax * available substrate concentration) / (Km + available substrate concentration)
# Available substrate concentration is estimated by concentration in the venous blood leaving the
liver, CVLi, as a surrogate
AMR1    <- (TOPVmax1 * CVLiTOP) / (TOPKm1 + CVLiTOP);
# Amount metabolized from TOP to OPO in liver microsomes by CYP450

AMR2    <- (TOPVmax2 * CVLiTOP) / (TOPKm2 + CVLiTOP);
# Amount metabolized from TOP to OPM in liver microsomes by CYP450

AMR3    <- (OPOVmax1 * CVLiOPO) / (OPOKm1 + CVLiOPO);
# Amount metabolized from OPO to OPM in liver microsomes by CYP and PON1

AMR4    <- (OPOVmax2 * CVLiOPO) / (OPOKm2 + CVLiOPO);
# Amount metabolized from OPO to OPM in liver cytosol by CYP and PON1

# Concentrations brain and venous blood from brain
CBrTOP  <- ABrTOP / VBr;
CVBrTOP <- CBrTOP / PBrTOP*BPRTOP;

CBrOPO  <- ABrOPO / VBr;
CVBrOPO <- CBrOPO / PBrOPO*BPROPO;

CBrOPM  <- ABrOPM / VBr;
CVBrOPM <- CBrOPM / PBrOPM*OPMavgBPR;

# Concentration richly perfused tissues and venous blood from richly perfused tissues
CRiTOP   <- ARiTOP / VRi;
CVRiTOP  <- CRiTOP / PRiTOP*BPRTOP;

CRiOPO   <- ARiOPO / VRi;
CVRiOPO  <- CRiOPO / PRiOPO*BPROPO;

CRiOPM   <- ARiOPM / VRi;
CVRiOPM  <- CRiOPM / PRiOPM*OPMavgBPR;

# Concentration slowly perfused tissues and venous blood from slowly perfused tissues
CSlTOP   <- ASlTOP / VSl;
CVSlTOP  <- CSlTOP / PSlTOP*BPRTOP;
```

```
CSlOPO   <- ASlOPO / VSl;
CVSlOPO  <- CSlOPO / PSlOPO*BPROPO;

CSlOPM   <- ASlOPM / VSl;
CVSlOPM  <- CSlOPM / PSlOPM*OPMavgBPR;

# Concentration blood (free and total)
CBlTOP   <- ABlTOP / VBl;
CBlOPO   <- ABlOPO / VBl;
CBlOPM   <- ABlOPM / VBl;

# Concentration in plasma (free and total)
# Concentration in blood / blood-plasma-concentration ratio (BPR)
CPlTOP   <- CBlTOP / BPRTOP;
CPlOPO   <- CBlOPO / BPROPO;
CPlOPM   <- CBlOPM / OPMavgBPR;

# unbound fractions in plasma
# Concentration in plasma multiplied by the unbound fraction in plasma (fup)
CPfTOP  <- CPlTOP * fupTOP;
CPfOPO  <- CPlOPO * fupOPO;
CPfOPM  <- CPlOPM * OPMavgfup;

# Metabolism in plasma according to Michaelis Menten kinetics (see liver metabolism)
AMR5    <- (OPOVmax3 * CPfOPO) / (OPOKm3 + CPfOPO);
# Amount metabolized from OPO to OPM by PON1

# Concentrations in kidney and venous blood from kidney
CKiTOP   <- AKiTOP / VKi;
CVKiTOP  <- CKiTOP / PKiTOP*BPRTOP;

CKiOPO   <- AKiOPO / VKi;
CVKiOPO  <- CKiOPO / PKiOPO*BPROPO;

CKiOPM   <- AKiOPM / VKi;
CVKiOPM  <- CKiOPM / PKiOPM*OPMavgBPR;

# Excretion kidney
ACRTOP  <- CKiTOP * fupTOP * GFR;  # Renal excretion of TOP
ACROPO  <- CKiOPO * fupOPO * GFR;  # Renal excretion of OPO
ACROPM  <- CKiOPM * OPMavgfup * GFR; # Renal excretion of OPM


# DIFFERENTIAL EQUATIONS
# These equation describe the change in the amount (umol) in a specific compartment (d/dt(Amount in compartment))
# This change is defined by:
# - Adding the amount that comes into the compartment (via blood or absorption)
# - Subtracting the amount that is metabolized
# - Subtracting the amount that is leaving the compartment (via venous blood or urine)

# Change in amount in stomach (can be TOP or OPO) is defined as:
# The amount in the stomach multiplied by the negative gastric emptying rate (GER; since the amount is going out of the stomach into the intestine)
```

```
d/dt(Ast) <- -GER * Ast;

# Amount in small intestine compartments
# The intestinal model consists of 7 compartments for the small intestine (in1 to in7), and 1 for the
large intestine (col).
# A fecal (fec) compartment catches what goes through the the whole GIT
# Absorption is limited to the small intestine due to bile acid activity
# Change in the amount present in each compartment depends on:
# - Influx: transition or emtpying rate from previous compartment (stomach or intestinal) multiplied
by the amount in the previous compartment
# - Absorption: absorption rate multiplied by the amount in the present compartment
# - Transition to next compartment: transition rate multiplied by the amount of compound in the
current compartment
d/dt(Ain1) <- GER * Ast - ktSI * Ain1 - ka*Ain1;    # Duodenum, bile acids secreted in duodenum
d/dt(Ain2) <- ktSI * Ain1 - ktSI * Ain2 - ka*Ain2;  # Jejunum
d/dt(Ain3) <- ktSI * Ain2 - ktSI * Ain3 - ka*Ain3;  # Jejunum
d/dt(Ain4) <- ktSI * Ain3 - ktSI * Ain4 - ka*Ain4;  # Ileum
d/dt(Ain5) <- ktSI * Ain4 - ktSI * Ain5 - ka*Ain5;  # Ileum
d/dt(Ain6) <- ktSI * Ain5 - ktSI * Ain6 - ka*Ain6;  # Ileum
d/dt(Ain7) <- ktSI * Ain6 - ktSI * Ain7 - ka*Ain7;  # Ileum, bile acids reabsorbed in ileum
d/dt(Acol) <- ktSI * Ain7 - ktCol * Acol;      # No absorption in the colon (bile acids reabosorbed in
ileum)
d/dt(Afec) <- ktCol * Acol;                    # Amount in feces


# Cumulative amount metabolized in blood
d/dt(TAMR5) <- AMR5;

# Amount in blood
# Here the amount in blood is calculated by adding up the amount of compound in the venous blood of
all compartments
# (multiply tissue blood flow by concentration in venous blood)
# From this amount, the concentration that was already in the blood is subtracted (Summed blood
flows multiplied by the Concentration in blood)
d/dt(ABlTOP) <- QFa * CVFaTOP + QLi * CVLiTOP + QKi * CVKiTOP + QBr * CVBrTOP + QSl
* CVSlTOP + QRi * CVRiTOP - (QFa+QLi+QKi+QBr+QSl+QRi) * CBlTOP;
d/dt(ABlOPO) <- QFa * CVFaOPO + QLi * CVLiOPO + QKi * CVKiOPO + QBr * CVBrOPO +
QSl * CVSlOPO + QRi * CVRiOPO - (QFa+QLi+QKi+QBr+QSl+QRi) * CBlOPO - AMR5;
d/dt(ABlOPM) <- QFa * CVFaOPM + QLi * CVLiOPM + QKi * CVKiOPM + QBr * CVBrOPM +
QSl * CVSlOPM + QRi * CVRiOPM - (QFa+QLi+QKi+QBr+QSl+QRi) * CBlOPM + AMR5;

# Amount in fat
d/dt(AFaTOP) <- QFa * (CBlTOP - CVFaTOP);
d/dt(AFaOPO) <- QFa * (CBlOPO - CVFaOPO);
d/dt(AFaOPM) <- QFa * (CBlOPM - CVFaOPM);

# Cumulative amount metablized in liver
d/dt(TAMR1) <- AMR1;
d/dt(TAMR2) <- AMR2;

d/dt(TAMR3) <- AMR3;
d/dt(TAMR4) <- AMR4;

# Amount in liver
```

```
# The amounts absorbed from the individual small intestine compartments is added (absorption rate constant multiplied by the amount in the specific compartment)
# The amount coming in from the blood is added (Blood flow to liver multiplied by the difference in arterial blood and venous blood)
# The amounts being metabolized are subtracted and added to the appropriate submodel (previously calculated using Michaelis Menten kinetics)
# If the absorbed pesticide is a thiophosphate, it will be absorbed in the liver compartment of the TOP submodel, if it is a phosphate, it will be absorbed in the liver compartment of the OPO submodel
if(TOP == 1 ){
    d/dt(ALiTOP) <- (ka*Ain1 + ka*Ain2 + ka*Ain3 + ka*Ain4 + ka*Ain5 + ka*Ain6 + ka*Ain7) + QLi * (CBlTOP - CVLiTOP) - AMR1 - AMR2;
    d/dt(ALiOPO) <- QLi * (CBlOPO - CVLiOPO) + AMR1 - AMR3 - AMR4;
    d/dt(ALiOPM) <- QLi * (CBlOPM - CVLiOPM) + AMR2 + AMR3 + AMR4;
}else{
    d/dt(ALiTOP) <- QLi * (CBlTOP - CVLiTOP) - AMR1 - AMR2;
    d/dt(ALiOPO) <- (ka*Ain1 + ka*Ain2 + ka*Ain3 + ka*Ain4 + ka*Ain5 + ka*Ain6 + ka*Ain7) + QLi * (CBlOPO - CVLiOPO) + AMR1 - AMR3 - AMR4;
    d/dt(ALiOPM) <- QLi * (CBlOPM - CVLiOPM) + AMR2 + AMR3 + AMR4;
}

# Amount in brain
d/dt(ABrTOP) <- QBr * (CBlTOP - CVBrTOP);
d/dt(ABrOPO) <- QBr * (CBlOPO - CVBrOPO);
d/dt(ABrOPM) <- QBr * (CBlOPM - CVBrOPM);

# Amount in richly perfused tissue
d/dt(ARiTOP) <- QRi * (CBlTOP - CVRiTOP);
d/dt(ARiOPO) <- QRi * (CBlOPO - CVRiOPO);
d/dt(ARiOPM) <- QRi * (CBlOPM - CVRiOPM);


# Amount in slowly perfused tissue
d/dt(ASlTOP) <- QSl * (CBlTOP - CVSlTOP);
d/dt(ASlOPO) <- QSl * (CBlOPO - CVSlOPO);
d/dt(ASlOPM) <- QSl * (CBlOPM - CVSlOPM);

# Cumulative amount excreted into urine
d/dt(TACRTOP) <- ACRTOP;
d/dt(TACROPO) <- ACROPO;
d/dt(TACROPM) <- ACROPM;

# Amount in kidney
# Normal kidney amount minus the amount excreted into urine
d/dt(AKiTOP) <- QKi * (CBlTOP - CVKiTOP) - ACRTOP;
d/dt(AKiOPO) <- QKi * (CBlOPO - CVKiOPO) - ACROPO;
d/dt(AKiOPM) <- QKi * (CBlOPM - CVKiOPM) - ACROPM;
```

5. References

(1) ATDR. Toxicological Profile for Chlorfenvinphos, 1997. https://www.atsdr.cdc.gov/ToxProfiles/tp83.pdf.

(2) Punt, A.; Louisse, J.; Pinckaers, N.; Fabian, E.; van Ravenzwaay, B. Predictive Performance of Next Generation Physiologically Based Kinetic (PBK) Model Predictions in Rats Based on In Vitro and In Silico Input Data. *Toxicological Sciences* **2022**, *186* (1), 18–28. https://doi.org/10.1093/toxsci/kfab150.

(3) Cook, T. J.; Shenoy, S. S. Intestinal Permeability of Chlorpyrifos Using the Single-Pass Intestinal Perfusion Method in the Rat. *Toxicology* **2003**, *184* (2), 125–133. https://doi.org/10.1016/S0300-483X(02)00555-3.

(4) Yu, L. X.; Amidon, G. L. A Compartmental Absorption and Transit Model for Estimating Oral Drug Absorption. *International Journal of Pharmaceutics* **1999**, *186* (2), 119–125. https://doi.org/10.1016/S0378-5173(99)00147-7.

(5) Timchalk, C.; Nolan, R. J.; Mendrala, A. L.; Dittenber, D. A.; Brzak, K. A.; Mattsson, J. L. A Physiologically Based Pharmacokinetic and Pharmacodynamic (PBPK/PD) Model for the Organophosphate Insecticide Chlorpyrifos in Rats and Humans. *Toxicological Sciences* **2002**, *66* (1), 34–53. https://doi.org/10.1093/toxsci/66.1.34.

(6) Nolan, R. J.; Rick, D. L.; Freshour, N. L.; Saunders, J. H. Chlorpyrifos: Pharmacokinetics in Human Volunteers. *Toxicology and Applied Pharmacology* **1984**, *73* (1), 8–15. https://doi.org/10.1016/0041-008X(84)90046-2.

(7) McKim, J. M.; Kolanczyk, R. C.; Lien, G. J.; Hoffman, A. D. Dynamics of Renal Excretion of Phenol and Major Metabolites in the Rainbow Trout (Oncorhynchus Mykiss). *Aquatic Toxicology* **1999**, *45* (4), 265–277. https://doi.org/10.1016/S0166-445X(98)00105-2.

(8) WHO. *Characterization and application of physiologically based pharmacokinetic models in risk assessment*. https://www.who.int/publications-detail-redirect/9789241500906 (accessed 2024-01-02).

(9) Proença, S.; Escher, B. I.; Fischer, F. C.; Fisher, C.; Grégoire, S.; Hewitt, N. J.; Nicol, B.; Paini, A.; Kramer, N. I. Effective Exposure of Chemicals in in Vitro Cell Systems: A Review of Chemical Distribution Models. *Toxicology in Vitro* **2021**, *73*, 105133. https://doi.org/10.1016/j.tiv.2021.105133.

(10) Louisse, J.; Alewijn, M.; Peijnenburg, A. A. C. M.; Cnubben, N. H. P.; Heringa, M. B.; Coecke, S.; Punt, A. Towards Harmonization of Test Methods for *in Vitro* Hepatic Clearance Studies. *Toxicology in Vitro* **2020**, *63*, 104722. https://doi.org/10.1016/j.tiv.2019.104722.

(11) Lobell, M.; Sivarajah, V. In Silico Prediction of Aqueous Solubility, Human Plasma Protein Binding and Volume of Distribution of Compounds from Calculated pKa and AlogP98 Values. *Mol Divers* **2003**, *7* (1), 69–87. https://doi.org/10.1023/B:MODI.0000006562.93049.36.

(12) Berezhkovskiy, L. M. Determination of Volume of Distribution at Steady State with Complete Consideration of the Kinetics of Protein and Tissue Binding in Linear Pharmacokinetics. *J Pharm Sci* **2004**, *93* (2), 364–374. https://doi.org/10.1002/jps.10539.

(13) Rodgers, T.; Rowland, M. Mechanistic Approaches to Volume of Distribution Predictions: Understanding the Processes. *Pharm Res* **2007**, *24* (5), 918–933. https://doi.org/10.1007/s11095-006-9210-3.

(14) Simcyp. *Simcyp Prediction Tools - Blood to Plasma Partition Ratio (B/P)*. https://members.simcyp.com/account/tools/BP/ (accessed 2023-01-18).

(15) Brown, R. P.; Delp, M. D.; Lindstedt, S. L.; Rhomberg, L. R.; Beliles, R. P. Physiological Parameter Values for Physiologically Based Pharmacokinetic Models. *Toxicol Ind Health* **1997**, *13* (4), 407–484. https://doi.org/10.1177/074823379701300401.

(16) Grandoni, S.; Cesari, N.; Brogin, G.; Puccini, P.; Magni, P. Building In-House PBPK Modelling Tools for Oral Drug Administration from Literature Information. *ADMET DMPK* **2019**, *7* (1), 4–21. https://doi.org/10.5599/admet.638.

(17) Stark, H.; Schuster, S. Comparison of Various Approaches to Calculating the Optimal Hematocrit in Vertebrates. *Journal of Applied Physiology* **2012**, *113* (3), 355–367. https://doi.org/10.1152/japplphysiol.00369.2012.
(18) Walton, K.; Dorne, J. L. C. M.; Renwick, A. G. Species-Specific Uncertainty Factors for Compounds Eliminated Principally by Renal Excretion in Humans. *Food and Chemical Toxicology* **2004**, *42* (2), 261–274. https://doi.org/10.1016/j.fct.2003.09.001.
(19) Barter, Z. E.; Bayliss, M. K.; Beaune, P. H.; Boobis, A. R.; Carlile, D. J.; Edwards, R. J.; Houston, J. B.; Lake, B. G.; Lipscomb, J. C.; Pelkonen, O. R.; Tucke, G. T.; Rostami-Hodjegan, A. Scaling Factors for the Extrapolation of In Vivo Metabolic Drug Clearance From In Vitro Data: Reaching a Consensus on Values of Human Micro-Somal Protein and Hepatocellularity Per Gram of Liver. *Current Drug Metabolism* **2007**, *8* (1), 33–45. https://doi.org/10.2174/138920007779315053.
(20) Chiu, W. A.; Ginsberg, G. L. Development and Evaluation of a Harmonized Physiologically Based Pharmacokinetic (PBPK) Model for Perchloroethylene Toxicokinetics in Mice, Rats, and Humans. *Toxicology and Applied Pharmacology* **2011**, *253* (3), 203–234. https://doi.org/10.1016/j.taap.2011.03.020.
(21) Cubitt, H. E.; Houston, J. B.; Galetin, A. Prediction of Human Drug Clearance by Multiple Metabolic Pathways: Integration of Hepatic and Intestinal Microsomal and Cytosolic Data. *Drug Metab Dispos* **2011**, *39* (5), 864–873. https://doi.org/10.1124/dmd.110.036566.
(22) Medinsky, M. A.; Leavens, T. L.; Csanády, G. A.; Gargas, M. L.; Bond, J. A. In Vivo Metabolism of Butadiene by Mice and Rats: A Comparison of Physiological Model Predictions and Experimental Data. *Carcinogenesis* **1994**, *15* (7), 1329–1340. https://doi.org/10.1093/carcin/15.7.1329.
(23) Zhao, S.; Wesseling, S.; Spenkelink, B.; Rietjens, I. M. C. M. Physiologically Based Kinetic Modelling Based Prediction of in Vivo Rat and Human Acetylcholinesterase (AChE) Inhibition upon Exposure to Diazinon. *Arch Toxicol* **2021**, *95* (5), 1573–1593. https://doi.org/10.1007/s00204-021-03015-1.
(24) Tetko, I. V.; Tanchuk, V. Yu. Application of Associative Neural Networks for Prediction of Lipophilicity in ALOGPS 2.1 Program. *J. Chem. Inf. Comput. Sci.* **2002**, *42* (5), 1136–1145. https://doi.org/10.1021/ci025515j.
(25) Pan, X.; Wang, H.; Li, C.; Zhang, J. Z. H.; Ji, C. MolGpka: A Web Server for Small Molecule pKa Prediction Using a Graph-Convolutional Neural Network. *J. Chem. Inf. Model.* **2021**, *61* (7), 3159–3165. https://doi.org/10.1021/acs.jcim.1c00075.
(26) Punt, A.; Pinckaers, N.; Peijnenburg, A.; Louisse, J. Development of a Web-Based Toolbox to Support Quantitative In-Vitro-to-In-Vivo Extrapolations (QIVIVE) within Nonanimal Testing Strategies. *Chem. Res. Toxicol.* **2021**, *34* (2), 460–472. https://doi.org/10.1021/acs.chemrestox.0c00307.
(27) Racke, K. D. Environmental Fate of Chlorpyrifos. In *Reviews of Environmental Contamination and Toxicology*; Ware, G. W., Ed.; Reviews of Environmental Contamination and Toxicology; Springer: New York, NY, 1993; pp 1–150. https://doi.org/10.1007/978-1-4612-4362-5_1.
(28) Biberoglu, K.; Tacal, O.; Schopfer, L. M.; Lockridge, O. Chlorpyrifos Oxon-Induced Isopeptide Bond Formation in Human Butyrylcholinesterase. *Molecules* **2020**, *25* (3), 533. https://doi.org/10.3390/molecules25030533.
(29) Bako, Y. F. R.; Kabore, B.; Tapsoba, I. Electrochemical Sensors Based on Modification of Carbon Fiber Microelectrode by Nickel Phthalocyanine Polymer for 3-Methyl-4-Nitrophenol Analysis in Water. *Materials Sciences and Applications* **2017**, *8* (11), 798–810. https://doi.org/10.4236/msa.2017.811058.
(30) Yang, M.; Zhang, X. Comparative Developmental Toxicity of New Aromatic Halogenated DBPs in a Chlorinated Saline Sewage Effluent to the Marine Polychaete Platynereis Dumerilii. *Environ. Sci. Technol.* **2013**, *47* (19), 10868–10876. https://doi.org/10.1021/es401841t.
(31) Cubitt, H. E.; Houston, J. B.; Galetin, A. Relative Importance of Intestinal and Hepatic Glucuronidation—Impact on the Prediction of Drug Clearance. *Pharm Res* **2009**, *26* (5), 1073–1083. https://doi.org/10.1007/s11095-008-9823-9.

(32) Nolan, R. J.; Dryzga, M. D.; Landenberger, B. D.; Kastl, P. E. Chlorpyrifos: Tissue Distribution and Metabolism of Orally Administered 14C-Labeled Chlorpyrifos in Fischer 344 Rats, 1987. https://www3.epa.gov/pesticides/chem_search/cleared_reviews/csr_PC-059101_1-Jun-88_250.pdf.
(33) Bakke, J. E.; Fell, V. J.; Price, C. E. Rat Urinary Metabolites from O, O-diethyl-O-(3, 5, 6-trichloro-2-pyridyl) Phosphorothioate. *Journal of Environmental Science and Health, Part B* **1976**, *11* (3), 225–230. https://doi.org/10.1080/03601237609372038.
(34) Mücke, Wolfgang.; Alt, K. O.; Esser, H. E. Degradation of Carbon-14 Labeled Diazinon in the Rat. *J. Agric. Food Chem.* **1970**, *18* (2), 208–212. https://doi.org/10.1021/jf60168a020.
(35) Miyamoto, J.; Mihara, K.; Hosokawa, S. Comparative Metabolism of M-Methyl-14C-Sumithion in Several Species of Mammals in Vivo. *日本農薬学会誌（Journal of Pesticide Science）* **1976**, *1* (1), 9–21. https://doi.org/10.1584/jpestics.1.9.
(36) Food and Agriculture Organization of the United Nations; World Health Organization; FAO Panel of Experts on Pesticide Residues in Food and the Environment; WHO Core Assessment Group on Pesticide Residues. Pesticide Residues in Food : 2007, Toxicological Evaluations, Sponsored Jointly by FAO and WHO, with the Support of the International Programme on Chemical Safety, Joint Meeting of the FAO Panel of Experts on Pesticide Residues in Food and the Environment and the WHO Core Assessment Group, Geneva, Switzerland, 18-27 September 2007. *Pesticide residues in food 2007 : evaluations : part 2, toxicological* **2009**, 529.
(37) APVMA. *NRA Review of Parathion-methyl - Toxicology Assessment Section 4: Evaluation of the mammalian toxicology and metabolism/toxicokinetics*. https://www.apvma.gov.au/sites/default/files/publication/14791-parathionmethyl-tox.pdf (accessed 2023-12-19).
(38) Poet, T. S.; Wu, H.; Kousba, A. A.; Timchalk, C. In Vitro Rat Hepatic and Intestinal Metabolism of the Organophosphate Pesticides Chlorpyrifos and Diazinon. *Toxicological Sciences* **2003**, *72* (2), 193–200. https://doi.org/10.1093/toxsci/kfg035.
(39) Zhao, S.; Wesseling, S.; Rietjens, Ivonne. M. C. M.; Strikwold, M. Inter-Individual Variation in Chlorpyrifos Toxicokinetics Characterized by Physiologically Based Kinetic (PBK) and Monte Carlo Simulation Comparing Human Liver Microsome and Supersome^{TM} Cytochromes P450 (CYP)-Specific Kinetic Data as Model Input. *Arch Toxicol* **2022**, *96* (5), 1387–1409. https://doi.org/10.1007/s00204-022-03251-z.
(40) Zhao, S.; Kamelia, L.; Boonpawa, R.; Wesseling, S.; Spenkelink, B.; Rietjens, I. M. C. M. Physiologically Based Kinetic Modeling-Facilitated Reverse Dosimetry to Predict In Vivo Red Blood Cell Acetylcholinesterase Inhibition Following Exposure to Chlorpyrifos in the Caucasian and Chinese Population. *Toxicol Sci* **2019**, *171* (1), 69–83. https://doi.org/10.1093/toxsci/kfz134.
(41) Nallani, G.; Chandrasekaran, A.; Kassahun, K.; Shen, L.; Reiss, R.; Whatling, P. Determination of Enzymatic Kinetics of Metabolism of Dimethoate and Omethoate in Rats and Humans. *Xenobiotica* **2023**, *53* (4), 279–287. https://doi.org/10.1080/00498254.2023.2228904.
(42) Chen, J.; Zhao, S.; Wesseling, S.; Kramer, N. I.; Rietjens, I. M. C. M.; Bouwmeester, H. Acetylcholinesterase Inhibition in Rats and Humans Following Acute Fenitrothion Exposure Predicted by Physiologically Based Kinetic Modeling-Facilitated Quantitative In Vitro to In Vivo Extrapolation. *Environ. Sci. Technol.* **2023**. https://doi.org/10.1021/acs.est.3c07077.
(43) Buratti, F. M.; Volpe, M. T.; Meneguz, A.; Vittozzi, L.; Testai, E. CYP-Specific Bioactivation of Four Organophosphorothioate Pesticides by Human Liver Microsomes. *Toxicology and Applied Pharmacology* **2003**, *186* (3), 143–154. https://doi.org/10.1016/S0041-008X(02)00027-3.
(44) Gearhart, J. M.; Jepson, G. W.; Clewell, H. J.; Andersen, M. E.; Conolly, R. B. Physiologically Based Pharmacokinetic Model for the Inhibition of Acetylcholinesterase by Organophosphate Esters. *Environmental Health Perspectives* **1994**, *102* (suppl 11), 51–60. https://doi.org/10.1289/ehp.94102s1151.

(45) Ma, T.; Chambers, J. E. Kinetic Parameters of Desulfuration and Dearylation of Parathion and Chlorpyrifos by Rat Liver Microsomes. *Food and Chemical Toxicology* **1994**, *32* (8), 763–767. https://doi.org/10.1016/S0278-6915(09)80009-4.
(46) McCracken, N. W.; Blain, P. G.; Williams, F. M. Human Xenobiotic Metabolizing Esterases in Liver and Blood. *Biochemical Pharmacology* **1993**, *46* (7), 1125–1129. https://doi.org/10.1016/0006-2952(93)90459-A.
(47) Mutch, E.; Daly, A. K.; Leathart, J. B. S.; Blain, P. G.; Williams, F. M. Do Multiple Cytochrome P450 Isoforms Contribute to Parathion Metabolism in Man? *Arch Toxicol* **2003**, *77* (6), 313–320. https://doi.org/10.1007/s00204-003-0452-0.
(48) Omwenga, I.; Zhao, S.; Kanja, L.; Mol, H.; Rietjens, I. M. C. M.; Louisse, J. Prediction of Dose-Dependent in Vivo Acetylcholinesterase Inhibition by Profenofos in Rats and Humans Using Physiologically Based Kinetic (PBK) Modeling-Facilitated Reverse Dosimetry. *Arch Toxicol* **2021**, *95* (4), 1287–1301. https://doi.org/10.1007/s00204-021-03004-4.
(49) Smith, J. N.; Wang, J.; Lin, Y.; Klohe, E. M.; Timchalk, C. Pharmacokinetics and Pharmacodynamics of Chlorpyrifos and 3,5,6-Trichloro-2-Pyridinol in Rat Saliva After Chlorpyrifos Administration. *Toxicological Sciences* **2012**, *130* (2), 245–256. https://doi.org/10.1093/toxsci/kfs251.
(50) Timchalk, C.; Poet, T. S.; Hinman, M. N.; Busby, A. L.; Kousba, A. A. Pharmacokinetic and Pharmacodynamic Interaction for a Binary Mixture of Chlorpyrifos and Diazinon in the Rat. *Toxicology and Applied Pharmacology* **2005**, *205* (1), 31–42. https://doi.org/10.1016/j.taap.2004.09.004.
(51) Smith, J. N.; Campbell, J. A.; Busby-Hjerpe, A. L.; Lee, S.; Poet, T. S.; Barr, D. B.; Timchalk, C. Comparative Chlorpyrifos Pharmacokinetics via Multiple Routes of Exposure and Vehicles of Administration in the Adult Rat. *Toxicology* **2009**, *261* (1), 47–58. https://doi.org/10.1016/j.tox.2009.04.041.
(52) EPA. *Data Evaluation Record for Chlorpyrifos*; 2009. https://archive.epa.gov/osa/hsrb/web/pdf/1d5-science-rvw-kisicki-052709.pdf (accessed 2024-03-28).
(53) Garfitt, S. J.; Jones, K.; Mason, H. J.; Cocker, J. Exposure to the Organophosphate Diazinon: Data from a Human Volunteer Study with Oral and Dermal Doses. *Toxicology Letters* **2002**, *134* (1), 105–113. https://doi.org/10.1016/S0378-4274(02)00178-9.
(54) APVMA. The Reconsideration of Approvals of the Active Constituent Diazinon, Registrations of Products Containing Diazinon and Approval of Their Associated Labels, 2006.
(55) Lu, C.; Irish, R.; Fenske, R. Biological Monitoring of Diazinon Exposure Using Saliva in an Animal Model. *Journal of Toxicology and Environmental Health, Part A* **2003**, *66* (24), 2315–2325. https://doi.org/10.1080/716100640.
(56) Wu, H.; Evreux-Gros, C.; Descotes, J. Influence of Cimetidine on the Toxicity and Toxicokinetics of Diazinon in the Rat. *Hum Exp Toxicol* **1996**, *15* (5), 391–395. https://doi.org/10.1177/096032719601500505.
(57) Reiss, R.; Loccisano, A.; Deines, A.; Kim, M.; Nallani, G.; Chandrasekaran, A.; Whatling, P. A Physiologically-Based Pharmacokinetic/Pharmacodynamic (PBPK/PD) Model for the Insecticide Dimethoate. *Xenobiotica* **2023**, *0* (0), 1–14. https://doi.org/10.1080/00498254.2023.2258507.
(58) Meaklim, J.; Yang, J.; Drummer, O. H.; Killalea, S.; Staikos, V.; Horomidis, S.; Rutherford, D.; Ioannides, -Demos Lisa L.; Lim, S.; McLean, A. J.; McNeil, J. J. Fenitrothion: Toxicokinetics and Toxicologic Evaluation in Human Volunteers. *Environmental Health Perspectives* **2003**, *111* (3), 305–308. https://doi.org/10.1289/ehp.5726.
(59) Nosál', M.; Hladká, A. Determination of the Exposure to Fenitrothion (0,0-Dimethyl-0/3-Methyl-4-Nitrophenyl/Thiophosphate) on the Basis of the Excretion of p-Nitro-m-Cresol by the Urine of the Persons Tested. *Int. Arch. Gewerbepath. Gewerbehyg.* **1968**, *25* (1), 28–38. https://doi.org/10.1007/BF00404665.

(60) Miyamoto, J. Studies on the Mode of Action of Organophosphorus Compounds. *Agricultural and Biological Chemistry* **1964**, *28* (7), 411–430. https://doi.org/10.1080/00021369.1964.10858259.
(61) Becker, J. M.; Nakatsugawa, T. Significance of Hepatic Breakthrough Thresholds in Fenitrothion Toxicity in Male Rats and Female Mice. *Pesticide Biochemistry and Physiology* **1990**, *38* (1), 34–40. https://doi.org/10.1016/0048-3575(90)90145-R.
(62) Hladká, A.; Nosál', M. The Determination of the Exposition to Metathion (Fenitrotion) on the Basis of Excreting Its Metabolite p-Nitro-m-Cresol through Urine in Rats. *Int. Archiv für Gewerbepathologie und Gewerbehygiene* **1967**, *23* (3), 209–214. https://doi.org/10.1007/BF00368120.
(63) Kramer, R. E.; Wellman, S. E.; Rockhold, R. W.; Baker, R. C. Pharmacokinetics of Methyl Parathion: A Comparison Following Single Intravenous, Oral or Dermal Administration. *JBS* **2002**, *9* (4), 311–320. https://doi.org/10.1159/000065001.
(64) Eigenberg, D. A.; Pazdernik, T. L.; Doull, J. Hemoperfusion and Pharmacokinetic Studies with Parathion and Paraoxon in the Rat and Dog. *Drug Metab Dispos* **1983**, *11* (4), 366–370.
(65) Hurh, E.; Lee, E. J.; Kim, Y. G.; Kim, S. Y.; Kim, S. H.; Kim, Y. C.; Lee, M. G. Effects of Physostigmine on the Pharmacokinetics of Intravenous Parathion in Rats. *Biopharmaceutics & Drug Disposition* **2000**, *21* (8), 331–338. https://doi.org/10.1002/bdd.243.
(66) Hutson, D. H.; Hathway, D. E. Toxic Effects of Chlorfenvinphos in Dogs and Rats. *Biochemical Pharmacology* **1967**, *16* (6), 949–962. https://doi.org/10.1016/0006-2952(67)90267-5.
(67) Ikeda, T.; Kojima, T.; Yoshida, M.; Takahashi, H.; Tsuda, S.; Shirasu, Y. Pretreatment of Rats with an Organophosphorus Insecticide, Chlorfenvinphos, Protects against Subsequent Challenge with the Same Compound. *Fundamental and Applied Toxicology* **1990**, *14* (3), 560–567. https://doi.org/10.1016/0272-0590(90)90260-Q.
(68) Ikeda, T.; Tsuda, S.; Shirasu, Y. Pharmacokinetic Analysis of Protection by an Organophosphorus Insecticide, Chlorfenvinphos, against the Toxicity of Its Succeeding Dosage in Rats. *Fundamental and Applied Toxicology* **1992**, *18* (2), 299–306. https://doi.org/10.1016/0272-0590(92)90059-Q.
(69) Cho, Y.-J.; Min, K.-J.; Lee, I.-S.; Cha, C.-G. Determination of Urinary Metabolite of Profenofos after Oral Administration and Dermal Application to Rats. *Journal of Food Hygiene and Safety* **2002**, *17* (1), 20–25.
(70) Becker, J. M.; Nakatsugawa, T. Hepatic Breakthrough Thresholds for Parathion and Paraoxon and Their Implications to Toxicity in Normal and DDE-Pretreated Rats. *Pesticide Biochemistry and Physiology* **1990**, *36* (1), 83–98. https://doi.org/10.1016/0048-3575(90)90025-W.
(71) Johnson, K. A.; Goody, R. S. The Original Michaelis Constant: Translation of the 1913 Michaelis-Menten Paper. *Biochemistry* **2011**, *50* (39), 8264–8269. https://doi.org/10.1021/bi201284u.
(72) Rietjens, I. M. C. M.; Louisse, J.; Punt, A. Tutorial on Physiologically Based Kinetic Modeling in Molecular Nutrition and Food Research. *Molecular Nutrition & Food Research* **2011**, *55* (6), 941–956. https://doi.org/10.1002/mnfr.201000655.
(73) Wang, W.; Hallow, K.; James, D. A Tutorial on RxODE: Simulating Differential Equation Pharmacometric Models in R. *CPT: Pharmacometrics & Systems Pharmacology* **2016**, *5* (1), 3–10. https://doi.org/10.1002/psp4.12052.
